# Genetics of monozygotic twins reveals the impact of environmental sensitivity on psychiatric and neurodevelopmental phenotypes

**DOI:** 10.1038/s41562-025-02193-7

**Published:** 2025-06-10

**Authors:** Elham Assary, Jonathan R. I. Coleman, Gibran Hemani, Margot P. van de Weijer, Laurence J. Howe, Teemu Palviainen, Katrina L. Grasby, Rafael Ahlskog, Marianne Nygaard, Rosa Cheesman, Kai Lim, Chandra A. Reynolds, Juan R. Ordoñana, Lucia Colodro-Conde, Scott Gordon, Juan J. Madrid-Valero, Anbupalam Thalamuthu, Jouke-Jan Hottenga, Jonas Mengel-From, Nicola J. Armstrong, Perminder S. Sachdev, Teresa Lee, Henry Brodaty, Julian N. Trollor, Margaret Wright, David Ames, Vibeke S. Catts, Antti Latvala, The Within Family Consortium, Robin G. Walters, Sam Morris, Zhengming Chen, Kuang Lin, Amanda M. Hughes, Iona Y. Millwood, Liming Li, Alexandra Havdahl, Jean-Baptiste Pingault, W. David Hill, Michel Boivin, Daniel J. Benjamin, Matthew C. Keller, Fartein A. Torvik, Shuai Li, Eco de Geus, Floris Huider, Wonu Akingbuwa, Helga Ask, Per Magnus, Bjørn Olav Åsvold, Sonia Brescianini, Alexandros Giannelis, Emily A. Willoughby, Joohon Sung, Soo Ji Lee, Hyojin Pyun, David Evans, Campbell Archie, Eero Vuoksimaa, Travis Mallard, K. Paige Harden, Elliot M. Tucker-Drob, Sven Oskarsson, Christopher J. Hammond, Kaare Christensen, Mark Taylor, Sebastian Lundström, Henrik Larsson, Robert Karlsson, Nancy L. Pedersen, Karen A. Mather, Sarah E. Medland, Dorret I. Boomsma, Nicholas G. Martin, Robert Plomin, Meike Bartels, Paul Lichtenstein, Jaakko Kaprio, Thalia C. Eley, Neil M. Davies, Patricia B. Munroe, Robert Keers

**Affiliations:** 1 School of Biological and Behavioural Sciences, Queen Mary University of London, London, UK; 2 Social, Genetic and Developmental Psychiatry Centre, Institute of Psychiatry, Psychology and Neuroscience, King’s College London, London, UK; 3 NIHR Maudsley Biomedical Research Centre, South London and Maudsley NHS Foundation Trust, London, UK; 4 Medical Research Council Integrative Epidemiology Unit, University of Bristol, Bristol, UK; 5 Department of Biological Psychology, Vrije Universiteit Amsterdam, Amsterdam, the Netherlands; 6 Amsterdam Public Health Institute, Amsterdam University Medical Center, Amsterdam, the Netherlands; 7 Institute for Molecular Medicine Finland, University of Helsinki, Helsinki, Finland; 8 QIMR Berghofer Medical Research Institute, Brisbane, Queensland Australia; 9 School of Biomedical Sciences, Queensland University of Technology, Brisbane, Queensland Australia; 10 School of Biomedical Sciences, University of Queensland, Brisbane, Queensland Australia; 11 Department of Government, Uppsala University, Uppsala, Sweden; 12 The Danish Twin Registry, Department of Public Health, University of Southern Denmark, Odense, Denmark; 13 Department of Clinical Genetics, Odense University Hospital, Odense, Denmark; 14 The PROMENTA Research Center, Department of Psychology, University of Oslo, Oslo, Norway; 15 Institute for Behavioral Genetics, University of Colorado Boulder, Boulder, CO USA; 16 Department of Psychology, University of California, Riverside, Riverside, CA USA; 17 Department of Psychology and Neuroscience, University of Colorado Boulder, Boulder, CO USA; 18 Department of Human Anatomy and Psychobiology, University of Murcia, Murcia, Spain; 19 Biomedical Research Institute of Murcia (IMIB-Arrixaca), Murcia, Spain; 20 School of Psychology, University of Queensland, Brisbane, Queensland Australia; 21 Centre for Healthy Brain Ageing, Discipline of Psychiatry and Mental Health, School of Clinical Medicine, Faculty of Medicine and Health, UNSW Sydney, Sydney, New South Wales Australia; 22 School of Electrical Engineering, Computing and Mathematical Sciences, Curtin University, Perth, Western Australia Australia; 23 National Centre of Excellence in Intellectual Disability Health, Faculty of Medicine and Health, UNSW Sydney, Sydney, New South Wales Australia; 24 Queensland Brain Institute, University of Queensland, Brisbane, Queensland Australia; 25 University of Melbourne Academic Unit for Psychiatry of Old Age, St George’s Hospital, Melbourne, Victoria Australia; 26 Institute of Criminology and Legal Policy, University of Helsinki, Helsinki, Finland; 27 Department of Psychiatry, Harvard Medical School, Boston, MA USA; 28 Center for Precision Psychiatry, Department of Psychiatry, Massachusetts General Hospital, Boston, MA USA; 29 Department of Psychology and Population Research Center, University of Texas at Austin, Austin, TX USA; 30 Department of Twin Research and Genetic Epidemiology, King’s College London, London, UK; 31 Department of Clinical Biochemistry, Odense University Hospital, Odense, Denmark; 32 Department of Medical Epidemiology and Biostatistics, Karolinska Institutet, Solna, Sweden; 33 Gillberg Neuropsychiatry Centre, University of Gothenburg, Gothenburg, Sweden; 34 Office for Psychiatry, Habilitation and Aid, Child and Adolescent Mental Health Services, Malmö, Sweden; 35 Department of Complex Trait Genetics, Center for Neurogenomics and Cognitive Research, Amsterdam Neuroscience, Vrije Universiteit Amsterdam, Amsterdam, the Netherlands; 36 Amsterdam Reproduction and Development research institute, Amsterdam, the Netherlands; 37 Division of Psychiatry, University College London, London, UK; 38 Department of Statistical Science, University College London, London, UK; 39 William Harvey Research Institute, Queen Mary University of London, London, UK; 40 Nuffield Department of Population Health, University of Oxford, Oxford, UK; 41 Centre for Academic Mental Health, University of Bristol, Bristol, UK; 42 Department of Epidemiology and Biostatistics, School of Public Health, Peking University, Beijing, China; 43 Peking University Center for Public Health and Epidemic Preparedness and Response, Beijing, China; 44 Key Laboratory of Epidemiology of Major Diseases (Peking University), Ministry of Education, Beijing, China; 45 PsychGen Centre for Genetic Epidemiology and Mental Health, Norwegian Institute of Public Health, Oslo, Norway; 46 Department of Clinical, Educational and Health Psychology, Division of Psychology and Language Sciences, University College London, London, UK; 47 Department of Psychology, University of Edinburgh, Edinburgh, Scotland; 48 École de Psychologie, Université Laval, Québec City, Québec Canada; 49 Groupe de Recherche sur l’Inadaptation Psychosociale, Québec City, Québec Canada; 50 Behavioral Decision Making Area, Anderson School of Management, University of California, Los Angeles, Los Angeles, CA USA; 51 Human Genetics Department, David Geffen School of Medicine, University of California, Los Angeles, Los Angeles, CA USA; 52 National Bureau of Economic Research, Cambridge, MA USA; 53 Centre for Fertility and Health, Norwegian Institute of Public Health, Oslo, Norway; 54 The PROMENTA Research Center, University of Oslo, Oslo, Norway; 55 Centre for Epidemiology and Biostatistics, Melbourne School of Population and Global Health, University of Melbourne, Melbourne, Victoria Australia; 56 Department of Precision Medicine, School of Clinical Sciences at Monash Health, Monash University, Melbourne, Victoria Australia; 57 Netherlands Twin Register, Department of Biological Psychology, Vrije Universiteit Amsterdam, Amsterdam, the Netherlands; 58 HUNT Center for Molecular and Clinical Epidemiology, Department of Public Health and Nursing, Norwegian University of Science and Technology, Trondheim, Norway; 59 Department of Endocrinology, Clinic of Medicine, St. Olav’s Hospital, Trondheim University Hospital, Trondheim, Norway; 60 Center for Behavioral Science and Mental Health, Istituto Superiore di Sanità, Rome, Italy; 61 Department of Psychology, University of Minnesota Twin Cities, Minneapolis, MN USA; 62 Genome and Health Big Data Laboratory, Department of Public Health, Graduate School of Public Health, Seoul National University, Seoul, South Korea; 63 Institute of Health and Environment, Seoul National University, Seoul, South Korea; 64 Institute for Molecular Biosciences, University of Queensland, Brisbane, Australia; 65 Centre for Genomc and Experimental Medicine, Institute of Genetics & Cancer, University of Edinburgh, Edinburgh, United Kingdom

**Keywords:** Human behaviour, Quantitative trait loci, Genome-wide association studies

## Abstract

Individual sensitivity to environmental exposures may be genetically influenced. This genotype-by-environment interplay implies differences in phenotypic variance across genotypes, but these variants have proven challenging to detect. Genome-wide association studies of monozygotic twin differences are conducted through family-based variance analyses, which are more robust to the systemic biases that impact population-based methods. We combined data from 21,792 monozygotic twins (10,896 pairs) from 11 studies to conduct one of the largest genome-wide association study meta-analyses of monozygotic phenotypic differences, in children, adolescents and adults separately, for seven psychiatric and neurodevelopmental phenotypes: attention deficit hyperactivity disorder symptoms, autistic traits, anxiety and depression symptoms, psychotic-like experiences, neuroticism and wellbeing. The proportions of phenotypic variance explained by single-nucleotide polymorphisms in these phenotypes were estimated (*h*^2^ = 0–18%), but were imprecise. We identified 13 genome-wide significant associations (single-nucleotide polymorphisms, genes and gene sets), including genes related to stress reactivity for depression, growth factor-related genes for autistic traits and catecholamine uptake-related genes for psychotic-like experiences. This is the largest genetic study of monozygotic twins to date by an order of magnitude, evidencing an alternative method to study the genetic architecture of environmental sensitivity. The statistical power was limited for some analyses, calling for better-powered future studies.

## Abstract (web summary)

This study uncovered genetic associations with environmental sensitivity in psychiatric and neurodevelopmental traits in an international collaboration using data from more than 21,000 monozygotic twins—the largest genetic study of monozygotic twin differences to date.

## Main

Complex phenotypes are likely to be affected by genetic and environmental factors and their interactions. Interactions between genetic variants and the environment increase phenotypic variability^1,2^, which may be reflected in differences in the mean and/or variance of a phenotype in a population. This is evidenced when a genotype is associated with phenotype levels only under certain environmental conditions. Environmental sensitivity can also increase the variance of a trait if a genotype produces a wide range of phenotypes depending on environmental exposures, which may or may not also affect its population mean. Genetic knowledge of environmental sensitivity is most consistently exploited in bioengineering and evidenced in behavioural ecology, but it has been extremely challenging to evaluate in humans, especially for psychiatric disorders^3^. Understanding the genetic basis of environmental sensitivity is crucial for improving human health, as it informs on the biological pathways implicated in variations in responses to environmental exposures.

In contrast with most GWASs, which estimate associations of genetic variants and phenotypic means, Genome-wide variance quantitative trait locus (vQTL) analysis^4,5^ aims to discover genetic variants associated with phenotypic variance, which can be prioritized for a statistical test of gene–environment interaction^6^. However, phenotypic variance may be affected not only by gene–environment interactions^1^, but also by selection^7^, phantom vQTLs^8^ and epistasis^4^. It is therefore challenging to robustly determine which potential mechanisms have given rise to the observed trait variance associated with a vQTL. Furthermore, commonly used population-based methods for estimating genetic effects using unrelated individuals suffer from variance inflation^9^, bias due to insufficient correction of demographic and indirect genetic effects^10^, and unstable test statistics for vQTLs when tested loci are in linkage disequilibrium with additive effects^11^. Although statistical correction for certain observed demographic effects (for example, age and sex) is possible, unobserved confounders (for example, residual population stratification, dynastic effects via parents, and assortative mating) cannot be corrected for.

GWASs of monozygotic (MZ) twin differences provide an alternative, family-based approach to estimating vQTLs that is less susceptible to these sources of bias^12^ and can therefore more reliably identify variants that reflect environmental sensitivity.

MZ twins are nearly identical genetically. Therefore, within-pair phenotypic differences are probably due to chance or the environment^13^. Somatic mutations may also play a role in MZ differences for chromosomal and rare diseases^14^, but are unlikely to play a systematic role in common and polygenic complex traits^15^. Although all MZ twin pairs have the same degree of genetic similarity, they have varying degrees of phenotypic similarity. Greater within-pair differences in a population of MZ twins therefore reflect the pairs’ greater sensitivity to their non-shared environments. Jinks and Fulker^16^ proposed a test in MZ twins for which the association between MZ pair differences and MZ pair mean score is obtained, and they provided a full description of its standard biometrical terms. The rationale behind their test of genotype–environment interaction is the same one that underlies tests involving inbred lines of animals that detect genotype–environment interaction through the heterogeneity of within-strain variances. We may now take single-nucleotide polymorphisms (SNPs) as measured genotype indicators and test for associations with within-pair differences.

**Fig. 1:**
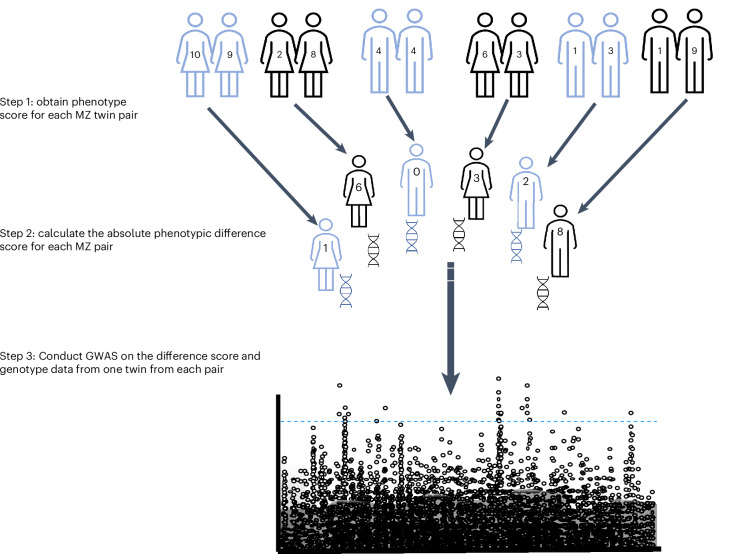
GWAS of MZ differences approach. These analyses are conducted in three main steps: First, a quantitative phenotype value is obtained for a population of MZ twin pairs. Second, the absolute phenotypic difference score is calculated for each MZ pair. This score reflects phenotypic differences due to environmental effects, as the environment makes genetically identical twins diverge phenotypically. The absolute phenotypic difference is corrected for age, sex, ten genetic principal components and any study-specific covariates. The residuals are standardized and inverse rank transformed. Third, a GWAS of the MZ differences score is conducted, using the phenotypic differences score for one twin from each pair and their genotype data; therefore, the sample comprises unrelated individuals.

A GWAS of MZ phenotypic differences can identify the loci associated with variations in environmental sensitivity while also controlling for dynastic and epistatic effects, which are difficult to account for in population-based approaches (Fig. 1 provides a schematic of a GWAS of MZ differences). Although this approach has been advocated for because it facilitates understanding of the genetics of environmental sensitivity^12^, the requirement for a large sample of MZ twins has been a major impediment to progress in this field. In this Article, we report the findings from a GWAS meta-analysis of MZ differences for seven psychological phenotypes, using data from up to 21,792 MZ twins (10,896 pairs) from 11 studies. This is the largest GWAS conducted on MZ twin differences to date, representing an order of magnitude more participants than two previous MZ twin differences GWAS^17,18^. We conducted meta-analyses separately for children, adolescents and adults and identified 13 genome-wide significant associations across the phenotypes studied. This enabled us to estimate the SNP heritability (that is, the proportion of a trait’s variance that can be attributed to genetic variation explained by SNPs) of environmental sensitivity to various mental health phenotypes (adolescent attention deficit hyperactivity disorder (ADHD): *h*^2^ = 0.18 and s.e. = 0.11; child ADHD: *h*^2^ = 0.04 and s.e. = 0.06; adult autistic traits: *h*^2^ = 0.09 and s.e. = 0.15; depression: *h*^2^ = 0.03 and s.e. = 0.09). We also found that higher genetic liability to depression was associated with greater environmental sensitivity to depression in adults.

## Results

**Fig. 2:**
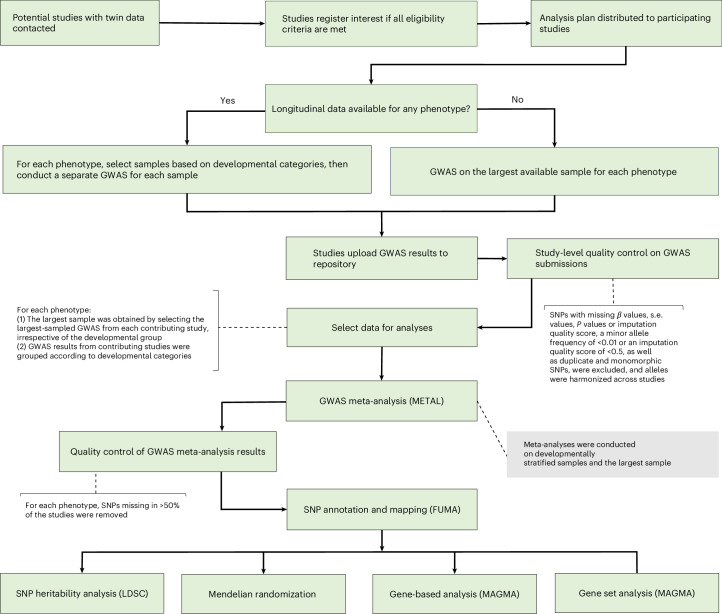
Flow chart of the current study. Flow chart genome-wide association meta-analysis of MZ twin differences in the present study. The eligibility criteria and developmental categories are described in the Methods. If multiple data points were available during the same developmental stage (for example, ages 7, 8 and 11 years), the largest sample was selected. Total *n* values per phenotype (representing the total across participating studies) were as follows: 13,738 (ADHD largest), 13,738 (ADHD child), 7,840 (ADHD adolescent), 12,354 (anxiety largest), 10,494 (anxiety child), 7,932 (anxiety adult), 14,152 (autistic traits largest), 13,130 (autistic traits child), 6,050 (autistic traits adult), 21,792 (depression largest), 10,510 (depression child), 18,074 (depression adult), 8,900 (neuroticism largest), 3,636 (psychotic-like experiences largest) and 13,740 (wellbeing largest). *n* values for GWAS analyses were halved as only data from one twin from each pair were included.

Our empirical analyses included a GWAS meta-analysis, gene-based and gene set analyses and Mendelian randomization analyses (Fig. 2 provides a flow chart for the study). Authors of contributing studies were asked to conduct GWASs of MZ differences separately for samples from child, adolescent and adult twins if repeated measures across lifespan were available. The study-level GWAS results were subjected to quality control and harmonization (Supplementary Information section 2.2) using EasyQC (version 23.8)^19^, then meta-analysed using the inverse-variance-weighted fixed-effect meta-analysis method in METAL (2011 release)^20^. GWAS meta-analyses were conducted using the largest available sample from each study and separately in developmentally stratified samples (Methods). GWAS meta-analysis results were quality controlled (Methods), then used for gene-based and gene set analyses using MAGMA (version 1.08) in the FUMA web application (version 1.5.2)^21^ and SNP heritability analyses using LDSC (version 1.0.1)^22^. We used Mendelian randomization to estimate the effects of psychological phenotypes (as reflected in GWAS associations with means) on phenotypic variance (Methods).

### Descriptives

Table 1 shows descriptive statistics of the GWAS meta-analysis samples per phenotype. Supplementary Information Section 1.1 and the Supplementary Data present descriptives for all of the participating studies. The largest sample was for depression symptoms, with 21,792 MZ twins from 11 studies. The smallest was for psychotic-like experiences (PLEs; 3,636 twins from two cohorts). Mean MZ correlations across studies ranged between *r* = 0.31 for wellbeing and *r* = 0.71 for child ADHD. Overall, within-twin MZ correlations tended to be lower in samples from adult twins than those from child twins.

**Table 1:** Descriptive statistics of the samples in the current study

Phenotype	Sample	*n*_studies_^a^	*n*_MZ twins_^b^	*n*_female_	Mean age (years)	Mean *r*_MZ_^c^
ADHD symptoms	Largest^d^	4	13,738	7,420	9.54	0.71
	Child^e^	4	13,738	7,420	9.54	0.71
	Adolescent^f^	4	7,840	4,821	20.28	0.58
Anxiety symptoms	Largest	5	12,354	6,802	20.40	0.52
	Child	3	10,494	5,881	10.01	0.54
	Adult^g^	5	7,932	5,187	33.35	0.46
Autistic traits	Largest	4	14,152	7,779	15.79	0.63
	Child	3	13,130	7,084	10.22	0.69
	Adult	3	6,050	3,567	22.53	0.63
Depression symptoms	Largest	11	21,792	13,011	43.23	0.41
	Child	3	10,510	5,726	10.05	0.51
	Adult	11	18,074	11,780	43.62	0.38
Neuroticism	Largest	4	8,900	4,096	35.05	0.35
PLEs	Largest	2	3,636	2,135	15.91	0.46
Wellbeing	Largest	9	13,740	8,009	41.30	0.31

^a^*n*_studies_ represents the number of contributing datasets for each phenotype.

^b^GWAS *n* was halved as only genotype data from one twin from each pair were used.^c^Mean *r*_MZ_ represents the mean MZ twin correlation across studies.

^d^For ADHD symptoms, the largest sample was the sample from children. In general, this category represents the largest available sample, which was obtained by selecting the largest sample from each study, irrespective of age group.

^e^The child category includes data from studies in which participants were aged 5–12 years.^f^The adolescent category includes data from studies in which participants were aged 13–18 years.

^g^The adult category includes data from studies in which participants were aged >18 years.

Data from individual studies are reported in the Supplementary Data.

### MZ GWAS meta-analysis

**Fig. 3:**
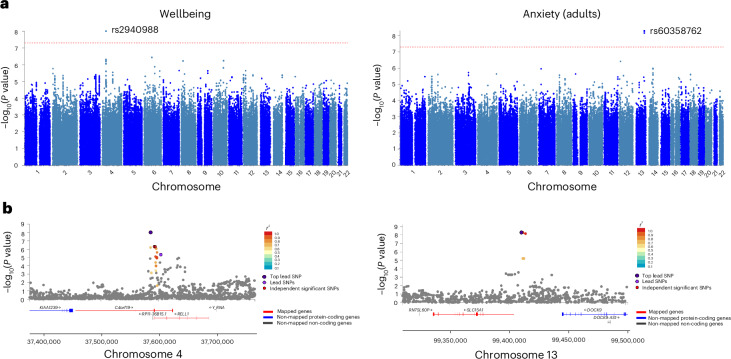
Manhattan and regional plots of genome-wide significant SNPs. **a**, Manhattan plots based on GWASs of MZ differences for wellbeing (left) and anxiety in samples from adults (right). The red dashed lines indicate the genome-wide significant threshold of *P* = 5 × 10^−8^, corrected for multiple testing. **b**, Regional plots of independent significant SNPs for wellbeing (left; rs2940988; *β* = −0.16; *P* = 9.93 × 10^−9^) and anxiety (right; rs60358762; *β*  = 0.44; *P* = 5.07 × 10^−9^). SNPs not in linkage disequilibrium with significant independent lead SNPs in the selected region are coloured grey.

Meta-analyses for each phenotype identified two genome-wide significant variants (Table 2): one associated with variability in wellbeing (rs2940988; *P* = 9.93 × 10^−9^), located in the intronic region of the protein-coding chromosome 4 open reading frame 19 (*C4orf19*) gene; and one (rs60358762; *P* = 5.07 × 10^−9^) associated with variance in anxiety symptoms in adults, located in the intergenic region of the protein-coding *SLC15A1* gene on chromosome 13. Manhattan plots, quantile–quantile (QQ) plots and the genomic regions of these genome-wide significant variants are presented in Fig. 3. No variants were genome-wide significantly associated with variance in the other phenotypes (Supplementary Table 4 provides the top SNPs for each phenotype). Supplementary Figs. 7–11 provide Manhattan and QQ plots for all of the tested phenotypes.

**Table 2:** Genome-wide significant results

GWAS meta-analyses													
**SNP based analysis**													
**Phenotype**	**Sample**	**SNP**	**Chromosome**	**Position**	**Gene**	**A1**		**EAF**	***β***	**s.e.**	***P***	***n***	**Effect across studies**^**a**^
Anxiety	Adult	rs60358762	13	99,411,217	*SLC15A1*	A		0.03	0.44	0.1	5.07 × 10^−9^	3,033	+++??
Wellbeing	Adult	rs2940988	4	37,586,376	*C4orf19*	T		0.88	0.16	0.03	9.93 × 10^−9^	6,464	++?++−+++
**Gene-based analysis**													
**Phenotype**	**Sample**	**Gene**		**Chromosome**	***n***_SNPs_	***n***_parameters_		***n***		***Z*** **statistic**			***P***
Anxiety	Largest	*C15orf38*		15	28	7		5,265		5.08			2.00 × 10^−7b^
Depression	Largest	*PTCH1*		9	130	12		10,166		4.63			1.80 × 10^−6c^
**Gene set analysis**													
**Phenotype**	**Sample**	**Gene set**			***n***_genes_	***β***	**s.d.**	**s.e.**		***P***			***P***_**Bonferroni**_^**d**^
Autistic traits	Child	Genes downregulated in embryonic fibroblasts upon stimulation with TGFβ1 for 1 h			5	1.68	0.03	0.34		6.00 × 10^−7^			0.01
	Adult	Regulation of protein localization to cilium			7	1.43	0.03	0.26		1.00 × 10^−8^			0.0002
Depression	Child	Proteasome regulatory particle			19	0.79	0.03	0.17		1.41 × 10^−6^			0.02
		Proteasome degradation			50	0.52	0.03	0.11		2.48 × 10^−6^			0.04
Neuroticism	Adult	Gemini of coiled bodies			9	1.01	0.02	0.20		2.00 × 10^−7^			0.003
		Negative regulation of vasculature development			92	0.38	0.03	0.08		5.97 × 10^−7^			0.01
PLEs	Adolescent	Regulation of dopamine uptake involved in synaptic transmission			8	1.37	0.03	0.28		5.00 × 10^−7^			0.01
		Catecholamine uptake involved in synaptic transmission			11	1.23	0.03	0.26		9.55 × 10^−7^			0.01
		Extrinsic component of endoplasmic reticulum membrane			5	1.63	0.03	0.35		1.59 × 10^−6^			0.02

^a^A question mark indicates that the SNP was missing in the study, − or + sign indicate whether the direction of effect for each study was the opposite of, or consistent with the direction of effect from meta-analysis.

^b^The Bonferroni-corrected *P* value significance threshold was *P* = 0.05/18,535 = 2.698 × 10^−6^.

^c^The Bonferroni-corrected *P* value significance threshold was *P* = 0.05/18,624 = 2.685 × 10^−6^.

^d^*P*_Bonferroni_ represents the Bonferroni-adjusted *P* value for multiple testing correction. *P* < 5 × 10^−8^ was the significance threshold used to adjust for multiple comparisons when identifying genome-wide significant SNPs.

A1, effect allele; β, beta estimate from linear regression; EAF, average effect allele frequency across studies.

### Gene-based and gene set analyses

The MAGMA gene-based analysis (Methods) found several genes associated with phenotypic variability; however, only two associations passed the threshold for Bonferroni correction for multiple testing (Table 2). The patched 1 (*PTCH1*) gene was associated with variance in depression (*P* = 1.80 × 10^−6^) and the chromosome 15 open reading frame 38 (*C15orf38*) gene was associated with variance in anxiety symptoms (*P* = 2.00 × 10^−7^). Supplementary Figs. 12–16 and Supplementary Table 6 provide the top genes per phenotype.

The MAGMA competitive test of gene sets (Methods) identified nine significant associations after Bonferroni correction for multiple testing (Table 2). Two gene sets were significantly associated with variance in depression symptoms, two with neuroticism, three with PLEs and two with autistic traits (one in samples from adults and one in samples from children). Supplementary Table 7 provides the top gene sets per phenotype, Supplementary Tables 8–11 provide details of significant gene sets and Supplementary Table 12 provides biological annotations for all genome-wide significant results.

### SNP heritability

We estimated the SNP heritability of MZ differences using LDSC (version 1.0.1)^22^ (Methods). The SNP heritability estimate of environmental sensitivity to ADHD symptoms in the samples from adolescents was 0.18 (s.e. = 0.11); the estimate in the samples from children was 0.04 (s.e. = 0.06). The SNP heritability estimate for environmental sensitivity to adult autistic traits was 0.09 (s.e. = 0.15) and for depression symptoms it was 0.03 (s.e. = 0.09 in children and s.e. = 0.06 in adults). All estimates, including those from the remaining phenotypes, were not statistically significant (Supplementary Table 13). We could not estimate the genetic correlation (r_g_) between all phenotypes because the SNP heritabilities were too low and imprecise, except for child and adolescent symptoms of ADHD (*r*_g_ = 0.82; s.e. = 0.56; *P* = 0.15). Overall, despite this being one of the largest samples of MZ twins to date, low power resulted in large confidence intervals around the heritability estimates.

### Mendelian randomization

**Fig. 4:**
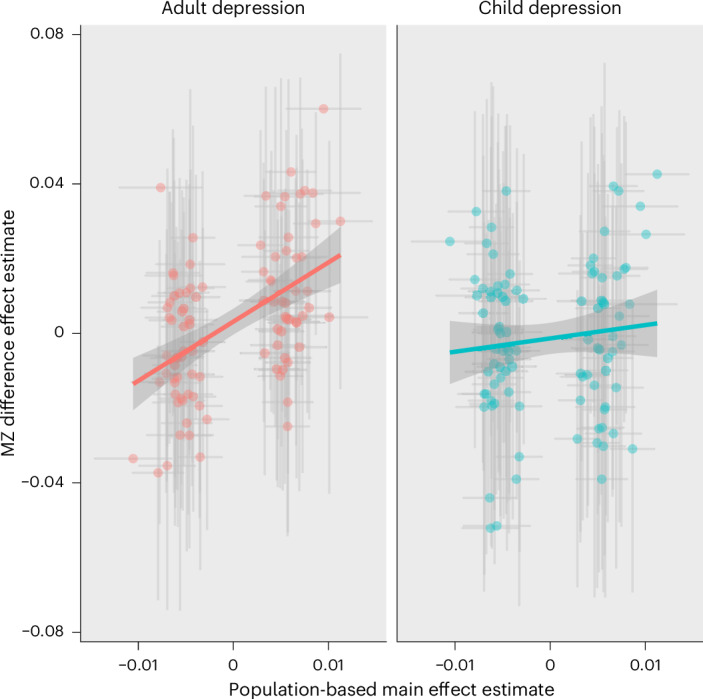
Two-sample Mendelian randomization estimates of the effect of genetic liability to depression on variance in depression. Genetic liability to depression, based on population studies is shown on the *x* axes and variance in depression scores (using 102 variants found to associate with depression incidence) is shown on the *y* axes. Genetic liability to depression increased the variability in depression outcomes and this was attenuated to the null in children (interaction term in linear regression *P* = 0.008). The error bars represent 95% confidence intervals of the variant effect estimates.

It has previously been speculated that environmental sensitivity might relate to polygenic liability rather than single loci, due to the environment interacting with a polygenic biological component^23^. We used Mendelian randomization to estimate the influence of the genetic liability of psychological phenotypes on their environmental sensitivity (Methods). We found a strong effect for depression (*β* = 0.84; s.e. = 0.26; *P* = 0.002). We also ran the analyses separately for our samples from children and those from adults (Fig. 4). The signal for depression was driven by analyses in adults (*β* = 1.58; s.e. = 0.29; *P* = 5 × 10^−7^), with the childhood association attenuated (*β* = 0.35; s.e. = 0.35; *P* = 0.36), and these estimates differed significantly (interaction *P* = 0.008). There was little evidence for heterogeneity of effect estimates across depression liability instruments. Other phenotypes did not exhibit an influence of liability on environmental sensitivity after correcting for multiple testing (Supplementary Table 14). We found little evidence that mean body mass indices and years of schooling affected phenotypic variance. Since vQTL effects could be biased by main effects, we conducted simulations to evaluate the sensitivity of the analytical approach to the Mendelian randomization results being driven by this bias (Supplementary Information section 7). We found that the MZ differences model can be liable to this problem under some phenotype normalization approaches, but that this was less likely to be driving the results in this study because we normalized by inverse rank transformation (Supplementary Fig. 17).

### Data simulation

**Fig. 5:**
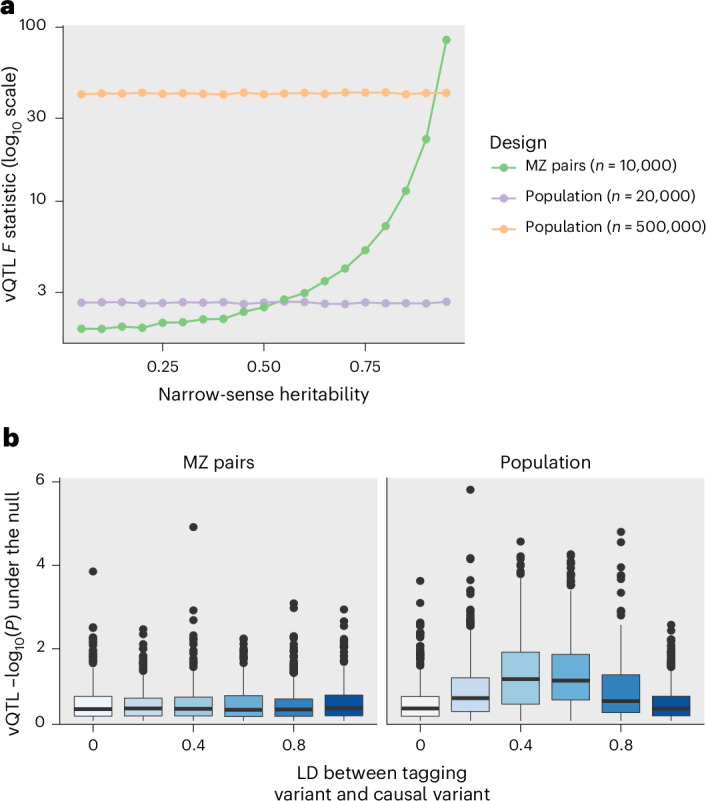
The MZ differences approach complements population-based vQTL detection. **a**, Comparison of the power between the MZ differences approach and the population-based approach (using the deviation regression model method) for detecting vQTLs. Comparisons were made between the MZ pairs approach and two different sample sizes for the population-based approach (one matching (with double the number of unrelated individuals; 20,000) and one with a typical sample size for modern GWASs (500,000)). The power of the MZ differences approach increases as the narrow-sense heritability increases. **b**, False discovery rates (*y* axis) due to incomplete linkage disequilibrium (LD) with a tagging causal variant (*x* axis), compared between the MZ differences approach (left) and population-based approach (right). The box plots represent interquartile ranges around median values of the simulations, with the whiskers representing ±1.5× the interquartile range and the points representing outliers. The presence of an additive causal variant tagging the tested SNP leads to elevated false discovery rates in the population approach, but not in the MZ differences approach.

We used simulations to investigate the properties of the method used to detect MZ twin-based variance loci and compared them with those of population-based vQTL methods. First, we found that the MZ twin-based method has the greatest power when the narrow-sense heritability is highest (Fig. 5a) and the residual variance is lowest, and therefore when variance effects explain the larger fraction of the difference between MZ pairs, as was suggested previously^12^. Second, we found that for moderate heritability the MZ differences approach has substantially greater statistical power than the population-based approach when sample sizes are equal (that is, 10,000 MZ twin pairs using the MZ twin-based method versus 20,000 unrelated individuals using the population-based vQTL method). However, in practice, the number of population-based samples generally available drastically outstrips the number of MZ twin samples available. When using a more realistic sample size (for example, 10,000 MZ twin pairs versus 500,000 population samples), the MZ differences approach only achieves similar power to the population approach when narrow-sense heritability values are very high (for example, >0.9). It has recently been shown that a variant tested for interaction can have inflated test statistics when in linkage disequilibrium with a strong additive effect^11^, and we investigated whether that mechanism can also adversely impact MZ twin-based estimates. Our simulations show that this problem is substantially exacerbated through population-based vQTL methods compared with direct interaction tests, but the MZ twin-based approach is robust to this bias (Fig. 5b).

## Discussion

Several genome-wide significant results were notable, including our finding that the *PTCH1* gene was associated with variation in depression symptoms, as this gene has previously been reported to be associated with depression-related phenotypes, including neuroticism^24,25^, anxiety^26^, depression symptoms^24^, feeling emotionally hurt^27^ and sensitivity to environmental stress and adversity^27^. The *C15orf38* gene (also known as *ARPIN-AP3S2*) was associated with variance in anxiety symptoms in our samples from children and has previously been associated with type 2 diabetes in adults^25,28^ and corticotropin-releasing factor protein levels^29^, which are involved in regulating anxiety, mood, eating and inflammation^30^. Hypoglycaemia symptoms in type 2 diabetes include a rapid heartbeat, sweating and nervousness, all of which are physical sensations associated with anxiety. It is possible that certain variants in this gene impact sensitivity to the effects of diet and stressors that are involved in the variability in insulin^31^, unpleasant physical sensations of which may be contextualized and made sense of as worries and anxieties (for example, the two-factor model of emotions^32^).

For autistic traits, the identified gene set included genes involved in tissue morphogenesis and healing that regulate the response to transforming growth factor beta (TGFβ1) levels and are involved in tissue repair pathways^33^. Growth factors serve important roles in neurodevelopment, immune function and development of the central nervous system and there is evidence that autism is associated with TGFβ1 and other growth factor-encoding genes^34–36^. For PLEs, the identified gene sets were related to the regulation of dopamine and catecholamine uptake. Our findings are supported by catecholamine’s involvement in the stress response and the hypothesized role of dopamine system dysregulation in the aetiology of psychosis^37^. Since gene–environment interactions have been implicated in variations in PLEs^38^, the association between the biological processes implicated herein and variability in psychotic experiences may partly be under the influence of the environment.

We also estimated the SNP heritability of environmental sensitivity (adolescent ADHD: *h*^2^ = 0.18 and s.e. = 0.11; child ADHD: *h*^2^ = 0.04 and s.e. = 0.06; adult autistic traits: *h*^2^ = 0.09 and s.e. = 0.15; depression: *h*^2^ = 0.03 and s.e. = 0.09), but the estimates were not statistically significant. We also showed that variants that affect mean levels of depression and anxiety influence the variance of these phenotypes. Several population-based methods for vQTL analysis are known to be susceptible to bias due to mean effects. In contrast, in principle, the MZ differences design is protected from this problem. Therefore, our study provides independent evidence that mean effects can influence variance.

The main strength of our study is the use of the MZ differences method to investigate the genetics of environmental sensitivity in the largest sample of MZ twins and a wide range of psychological phenotypes from a developmental perspective. The main limitation is the limited statistical power for detecting small genetic effects on variance. The findings should therefore be considered in light of our statistical power. Furthermore, as with all empirical analyses, our inferences depend on specific assumptions that may not hold. First, we took the MZ differences score to reflect genuine phenotypic variability. MZ differences, however, also reflect measurement errors that are difficult to separate from genuine phenotypic differences, and it is unclear whether MZ differences are stable across time. Future projects should investigate this approach with other phenotypes with low measurement errors, such as height, and consider a longitudinal design to assess the stability of these differences. Second, it is challenging to determine which mechanisms explain phenotypic variance. Genetic sensitivity to the environment and epigenetic processes, such as DNA methylation, imprinting, chorionicity and skewed X inactivation (for female MZ twins), also contribute to MZ phenotypic differences^15,39^. Also, the people included in our samples were all of European genetic ancestry since data from twins with DNA in other ancestries were not available in sufficient sample sizes. Our findings may not be generalizable to non-European genetic ancestries. The current study underlines the utility of DNA data from twins and should encourage funding for genetic data collection in multi-ancestry twin cohorts.

In summary, we have identified novel genetic factors associated with phenotypic variability. Our study illustrates the importance of large meta-analyses of genotyped MZ twin samples as a method for discovering and understanding the genetics of phenotypic variance and environmental sensitivity.

## Methods

This research complies with all of the ethical regulations related to the secondary analysis of data collected by various cohorts, each of which obtained ethical approval and informed consent.

### Ethical approval

#### Danish Twin Registry

The collection and use of biological material and survey information was approved by the Regional Committees on Health Research Ethics for Southern Denmark. This study is registered in Southern Denmrak University’s internal list (notification number 11.059) and complies with the rules of the General Data Protection Regulation.

#### Finnish Twin Registry

Ethics approval was obtained for multiple studies, the latest of which was related to the transfer of all available DNA samples, genotypes and associated phenotypes to the THL Biobank by the Hospital District of Helsinki and Uusimaa ethics board in 2018 (1799/2017).

#### Netherlands Twin Register

Ethical approval was provided by the Central Committee on Research Involving Human Subjects of the VU University Medical Center in Amsterdam, an institutional review board certified by the US Office for Human Research Protections (IRB number IRB-2991 under Federal-wide Assurance-3703; IRB/institute codes, NTR 03-180).

#### Murcia Twin Registry

The procedures of this registry have been approved by the University of Murcia Research Ethics Committee.

#### Older Australian Twins Study

The Older Australian Twins Study (OATS) was approved by the human research ethics committees of the Australian Twin Registry, University of New South Wales, University of Melbourne, Queensland Institute of Medical Research (QIMR) and South Eastern Sydney and Illawarra Area Health Service. Written informed consent was provided by all participants.

#### QIMR

Studies were approved by the QIMR Berghofer Medical Research Institute Human Research Ethics Committee.

#### Swedish Twin Registry

The different twin studies received separate approvals from the regional ethical review board in Stockholm (Dnr 80:80, 84:61; 93:226, 98:319 and 2010/657-31/3 (Swedish Adoption/Twin Study of Aging); Dnr 98:380 (OCTO-Twin); and Dnr: 97:051 and 2007/151-31/4 (HARMONY). Ethical approval for the PSYCH, TwinGene and Young Adult Twin Study in Sweden cohorts was given by the Uppsala Ethical Review Authority (2019-06066).

#### Twins Early Development Study (TEDS)

Ethical approval for TEDS has been provided by the King’s College London Ethics Committee (reference: PNM/09/10–104). Written informed consent was obtained prior to each wave of data collection from parents and from twins themselves from age 16 onwards.

#### TwinsUK

Approval was obtained from the TwinsUK BioBank, approved by the North West–Liverpool Central Research Ethics Committee (reference 19/NW/0187; Integrated Research Application System ID = 258513). This approval supersedes earlier approvals granted to TwinsUK by the St Thomas’ Hospital Research Ethics Committee and later by the London–Westminster Research Ethics Committee (reference EC04/015), which have now been subsumed within the TwinsUK BioBank.

### Study design

Figure  3 provides a flow chart for the current study. The eligibility criteria for inclusion of a study’s data in the present study analyses included: (1) cohorts comprising at least 100 pairs of MZ twins; (2) genotyping data for one or both twins; (3) availability of imputed genotype data (for example, 1000 Genomes or Haplotype Reference Consortium (HRC) data); (4) complete data from both twins for one or more phenotypes (imputation of missing data for incomplete pairs is not recommended); (5) complete covariate data for both twins (that is, age, sex and principal components for the genotyped twin); and (6) samples of European ancestry. Many of the authors of the studies with contributing data are part of the Within Family Consortium.

The analysis plan and pipeline were written, pre-specified and shared with interested cohorts to enable them to conduct GWASs of MZ differences for their available phenotypes, then the results were uploaded to a designated repository (https://github.com/LaurenceHowe/MZTwins-vQTL).

For some participating studies, data were available across the lifespan of participants, thus comprising repeated measures for certain phenotypes. It was therefore possible to explore genetic associations in the context of development by conducting developmentally stratified analyses. The developmental groups were defined as childhood (5–12 years of age), adolescence (13–18 years of age) and adulthood (>18 years of age). GWAS analyses were conducted separately for different cohorts and for each developmental stage if data were available. For anxiety symptoms, depression symptoms and autistic traits, GWAS data were available for children and adults. For ADHD symptoms, GWAS data were available for children and adolescents. For wellbeing and neuroticism, the samples comprised adults only, and for PLEs the samples comprised adolescents only.

Two sets of meta-analyses were then conducted using the GWAS results: a developmentally informed analysis, whereby GWAS results across studies were grouped according to the developmental stage of the sample and meta-analyses were conducted per phenotype (for example, depression child or depression adult); and a developmentally agnostic analysis, whereby a meta-analysis was conducted for each phenotype using the largest sample from each study, regardless of the developmental stage (for example, depression largest or anxiety largest). This ensured maximum power for meta-analysis per phenotype via the largest *n* value (Supplementary Information section 1.1 provides more details on study design).

### Samples

The samples analysed in this study included MZ twin pairs from cohort studies or twin registries in Australia and Europe (Supplementary Information Section 1.2 and Supplementary Table 1 provide details of the participating studies). Informed consent and ethical approvals were obtained for all participating cohorts (Supplementary Information). Table 1 shows the total sample sizes per phenotype and across developmental groups.

### Phenotypes

The MZ differences method requires continuous or categorical non-binary phenotypic data to calculate variance. Therefore, we used mean symptom scores instead of case/control diagnosis and the preference was for continuous measures. Various instruments have been developed for the assessment of psychological phenotypes, as was reflected in the participating studies. The scales differed in the numbers of items included, the types of symptoms assessed and the informant source. If multiple rating scales of a phenotype were available, study authors were asked to select the scale with the most items (tapping most symptom domains). Scales were coded so that higher values represented higher symptom levels. Absolute phenotypic differences were obtained for each MZ pair. Using linear regression, absolute phenotypic differences were corrected for age, sex, ten genetic principal components and any study-specific covariates. The residuals were standardized and rank transformed to be used as the phenotype in the GWASs. A brief description of each phenotype is provided below (Supplementary Information Section 1.1 gives details).

ADHD is a childhood-onset neurodevelopmental disorder of attention, activity and impulsivity. Symptoms commonly persist into adulthood. They are typically measured continuously using rating scales, often with separate scales for attention problems and hyperactivity or impulsivity, which can be summed to give a total score of ADHD symptoms.

Anxiety is heterogeneous, with clinical diagnoses comprising specific anxiety disorders (for example, phobias, post-traumatic stress disorder or social anxiety disorder) and generalized anxiety disorder. We were interested in generalized anxiety symptoms, usually measured via self-report, and reflected in a total score of anxiety symptoms.

Autism spectrum disorder is a neurodevelopmental disorder broadly reflecting difficulties in social interaction and verbal communication, as well as repetitive behaviours. Symptoms typically emerge in early childhood, and assessment is carried out via questionnaires and/or interviews. The continuous scores reflect the presence or extent of autism traits rather than a diagnosis of autism spectrum disorder.

Depression is heterogeneous, with many clinical presentations. A diagnosis requires a distinct change of mood, characterized by sadness or irritability and accompanied by psychophysiological changes, such as disturbances in sleep and appetite or loss of the ability to experience pleasure. The phenotypic scores for depression reflect the presence of any of these symptoms rather than a diagnosis of major depression.

Neuroticism is a personality domain and refers to a lack of emotional stability, stress vulnerability, the tendency to experience intense negative emotions, affects and cognitions, and impulsive behaviours under emotional strain. Neuroticism is considered a risk factor for anxiety and depression.

PLEs include a sub-clinical threshold of symptoms related to psychosis or schizophrenia disorders, such as persecutory ideation or perceptual abnormalities, that are prevalent in the community and non-clinical samples. PLEs are screened using self- or other-report questionnaires or interviews that cover some or all of these domains: paranoia, hallucinations, cognitive disorganization, anhedonia and negative symptoms.

Wellbeing includes both hedonic and eudaimonic wellbeing and is typically assessed via questionnaires that index an individual’s subjective sense of wellness, such as reporting satisfaction with one’s life or being hopeful and optimistic about it. The data from participating studies mainly related to subjective wellbeing (for example, life satisfaction). We preferred to use data for which wellbeing had been measured using a battery of questions. If data were only available from a single question reported on a Likert scale, the response variable was treated as a continuous scale.

### Genotypes

Participating studies were required to have genotype data from all 22 autosomes imputed to either the 1000 Genomes reference panel (preferably phase 3) or the HRC. Almost all contributing studies had already participated in a project related to the Within Family Consortium^10^ and used the same protocol in the automated scripts for genetic data preparation and quality control procedures before GWAS analysis. Minimum quality control requirements at the study level included: filtering SNPs for an imputation quality of >0.3 for HapMap imputed data or >0.5 for 1000 Genomes or HRC data; a call rate of >95%; and a minor allele frequency of >1%. Studies also removed one pair randomly when there were two MZ pairs with a kinship of >0.1. Study-level genotyping and quality control information are included in the Supplementary Data.

### Analyses

#### Simulations

We investigated the statistical properties of the MZ differences GWAS method using simulations. Methods using family-based design, such as the MZ differences method, complement population-based vQTL methods in three ways: (1) statistical power; (2) robustness to bias due to additive effects; and (3) providing an alternative identification strategy for triangulation^40^. To simulate vQTLs, we used the following data-generating model.

$${y}_{i,t}\,=\alpha +{\beta }_{1}{G}_{i}+{z}_{i}+{v}_{i,t}+{e}_{i,t}$$

where *y*_*i,t*_ is the phenotypic value for twin *t* = {A,B} at MZ pair *i* = {*1*,…,*n*} and *α* is an intercept term, which is set to 0 for simplicity. *β*_1_ is the additive effect of the SNP *G*_*i*_, which is distributed as

$${G}_{i}\sim{{\rm{Binom}}}\left(2,p\right)$$

where *p* is the allele frequency. Note that *G*_*i*_ is the same for both twins in the *i*th MZ pair. *z*_*i*_ is the remaining polygenic risk for the MZ pair, which is normally distributed with a mean of 0 for simplicity, and variance is defined as

$${z}_{i}\,\sim \,n\left(0,{{\sigma }^{2}}_{{\rm{g}}}-2p\left(1-p\right){{\beta }_{1}}^{2}\right)$$

where ${{\sigma }^{2}}_{{\rm{g}}}$ is the genetic variance of the trait. The variance heterogeneity term *v*_*i,t*_ is modelled as

$${v}_{i,t}\sim n(0,{\beta }_{2}{G}_{i})$$

such that each additional allele at *G* increases the within-MZ difference by a factor of *β*_2_. Finally, *e*_*i,t*_ is the independent individual residual variance defined as

$${e}_{i,t}\sim n\left(0,1-{{\sigma }^{2}}_{{\rm{g}}}-{{\sigma }^{2}}_{{\rm{v}}}\right)$$

where ${\sigma }_{{\rm{v}}}^{2}$ is the variance of *v*_*i,t*_. Therefore, for a pair (A and B) of MZs, the additive genetic factors *G*_*i*_ and *z*_*i*_ are fixed, but the within-MZ variability is induced from the *v*_*i,t*_ + *e*_*i,t*_ terms. We estimated vQTL effects from unrelated individuals (choosing one MZ at random) using the deviation regression model from Marderstein and colleagues^41^. We estimated vQTL effects using MZs and the following MZ difference model:

$$\,\left|{y}_{i,{\rm{A}}}-{y}_{i,{\rm{B}}}\right|={\hat{\beta }}_{2}{G}_{i}+{\epsilon }_{i}$$

where ${\hat{\beta }}_{2}$ is an estimate of the vQTL effect, and *ϵ*_*i*_ is the residual error from this regression. We investigated the power of each method by generating vQTL effects (*β*_2_) calibrated to have 80% statistical power for the deviation regression model method in 500,000 unrelated individuals (with parameters *p* = 0.3 and *β*_1_ = 0). We then estimated how the power of the MZ differences approach varies for these parameters across a range of narrow-sense heritability values ${{\sigma }^{2}}_{{\rm{g}}}={h}^{2}$. We calculated the power for detecting a vQTL at the genome-wide significance level by drawing 1,000 replications and identifying the fraction of simulations that had *P* < 5 × 10^−8^.

We estimated the false discovery rate for vQTL in the presence of tagging additive loci, following the approach outlined by Hemani and colleagues^11^. Briefly, the data-generating model described above is simulated with an additive effect of *β*_1_ = 0.1 generated, but the vQTL test is performed at a second locus, G^*^, that is generated to be in linkage disequilibrium with *G*. The simulations were then performed with no vQTL effect (*β*_1_ = 0), variance of the linkage disequilibrium between *G* and G^*^ (*r*^2^ = 0,…,1), *h*^2^ = 0.5 and *p* = 0.1, and 500 repeats were performed for each parameter combination.

#### GWAS model

In our primary analysis, we estimated the association between the absolute phenotypic difference between MZ twins (residualized for age, sex and principal components and then standardized and rank transformed) and the genetic marker using linear regression for each SNP, *j*:

$$|{y}_{i,{\rm{A}}}-{y}_{i,{\rm{B}}}| \sim \,{\beta }_{2,\;j}{G}_{{ij}}+{\epsilon }_{i,\;j}.$$

We constructed two further models for sensitivity analyses: model 2, for which the within-twin mean of the phenotype was a covariate in the regression; and model 3, which differed from our primary model by not adjusting for principal components when constructing the phenotype. Model 2 was constructed to examine whether adjusting for the within-twin mean in the GWAS model would significantly impact the SNP associations, which would be the case if the MZ differences largely reflected mean differences. However, this also risks over-correcting, especially for vQTLs, which affect both the mean and variance of a phenotype, as was indicated here by a small-to-moderate (*r* = ~0.3–0.6) positive correlation between the MZ phenotypic mean and MZ phenotypic differences in our sample (as was previously proposed^42^). Model 3 was constructed since some participating studies were likely to be very small (fewer than 300 participants). Including ten principal components for all studies might have been overly conservative, leading to an inflation of *P* values.

We used the sign test to assess whether model 2 and 3 results were similar to those of model 1, as indicated by the correlation between *β* values, *P* values and the direction of the effect. The results indicated that models 2 and 3 were not significantly different from model 1 (Supplementary Table 3a–c). Therefore, we considered model 1 to be the most parsimonious, with a lower number of parameters than model 2 but similar results, while also correcting for population stratification confounding. The remaining analyses were therefore conducted using model 1 results only.

#### Quality control procedure

Study-level GWAS results (Supplementary Figs. 2–7) were quality controlled using EasyQC (version 23.8)^19^. Variants with missing estimate (*β)* values, standard error (s.e.) values, statistical significance (*P*) values or imputation quality score and SNPs with a minor allele frequency of <0.01 or an imputation quality score of <0.5 were removed. Cptid identifiers (chr:bp:A1:A2) were created and alleles, effects and frequencies were checked in all GWAS results and harmonized according to their respective reference panel (1000 Genomes phase 3 version 5 or the HRC). SNPs with mismatching alleles compared with the reference panel were removed. Indels, monomorphic SNPs and duplicate SNPs that could also be tri-allelic (with the same base pair position but different alleles) were removed, retaining only the SNP with the largest sample. Manhattan and QQ plots were obtained and lambda-median values were inspected for *P* value inflation (Supplementary Figs. 2–6 and Supplementary Table 2 provide more details).

#### GWAS meta-analysis

METAL (2011 release)^20^ was used to conduct inverse-variance-weighted fixed-effect meta-analysis across studies, per phenotype. First, a meta-analysis was conducted by selecting the GWAS result with the largest sample from each study, regardless of the developmental stage. Another set of GWAS meta-analyses were then conducted in developmentally stratified samples for depression (child and adult), anxiety (child and adult), ADHD (child and adolescent) and autistic traits (child and adult) phenotypes (Supplementary Table 4). Cptid IDs were mapped into rsIDs from the 1000 Genomes phase 3 version 5 European panel. The SNP2GENE function in the FUMA web application (version 1.5.0)^21^ was used to annotate GWAS SNPs and identify independent significant SNPs (SNPs in linkage disequilibrium with the lead SNP at *r*^2^ = 0.1; lead SNPs are those in linkage disequilibrium with any independent significant SNPs with *r*^2^ > 0.6; Supplementary Table 5) and to produce regional, QQ and Manhattan plots (Supplementary Figs. 7–11).

#### Gene-based and gene set analyses

MAGMA (version 1.08) in the FUMA web application (version 1.5.2)^21^ was used to annotate GWAS SNPs and conduct gene-based and gene set analyses. Meta-analysed GWAS results were filtered to include only SNPs available in at least 50% of studies. SNPs were annotated to Ensembl version 92 protein-coding genes for gene-based analyses using default parameters (a SNP-wise model for gene testing). A competitive test was conducted for gene set analyses using default gene sets in FUMA from MsigDB version 7.0, totalling 15,496 gene sets (5,500 curated gene sets and 9,996 Gene Ontology terms). Curated gene sets contained nine data resources, including the Kyoto Encyclopedia of Genes and Genomes, Reactome and BioCarta. Gene Ontology terms comprised three categories: biological processes, cellular components and molecular functions. The major histocompatibility complex region was excluded from all annotations (Supplementary Table 5 provides details).

#### Heritability analysis

SNP heritability estimates per phenotype were obtained using LDSC (version 1.0.1)^22^. The European 1000 Genomes linkage disequilibrium scores generated by the authors of LDSC were used, and SNPs for heritability analyses were merged with a set of ~1.2 million high-quality SNPs defined by the authors of LDSC^22^.

#### Developmentally stratified analyses

For ADHD symptoms, data were available for children (5–12 years of age) and adolescents (13–18 years of age), whereas for anxiety, depression and autistic traits, data were available for both children and adults (>18 years of age). The stratified analyses included a meta-analysis of GWAS results separately for samples from children, adolescents and adult for these phenotypes, followed by gene-based, gene set and heritability analyses, using the same criteria as the largest non-stratified sample.

#### Mendelian randomization analysis

We used a two-sample summary data Mendelian randomization to estimate the influence of the genetic liability of psychological phenotypes on their environmental sensitivity. Mendelian randomization uses genetic variants as instrumental variables for the exposure of interest. Three assumptions define instrumental variables: (1) relevance (the instrument must be associated with the exposure); (2) independence (there must be no uncontrolled confounders of the instrument–outcome association); and (3) exclusion restriction (the instruments only affect the outcome via the exposure of interest). We used the summary data from the MZ twins GWAS as an outcome in our Mendelian randomization analyses and investigated whether differences in the mean of each exposure (for example, depression) affect the variance in each psychiatric outcome. The exclusion restriction assumption requires that SNPs only affect the variance in the outcome via their mean effects on the exposure (liability to depression). This assumption would be violated if these variants also affected the outcome via their effects on the variance in depression, independent of their effects on the mean liability to depression. Under such a scenario, our estimates may be biased. Theoretically, this bias could be in either direction. More methodological research would be useful to determine the likelihood and magnitude of these potential biases. We selected 102 independent (linkage disequilibrium = 10,000 kilobases; *r*^2^ = 0.001) genetic variants associated with depression in Howard et al.^43^ as instruments for genetic liability to depression. We harmonized the effects by effect allele, chromosome and position on build hg19. We used the inverse-variance-weighted estimator to estimate the effect of genetic liability to depression on phenotypic variability using the TwoSampleMR package^44^. The variants were strongly associated with depression and there was minimal overlap between our samples and those used in the GWASs to select variants. Therefore, weak instrument bias is unlikely^45^. We followed the same procedure for the other phenotypes, but with a different number of SNPs (Supplementary Table 14). Because depression is strongly genetically correlated with anxiety but there are no well-powered GWASs for anxiety, we performed a similar analysis for variance in anxiety but using the 102 variants for depression. Here the main effects for anxiety at each of the 102 variants were obtained from a GWAS using UK Biobank data on self-reported anxiety measures^46^. Finally, we also tested educational attainment^47^ and body mass index. GWAS summary statistics for main effects were obtained from OpenGWAS^48^. We reported this analysis using the STROBE-MR checklist^49^.

### Reporting summary

Further information on research design is available in the Nature Portfolio Reporting Summary linked to this article.

## Supplementary information

Supplementary Information Supplementary Tables 1–14 and Figs. 1–18, Study design and cohort-level information, Quality control details and Results.

Reporting Summary

Peer Review File

Supplementary Data Study-level information on the phenotypes used in the meta-analyses and genotyping information for each cohort.

## Data Availability

Meta-analysed GWAS summary statistics from the current study are publicly available from OpenGWAS (https://gwas.mrcieu.ac.uk/). Accession codes for the GWAS meta-analyses are as follows: ieu-b-5146 (adolescent) and ieu-b-5147 (child) for MZ twin differences in ADHD symptoms; ieu-b-5148 (largest), ieu-b-5149 (adult) and ieu-b-5150 (child) for MZ twin differences in anxiety symptoms; ieu-b-5151 (largest), ieu-b-5152 (adult) and ieu-b-5153 (child) for MZ twin differences in autistic traits; ieu-b-5154 (largest), ieu-b-5155 (adult) and ieu-b-5156 (child) for MZ twin differences in depression symptoms; ieu-b-5157 for MZ twin differences in neuroticism score; ieu-b-5158 for MZ twin differences in PLEs; and ieu-b-5159 for MZ twin differences in subjective wellbeing. Data from individual studies are not publicly available and are subject to strict access control because the consent given by the participants does not allow for data storage on an individual level in repositories or journals. Access to these data requires specific approval from the relevant data access committees for each cohort. Mapping and allele frequency reference files (all based on National Center for Biotechnology Information build 37) for 1000 Genomes phase 1 version 3, 1000 Genomes phase 3 version 5 and the HRC are available via https://www.uni-regensburg.de/medizin/epidemiologie-praeventivmedizin/genetische-epidemiologie/software/index.html.

## Code availability

Scripts for GWAS analyses are available from GitHub at https://github.com/Elham-Assary/MZ-differences-GWAS.
