# Supplementary material for: Genetics of monozygotic twins reveals the impact of environmental sensitivity on psychiatric and neurodevelopmental phenotypes
Source: Nat Hum Behav. 2025 Jun 10;9(8):1683–96. doi: 10.1038/s41562-025-02193-7 (PMC12367547; doi:10.1038/s41562-025-02193-7)
Supplement: Supplementary file 1 — Supplementary Tables 1–14 and Figs. 1–18, Study design and cohort-level information, Quality control details and Results. [file 41562_2025_2193_MOESM1_ESM.pdf]

nature human behaviour

Supplementary information

https://doi.org/10.1038/s41562-025-02193-7

Genetics of monozygotic twins reveals the impact of environmental sensitivity on psychiatric and neurodevelopmental phenotypes

In the format provided by the authors and unedited

Supplementary information

Table of Contents

1. STUDY DESIGN AND PARTICIPATING COHORTS....2

1.1. STUDY DESIGN....2
1.2. PARTICIPATING STUDIES AND MEASURES....4
Table S1. Participating studies N per phenotype....20

2. STUDY-LEVEL GWAS OF MZ DIFFERENCES AND QC....21

2.1. GWAS MODEL....21
2.2. STUDY-LEVEL GWAS QC....22
Table S2. Study Level information and QC....23
Figures S2 – S6: Study-level QQ-Plots of GWAS of MZ differences....24

3. GWAS META-ANALYSES....29

Tables S3a – S3c: GWAS model comparisons....30
Table S4. Top SNPs per phenotype....33
Figures S7-S11: Manhattan and QQ plots of MZ GWAS meta-analyses....34

4. GENE-BASED AND GENE-SET ANALYSES....39

Table S5. MAGMA parameters and descriptions....40
4.1. MAGMA GENE-BASED ANALYSES RESULTS....41
Figures S12 -S17: gene-based Manhattan and QQ plots....41
Table S6. Top genes per phenotype and across developmental groups....46
4.2. MAGMA GENE-SET ANALYSES RESULTS....47
Table S7. Top gene-sets per phenotype and across developmental groups....48
Tables S8 – S11: Genome-wide significant gene-sets details....49
Table S12: Genome-wide significant results per phenotype....52

5. HERITABILITY ANALYSES....53

Table S13. SNP-heritability estimates per phenotype....53

6. MR ANALYSIS....54

Table S14: Influence of disease liability on environmental sensitivity.....54

7. EVALUATION OF VARIOUS DATA GENERATING MODELS FOR MZ DIFFERENCES....55

Figure S17: Evaluation of the MZ difference model for different genetic mechanisms....56

8. FUNDING, ACKNOWLEDGEMENTS, AND ETHICAL APPROVALS....57

9. REFERENCES....60

1. Study design and participating cohorts

1.1. Study Design

We aimed to use MZ differences design to a) estimate genetic associations with phenotypic variability, b) estimate the SNP-heritability of phenotypic variability, and c) estimate genetic associations with phenotypic variability stratified by developmental stage. We investigated seven phenotypes: ADHD symptoms, ASD symptoms, anxiety symptoms, depression symptoms, psychotic-like experiences (PLE), neuroticism and wellbeing.

This involved using genome-wide genetic data and phenotypic data from MZ twin pairs from Australia and Europe. Twin cohorts that were part of the *within-family consortium*, as well as additional cohorts were invited to take part in the study. The minimum requirements for participating were having at least 100 pairs of MZ twins satisfying ALL of the following criteria:

i. one or both twins must be genotyped.
ii. imputed genotype data is available (e.g. 1000 Genomes or HRC).
iii. both twins must have complete data for one or more phenotypes. Imputation of missing data for incomplete pairs are not recommended.
iv. both twins must have complete covariate data (age, sex, and principal components for the genotyped twin).
v. samples are of European ancestry.

The analysis plan was shared with interested cohorts and studies were asked to conduct GWAS of MZ differences on their available phenotypes using automated scripts to ensure consistency across studies (https://github.com/LaurenceHowe/MZTwins-vQTL) and upload the results in a designated repository.

Some of the participating studies were longitudinal in design, whereby data were collected across lifespan. To explore genetic associations in the context of development, we did the following:

- Defined developmental groups as childhood (5-12 years old), adolescence (13-18 years old), and adulthood (>18 years old).
- Participating studies were asked to run GWAS analysis separately in each developmental sample, if their study included repeated measures across life span (e.g., ages 9, 15, 30 at the time of data collection)
- If they had repeated measures that was collected during the same life stage (e.g., data collected at age 25 and 55), they were asked to use the sample with the largest number of participants.
- Studies were advised to restrict the data to the same waves of data collection, rather than aggregating the data that was obtained from different waves of data collection with large intervals (e.g. twin pair 1 from wave 1 and twin pair 2 from wave 5). This was to ensure consistency in the sample, within each study, since many measures define a time-line within which symptoms are explored (e.g., in the past 6 months, in the last year).

Two sets of GWAS meta-analysis were conducted on the GWAS results for each phenotype:

- developmentally agnostic meta-analysis (the largest sample): selecting the largest-sampled GWAS from each cohort for meta-analysis, regardless of the developmental stage of the sample. This ensured maximum power for meta-analysis per phenotype, by way of securing the largest N.

- Developmentally informed meta-analysis (developmental samples): GWAS results across cohorts were grouped according to the developmental stage of the sample for each phenotype, and separate meta-analyses were conducted for each developmental stage (e.g., depression-child, depression-adult). For these meta-analyses, we set a minimum total sample size of 5,000 across studies for each phenotype, to ensure enough power as recommended for heritability analyses [1].

Phenotypes: the phenotypes of interest were symptoms of ADHD, anxiety, autism spectrum disorder, depression, as well as psychotic-like experiences, neuroticism and wellbeing. The main requirement was that data for phenotypes were continuous or categorical non-binary. Continuous phenotypes were preferable. A variety of rating scales have been developed to assess psychological phenotypes, and this was reflected in the participating studies. The scales differed in the number of items included and with respect to the types of symptoms assessed. If multiple rating scales of a phenotype were available, studies were asked to select the scale with most items (tapping most symptom domains). Details of measures used in participating studies are presented in section 1.1. Study-level descriptives for all phenotypes are presented in supplementary information file: **Phenotype_AllStudies.xlsx.**

Genotypes: Studies were required to have genotype data from all 22 autosomes imputed to either the 1000 genomes reference panel (preferably phase 3) or Haplotype Reference Consortium (HRC). Many of the studies had already participated in a related project from this consortium [2], and were advised to use the same script for genetic data preparation and quality control procedures. Minimum quality control requirements at the study level included filtering SNPs for imputation quality > 0.3 for HapMap imputed data and > 0.5 for 1000G or HRC data, call rate > 95%, and minor allele frequency (MAF) > 1%. Studies also removed one pair at random when there were two MZ pairs with kinship > 0.1. Study-level genotyping and QC information are in supplementary materials: **Gentoyping_AllStudies.xls.**

1.2. Participating studies and measures

A total of 9 twin cohorts participated in the current study, some providing data from multiple studies within each cohort. Details of participating studies and the measures used in each study to index phenotypes are below. **Table S1** shows sample sizes from participating studies for each phenotype and details of participating cohorts**.** Study-level descriptives for all phenotypes are presented in supplementary information: **Phenotype_AllStudies.xls**

Danish Twin Registry (DTR)

This study included monozygotic twin pairs recruited by the Danish Twin Registry (DTR) as part of the study of Middle-Aged Danish Twins (MADT) and the Longitudinal Study of Aging Danish Twins (LSADT) (Pedersen et al, 2019). Briefly, MADT was initiated in 1998 and includes 4,314 twins randomly chosen from the birth years 1931-1952. Surviving participants were revisited from 2008 to 2011, where the blood samples used in this study were collected. LSADT was initiated in 1995 and includes twins aged 70 years and older. Follow-up assessments were conducted every second year through 2005. The individuals included in the present study all participated in the 1997 assessment, where blood samples were collected from same sex twin pairs. To minimize the within-twin-pair difference in age at phenotyping as much as possible, the phenotype data used for MADT study participants were all collected as part of the 1998 assessment, although data for several of the phenotypes were also available from the follow-up assessment. All data for the LSADT study participants were collected as part of the 1997 assessment.

References

Pedersen DA, Larsen LA, Nygaard M, Mengel-From J, McGue M, Dalgård C, Skytthe A, Kyvik KO, Hvidberg L, Hjelmborg JvB, Holm NV, Christensen K. The Danish Twin Registry: An Updated Overview. Twin Research and Human Genetics, 1-9. DOI: 10.1017/thg.2019.72.

Phenotypes

Depressive symptoms

Depressive symptoms were assessed using a 17-item scale adapted from the Depression Section of the Cambridge Mental Disorders of the Elderly Examination (CAMDEX), evaluating both somatic and affective symptoms (McGue & Christensen, 1997). The depressive symptoms score was calculated as the sum of the 17 items, with a higher score corresponding to more severe symptoms.

Subjective wellbeing

This was evaluated using the question 'Are you happy and satisfied with your life at present?', with the response options 1 = Yes, always, 2 = Yes, nearly always, 3 = Yes, now and then, 4 = No, hardly ever, and 5 = No, never. Prior to analysis, the variable was recoded so that higher values represented higher levels of subjective wellbeing.

References

McGue M, Christensen K. Genetic and environmental contributions to depression symptomatology: Evidence from Danish Twins 75 years of age and older. J. Abnorm. Psychol. 106(3), Aug 1997, 439-448.

Finish Twin Cohort (FinnTwins)

Finnish Twin Cohort consists of three birth cohorts of twin born in Finland: 1) same-sex twin pairs born before 1958 and with both cotwins alive in 1974. They have been followed up since 1975, with cohort-wide questionnaires in 1975, 1981, 1990 and 2011 (Kaprio et al 2019). 2) twins born 1975-1979, follow-up from their 16th birthday onwards with surveys at ages 16, 17 and 18.5 yrs, and in their mid-twenties and mid-thirties (Kaidesoja et al, 2019) 3) Twins born 1983-1987, follow-up from their 11th birthday onwards with surveys at ages 11/12, 14, 17 and 18.5 yrs, and in their mid-twenties and mid-thirties (Rose et al, 2019). The data for the current study came from different studies within the registry.

FT12: FinnTwin12 study having the target population of all Finnish twins born in 1983–1987 ($N = 6272$). From the Finnish population registry (which covers the entire population), the twins were identified as those born on the same day to the same mother. The baseline survey was conducted in the year the twins turned 11, with follow-up questionnaires at ages 14, 17 and as young adults. Part of the twin families were invited to interviews and in-person assessments at ages 14 and as young adults, when DNA was extracted from blood samples and saliva for genotyping.

FT16: FinnTwin16 (FT16) cohort including Finnish twins born in 1975–1979 and identified from the population register ($n = \sim5600$ twin individuals). The FT16 study was targeted to investigate determinants of health-related behaviours, disease risk factors, and chronic diseases in adolescents and young adults. The baseline survey was at age 16, with follow-up surveys at ages 17, 18.5, as young adults and in their thirties. DNA was obtained from pairs participating in targeted clinical subsample studies.

Old Twin Cohort: The older part of the Finnish Twin Cohort consists of all Finnish twin pairs of the same gender born before 1958 with both co-twins alive in 1975. These twin pairs were selected from the Central Population Registry of Finland in 1974. Four surveys of the entire cohort have been carried out. The first questionnaire was mailed to all pairs in August-October 1975. Three follow-up questionnaire studies have been carried out in 1981, 1990, and 2011. The two latter surveys were mailed to twins born 1930-1957 and 1945- 1957 respectively. In addition, twins born prior to 1945 have been invited to a dementia screening interview between 2000 and 2017.

References

Rose RJ, Salvatore JE, Aaltonen S, Barr PB, Bogl LH, Byers HA, Heikkilä K, Korhonen T, Latvala A, Palviainen T, Ranjit A, Whipp AM, Pulkkinen L, Dick DM, Kaprio J. FinnTwin12 Cohort: An Updated Review. Twin Res Hum Genet. 2019;22(5):302-311. doi: 10.1017/thg.2019.83. . PMID: 31640839;

Kaidesoja M, Aaltonen S, Bogl LH, Heikkilä K, Kaartinen S, Kujala UM, Kärkkäinen U, Masip G, Mustelin L, Palviainen T, Pietiläinen KH, Rottensteiner M, Sipilä PN, Rose RJ, Keski-Rahkonen A, Kaprio J. FinnTwin16: A Longitudinal Study from Age 16 of a Population-Based Finnish Twin Cohort. Twin Res Hum Genet. 2019 ;22(6):530-539. doi: 10.1017/thg.2019.106. PMID: 31796134.

Kaprio J, Bollepalli S, Buchwald J, Iso-Markku P, Korhonen T, Kovanen V, Kujala U, Laakkonen EK, Latvala A, Leskinen T, Lindgren N, Ollikainen M, Piirtola M, Rantanen T, Rinne J, Rose RJ, Sillanpää E, Silventoinen K, Sipilä S, Viljanen A, Vuoksimaa E, Waller K. The Older Finnish Twin Cohort - 45 Years of Follow-up. Twin Res Hum Genet. 2019;22(4):240-254. doi: 10.1017/thg.2019.54.. PMID: 31462340.

Phenotypes

ADHD symptoms

These were measured in the FinnTwin12 cohort with Multidimensional Peer Nomination Inventory (MPNI; Pulkkinen, Kaprio & Rose, 1999), a 30-item inventory devised specifically to measure, among other behaviours, ADHD symptoms based on DSM diagnostic criteria. X items were used to ask for attention and hyperactivity. Items for Hyperactivity-Impulsivity include (‘Is restless’, ‘Does not wait for his turn’), while inattention was for example “has poor concentration”. Higher scores represent higher levels of ADHD. The MPNI was used to rate the twins' behaviour by teachers (at ages 12 & 14), parents (at age 12) and by the twins themselves (at ages 14 & 17) as well as cotwin (sibling) ratings (at ages 14 & 17). We used two ratings, parent at age 12 and teacher at age 14 as two separate variables in the present analysis. The teacher ratings has high Cronbach’s alphas (over 0.9 for the attention and hyperactivity in boys and girls), while parent ratings were lower (0.6 to 0.8 range, see Pulkkinen et al, 1999).

Depression symptoms

were measured with Beck Depression Inventory (Beck, Ward, Mendelson, Mock, Erbaugh, 1961), in the older twin cohort. This is a 21-item self-report inventory devised specifically to measure depression symptoms. Higher scores represent higher levels of depressive symptoms. Cronbach’s alpha of the measure is 0.85.

Neuroticism

were measured with Eysenck Personality Inventory- Brief (Viken, Rose, Kaprio, Koskenvuo,1992), a 19-item self-report inventory devised specifically to measure neuroticism (10 items) and extraversion (9 items). Higher scores represent higher levels of neuroticism. Neuroticism was administered in the older twin cohort in 1975, 1981 and 2011. We used the combined data from the 1981 & 1975 questionnaires in the present analysis (i.e., mean of 1975 and 1981 when both were available, or the single response when only one questionnaire of the two had been answered). Two sample items are:
“*Are you often uneasy, feeling that there is something you want without knowing what it is? Do you think you usually worry too long after a distressing situation?* “
The respondent is asked to endorse the response (yes or no) that best describes how they feel and act. Cronbach’s alpha of the measure is 0.73.

Wellbeing

Wellbeing was measured with questionnaire items on happiness from multiple questionnaires. These were one item from the Five Factor Inventory in FinnTwin12 as young adults, two questionnaires in adulthood in FinnTwin16 and from the 1975, 1981, 1990 and 2011 questionnaires. The wordings slightly differ but basically are similar and scored from 1

to 5 with cannot say/ do not know scored as "3". The FinnTwin16 and Older cohort item is one item from a four-item scale on Life Satisfaction. The higher sum score represents higher levels of wellbeing.
Do you feel that your life at the moment is:
[1] cannot say; [2] very sad; [3] fairly sad; [4] fairly happy; [5] very happy
I am happy and cheerful:
[1] I fully disagree; [2] I disagree; [3] Do not know ; [4] I agree ; [5] I fully agree

References

Beck, A. T., Ward, C. H., Mendelson, M., Mock, J., & Erbaugh, J. (1961). An inventory for measuring depression. *Arch Gen Psychiatry, 4*, 561-571.

Koivumaa-Honkanen H, Honkanen R, Koskenvuo M, Kaprio J. Self-reported happiness in life and suicide in ensuing 20 years. Soc Psychiatry Psychiatr Epidemiol. 2003 May;38(5):244-8. doi: 10.1007/s00127-003-0625-4. PMID: 12719839.

Pulkkinen, L., Kaprio, J., & Rose, R. J. (1999). Peers, teachers and parents as assessors of the behavioural and emotional problems of twins and their adjustment: the Multidimensional Peer Nomination Inventory. *Twin Research and Human Genetics*, *2*(4), 274-285.

Viken, R. J., Rose, R. J., Kaprio, J., & Koskenvuo, M. (1994). A developmental genetic analysis of adult personality: extraversion and neuroticism from 18 to 59 years of age. *Journal of personality and social psychology*, *66*(4), 722.

Murcia Twin Registry (MTR)

MTR is a population-based register of adult twins in the region of Murcia, located in southeast Spain. To date, the MTR has identified over 3,500 participants born between 1940 and 1976. Information is obtained longitudinally by face to face or telephone administered interviews and includes data on demographics, health, lifestyle and related variables. In addition, biological samples (DNA) have been collected in a subset of the sample. The registry procedures have been approved by the University of Murcia Research Ethics Committee. For more details, see Ordoňana et al. (2019).

References

Ordoñana, J. R., Carrillo, E., Colodro-Conde, L., García-Palomo, F. J., González-Javier, F., Madrid-Valero, J. J., Martínez Selva, J. M., Monteagudo, O., Morosoli, J. J., Pérez-Riquelme, F., & Sánchez-Romera, J. F. (2019). An Update of Twin Research in Spain: The Murcia Twin Registry. *Twin research and human genetics*, *22*(6), 667–671. https://doi.org/10.1017/thg.2019.60

Phenotypes

Wellbeing
This was assessed with the Visual Analogue Scale (VAS) measure of the EQ-5D questionnaire (Szende & Williams, 2004). This scale records the respondent's overall self-rated health-related quality of life on a graduated (0-100) scale, with higher scores for higher quality of life. The endpoints of the scale are labelled as 'the best' and 'the worst' the respondent can imagine.

Depressive symptoms
Symptoms were measured with the Spanish version of the State-Trait Depression Inventory (Spielberger et al., 2005), a 20-item self-report inventory devised specifically to measure depression. The questionnaire is composed of two subscales, namely *state depression* and *trait depression*, each of them composed of 10 items with 4-point scale answer options (1-4), with the final score ranging 10-40 for any of them. The *state* subscale items refer to how the participants feel 'at the moment', whereas the *trait* items refer to how participants 'generally' feel. Data used for this GWAS came from the *trait depression* subscale. Cronbach alpha was 0.88. Higher scores represent higher levels of depressive symptoms.

References
Spielberger et al. Analysis of convergent and discriminant validity of the Spanish experimental version of the State-Trait Depression Questionnaire (ST-DEP). Actas Esp Psiquiatr 2005;33:374–82.
Szende A, Williams A. Measuring self-reported population health: an international perspective based on EQ-5D: Netherlands: SpringMedpublishing, 2004

Netherlands Twin Registry (NTR)

NTR is a population-based longitudinal cohort study that collects data in twins, multiples, and their family members. NTR participants are recruited through birth felicitation services, city councils, and online platforms. Every couple of years, biological and non-biological family members are invited to partake in surveys that contain questions about development, health, behaviour, and lifestyle. For the current study, child data came from the Young NTR (YNTR). Both parents are asked to fill out a survey (on a broad set of behaviour and health related traits) on the twins at ages 2,3,5,7,9/10 and 12. The adolescent data were also obtained from the YNTR, but from a subsample of adolescents that filled out the Dutch Health and Behavior Questionnaire (DHBQ) that was send to adolescents at age 14,16, and 18.
Adult data were retrieved from the Adult NTR (ANTR). Self-report surveys are sent around every 2-3 years to ANTR participants.

References
Bartels, M., Boomsma, D. I., Bruins, S., Davies, G. E., Dolan, C. V., Ehli, E. A., . . . van't Ent, D. (2019). The Netherlands Twin Register: Longitudinal Research Based on Twin and Twin-Family Designs. *Twin Research and Human Genetics, 22*(6), 623-636. doi:10.1017/thg.2019.93

Phenotypes

ADHD symptoms
Child sample: symptoms were measured with the Conners' Parent Rating Scales Revised Short-Form (CPRS-R-S) ADHD index subscale, a 12-item mother-report subscale to measure ADHD symptoms (Kollins, Epstein & Conners, 2014). The parents are asked to rate items based on the youth's behaviour over the past month on a scale from 0 to 3, where 0 is not true at all, and 3 is very much true. The items are summed to create a ADHD sum-score, with higher scores indicating higher levels of ADHD.
Adult sample: symptoms were measured using Conners' Adult ADHD rating scales self-report screening version (CAARS-S:SV) (Conners, Erhardt & Sparrow, 1999). The scale measures ADHD symptoms according to DSM-IV criteria. Individuals are asked to rate themselves on 30 items using a 4-point scale where 0 indicates not at all or never, and 4 indicates very much or frequently. Item responses are summed to obtain a final ADHD sum-score (higher scores equal higher ADHD levels).

Anxiety symptoms
Child Sample: Anxiety symptoms were assessed using the Child Behavior CheckList (CBCL) anxiety problems subscale (Achenbach, 1991). The DSM-oriented scale consists of 6 items that assess generalized anxiety disorder, separation anxiety disorder and specific phobia. Mothers were asked to rate 6 items on a 3-point scale where 0=not true, 1=somewhat or sometimes true and 2=very true or often true. Item responses were combined into sum-scores.
Adult sample: Anxiety in adults was assessed using the Adult Self Report (ASR) DSM Anxiety Problems scale (Achenbach & Rescorla, 2003). Participants are asked to report their behavior, thoughts, and feelings of the previous 6 months on 7 items on a 3-point scale where 0=not true, 1=somewhat or sometimes true and 2=very true or often true. The scores were summed to obtain a sumscore where higher scores indicate higher levels of anxiety.

Autistic traits
Autistic traits were measured with the mother-report Child Behavior CheckList (CBCL) autism subscale (So, 2013; Achenbach, 1991). The autism scale consists of 10 items on a 3-point scale; 0: not true, 1: somewhat or sometimes true, and 2: very true or often true. Item responses were combined into a sumscore where higher scores indicate higher levels of autism symptoms.

Depression symptoms
Child sample: symptoms were measured using the mother-report Child Behavior CheckList (CBCL) anxious-depressed subscale (Achenbach, 1991). The Anxious-depressed subscale consists of 13 items pertaining to anxious-depressed symptoms which mothers are asked to rate on a 3-point scale; 0: not true, 1: somewhat or sometimes true, and 2: very true or often true. Item responses were combined into a sums core where higher scores correspond to higher levels of depression.
Adult sample: Depression in adults was measured using the Adult Self-Report DSM Depressive Problems scale (Achenbach & Rescorla, 2003). Participants are asked to report their behavior, thoughts, and feelings of the previous 6 months on 14 items on a 3-point scale where 0=not true, 1=somewhat or sometimes true and 2=very true or often true.

Items were summed to obtain final sum scores where higher scores indicate higher levels of depression.

Neuroticism

This was measured using the short version of the NEO Five Factory Inventory (FFI) Neuroticism scale (Costa & McCrae, 1989). The neuroticism subscale measures neuroticism using 12 items that are scored on a five-point scale (1–5: totally disagree, disagree, neutral, agree and totally agree). The 12 items are summed to obtain a sums core where a higher score indicates higher levels of neuroticism.

Wellbeing

In adults, this was measured using the satisfaction with life scale (Diener et al., 1985). The scale measures well-being using 5 items related to life satisfaction levels on a scale from 1 to 7. The items are summed to obtain a sum score where higher scores of indicate higher levels of well-being.

References

Achenbach, T. M., & Edelbrock, C. (1991). Child behavior checklist. *Burlington (Vt),* 7, 371-392.

Achenbach, T. M., Dumenci, L., & Rescorla, L. A. (2003). DSM-oriented and empirically based approaches to constructing scales from the same item pools. *Journal of clinical child and adolescent psychology, 32*(3), 328-340.

Achenbach, T. M., & Rescorla, L. A. (2003). Manual for the ASEBA adult forms & profiles. Research Center for Children, Youth, & Families, University of Vermont, Burlington, VT, USA.

Kollins, S. H., Epstein, J. N., & Conners, C. K. (2014). Corners' Rating Scales-Revised. In *The use of psychological testing for treatment planning and outcomes assessment* (pp. 215-234). Routledge.

Cantril, H. (1965). The pattern of human concerns. New Brunswick, NJ: Rutgers University Press.

Conners, C. K., Erhardt, D., & Sparrow, E. (1999). Conners' adult ADHD rating scales (CAARS). *Multi-Health Systems, Inc.*

Costa, P. T., & McCrae, R. R. (1989). NEO five-factor inventory (NEO-FFI). Odessa, FL: *Psychological Assessment Resources, 3.*

Diener, E. D., Emmons, R. A., Larsen, R. J., & Griffin, S. (1985). The satisfaction with life scale. *Journal of personality assessment, 49*(1), 71-75.

So, P., Greaves-Lord, K., van der Ende, J., Verhulst, F. C., Rescorla, L., & de Nijs, P. F. (2013). Using the child behavior checklist and the teacher's report form for identification of children with autism spectrum disorders. *Autism,* 17(5), 595-607.

The longitudinal Older Australian Twins Study (OATS)

The longitudinal Older Australian Twins Study (OATS) recruited twins and siblings aged 65 years and older from three eastern Australian states (NSW, Queensland & Victoria). These twins were recruited through the Australian Twin Registry, media release, and newspaper advertisements. Having some basic education in English, with at least low average intelligence (IQ ≥ 80) and the ability to consent to participate along with having a consenting co-twin were the inclusion criteria. Individuals with any life-threatening illness, inadequate English to participate in assessments, and acute psychosis were excluded. More details of the study are reported elsewhere [3,4]. Most of the co-twins were assessed within 6 months duration. Self-report and high-density single-nucleotide polymorphism genotyping arrays were used to confirm zygosity [5].

At baseline, OATS had 623 individuals with 406 females and 217 males, the majority of which were monozygotic and dizygotic twins (96%). Data on demographic, medical, health, and lifestyle measures were assessed at a face-to-face interview.

References

Lee, T., Lipnicki, D. M., Crawford, J. D., Henry, J. D., Trollor, J. N., Ames, D., . . . Sachdev, P. S. (2014). Leisure activity, health, and medical correlates of neurocognitive performance among monozygotic twins: the Older Australian Twins Study. *J Gerontol B Psychol Sci Soc Sci, 69*(4), 514-522. doi:10.1093/geronb/gbt031

Sachdev, P. S., Lammel, A., Trollor, J. N., Lee, T., Wright, M. J., Ames, D., . . . Schofield, P. R. (2009). A comprehensive neuropsychiatric study of elderly twins: the Older Australian Twins Study. *Twin Res Hum Genet, 12*(6), 573-582. doi:10.1375/twin.12.6.573

Sachdev, P. S., Lee, T., Lammel, A., Crawford, J., Trollor, J. N., Wright, M. J., . . . Martin, N. G. (2011). Cognitive functioning in older twins: the Older Australian Twins Study. *Australas J Ageing, 30 Suppl 2*, 17-23. doi:10.1111/j.1741-6612.2011.00534.x

Phenotypes

Depression symptoms

Symptoms were indexed using the 15 items from the Geriatric Depression (Sheikh & Yesavage, 1986).

Anxiety symptoms

Symptoms were indexed using the Goldberg Anxiety Scale (Goldberg,Bridges, Duncan-Jones & Grayson, 1988).

References

Goldberg, D., Bridges, K., Duncan-Jones, P., & Grayson, D. (1988). Detecting anxiety and depression in general medical settings. *Bmj, 297*(6653), 897-899. doi:10.1136/bmj.297.6653.897

Sheikh, J. I., & Yesavage, J. A. (1986). Geriatric Depression Scale (GDS): Recent evidence and development of a shorter version [Press release]

Queensland Institute of Medical Research (QIMR)

Study participants were volunteers recruited into longitudinal studies of Australian twins and their families conducted at QIMR. The contributing cohorts have been described in the articles (PMID: 9403910, 10405086, 19210181, 19803771, 22874079, 23752247, 31198126, 33341723, 33509317, 34924174. DOI: 10.1080/00049530410001734865). Depending on the phenotype, ages in these data ranged from 12 to 94 years and the percentage of females ranged from 54-73%.

Phenotypes

Autistic traits
This was measured as the summed score from 11 items of the Social Responsiveness Scale (Constantino, J., & Gruber, C. P, 2005). Responses were provided on a Likert scale ranging from 1=*false, not at all true* to 4=*very true*). A higher score indicates higher level of Autism type traits.

Depressive symptoms
Symptoms were measured as the summed score from the seven depressions items from the Delusions-Symptoms-States Inventory (DSSI) and five items from the Symptom Checklist 90 that were rescaled to match the DSSI scale. Responses were provided on a Likert scale (1=*not at all*, 2=*a little*, 3=*a lot*, 4=*unbearably*). A higher score indicates more depressive symptoms.

Neuroticism
Neuroticism was measured as the summed score from the neuroticism items from a personality measure. In some studies, one of the versions of the NEO Personality Inventory was administered, which has 240 items measured on a 5-point Likert scale ranging from 1=*strongly disagree* to 5=*strongly agree*. In some studies, one of the versions of the NEO Five Factor Personality Inventory was administered, which has 60 items measured on a 5-point Likert scale ranging from 1=*strongly disagree* to 5=*strongly agree*. In some studies, a Ten Item Personality Measure was administered, and responses were on a 7-point Likert scale ranging from 1=*strongly disagree* to 7=*strongly agree*. A z-score was obtained within questionnaire and this z-score was combined across questionnaires. Only MZ pairs reporting on the same measure and study were included. A higher score indicates a higher level of neuroticism

Wellbeing
Wellbeing was measured by the question "How would you describe your emotional wellbeing?" Responses were provided on a Likert scale (1=*poor*, 2=*fair*, 3=*good*, 4=*excellent*). A higher score indicates a higher level of perceived wellbeing.

References
Constantino, J., & Gruber, C. (2005). The Social Responsiveness Scale Manual. Western Psychological Services, Los Angeles.

The Swedish Twin Registry (STR)

STR is a nation-wide register of twin births with close to full coverage for most of the 20th century and forward (Zagai, Lichtenstein, Pedersen & Magnusson, 2019). Several cohorts of the STR have also been genotyped. Several studies within the STR have contributed to the current study, details of each are below.

References

Zagai, U., Lichtenstein, P., Pedersen, N. L., & Magnusson, P. K. E. (2019). The Swedish Twin Registry: Content and Management as a Research Infrastructure. Twin Research and Human Genetics, 22(6), 672-680. doi:10.1017/thg.2019.99

STR- Child and Adolescent Twin Study in Sweden (CATSS)

Families of all twins born in Sweden beginning in 1992 were contacted in connection with the twins' ninth birthday (earlier cohorts included individuals aged 12 years) and invited to participate in the Child and Adolescent Twin Study in Sweden (CATSS; Anckarsäter et al, 2011). The response rate was 75%. Follow-ups were conducted when the twins were 15 years of age (response rate, 61%) and 18 years of age (response rate, 59%).

References

Anckarsäter, Lundström, Kollberg, et al. The Child and Adolescent Twin Study in Sweden (CATSS). *TwinResHumGenet*.2011;14(6):495-508

Phenotypes

<u>Depression symptoms</u>

Child sample: symptoms were assessed via 11 items from the Short Moods and Feelings Questionnaire (SMFQ, Angold et al 1995) at age 9/12, via telephone interviews with parents. The items included low mood (1), anhedonia (1), tired (1), restless (1), low self worth (6), crying (1), cognitive difficulties (1), loneliness (1) items.
Adult sample: symptoms were assessed at age 18 via 11 self-report items from the Center for Epidemiologic Studies Depression (CESD; Radolff, 1997). The items included appetite (1), low mood (3), tiredness (1), disturbed sleep (1), loneliness (1), others unfriendly (2), enjoyment (1), behavior inactivation (1) items.

<u>ADHD symptoms</u>

Child sample: symptoms were assessed via 19 items from the ADHD subscale of the Autism-Tics, AD/HD and other Comorbidities inventory (A-TAC; Hansson et al, 2005; Larson et al, 2010). The data was obtained via telephone interviews with parents, at age 9/12. The items included Inattentive (9 items) & hyperactive-impulsive (10 items).
Adolescent sample: symptoms were assessed using 5 self-report items from the hyperactivity subscale of the Strengths and Difficulties questionnaire (SDQ; Goodman, 1997)

at age 15. The items included somatic symptoms (1), worries (1), unhappiness (1), nervousness (1), fearfulness (2).

Anxiety symptoms

Child sample: symptoms were assessed using 41 items from the Screen for Anxiety Related Disorders (SCARED, Hale et al 2005). The items included Panic (13), general anxiety (9), separation anxiety (8), school phobia (4) & social phobia (7). Data was collected at ages approximately 9/12 via telephone interviews with parents.
Adult sample: the same measure was used in the adult sample except it was 38 items with 4 instead of 7 for school phobia scale, collected via self-report, at age approximately 18.

Autistic traits

Child sample: symptoms were assessed via 17 items of the ASD subscale of the A-TAC inventory (A-TAC; Hansson et al, 2005; Larson et al, 2010). This included Social interaction (6 items), communication (6 items) & flexible behaviour (5 items) domains. The data was collected via telephone interview with parents when twins were 9/12 years old.
Adult sample: The same measure was used for adults, but with 12 items, via parental reports at age 18.

Psychotic-like experiences

Symptoms were assessed via 7 self-report items from the Adolescent Psychotic-like screener (Laurens et al, 2007) at age approximately 15. The items included Paranoia (4), grandiosity (2), visual hallucinations (1). Example item is: "Sometime I thought being sent special messages through the television" scored as yes, definitely=2, Maybe=1, No, Never=0.

References

Angold, A., Costello, E. J., Messer, S. C., Pickles, A., Winder, F., & Silver, D. (1995). Development of a short questionnaire for use in epidemiological studies of depression in children and adolescents. *International Journal of Methods in Psychiatric Research*(5), 237-249.

Goodman, R. (1997). The Strengths and Difficulties Questionnaire: a research note. *Journal of Child Psychology and Psychiatry, 38*(5), 581-586.

Hale, W. W., 3rd, Raaijmakers Q Fau - Muris, P., Muris P Fau - Meeus, W., & Meeus, W. Psychometric properties of the Screen for Child Anxiety Related Emotional Disorders (SCARED) in the general adolescent population.

Radloff, L. S. (1977). The CES-D Scale: A self-report depression scale for research in the general population. *Applied Psychological Measurement, 1*(3), 385-401.

Hansson S. L, Svanström Röjvall, A., Rastam, M., Gillberg, C., Gillberg, C., Anckarsäter, H. Psychiatric telephone interview with parents for screening of childhood autism - tics, attention-deficit hyperactivity disorder and other comorbidities (A-TAC): preliminary reliability and validity. *Br J Psychiatry*. 2005;187(3):262-267.

Larson,T., Anckarsäter, H., Gillberg,C. et al. The Autism-Tics, AD/HD and Other Comorbidities Inventory (A-TAC): further validation of a telephone interview for epidemiological research. *BMC Psychiatry*. 2010;10(1):1.

Laurens, K. R, Hodgins, S., Maughan, B., Murray, R. M., Rutter M. L., Taylor, E. A. Community screening for psychotic-like experiences and other putative antecedents of schizophrenia in children aged 9-12 years. *Schizophr Res*. 2007;90(1-3):130-146.

The Swedish Twin Registry – Aging Twins PsychChip (STR_Aging)

STR- Aging Twins PsychChip samples included data from monozygotic (MZ) twins reared together from the Swedish Adoption Twin Study of Aging (SATSA), [6], Aging in Women and Men (GENDER) [7], and Origins of Variance in the Oldest Old: Octogenarian Twins (OCTO-Twin) [8] and the Study of Dementia in Swedish Twins (HARMONY) [9]. There was overlap in participation between HARMONY and the SATSA, GENDER, and Octo-Twin studies. Hence, we prioritized data from the earliest home study assessment (SATSA, GENDER, and OCTO-Twin) where available for both members and otherwise used data from HARMONY. Phenotype data for this project were available from an initial set of 321 to 532 STR-Aging MZ twins from SATSA, OCTO-Twin and Harmony. For analyses, we selected data from 204-340 MZ twins who were reared together and per phenotype had data across both members of the pair. The different twin studies held separate-approvals by the regional ethical review board in Stockholm (SATSA: Dnr 80:80, 84:61; 93:226, 98:319, and 2010/657-31/3; OCTO-Twin: Dnr 98:380; HARMONY: Dnr: 97:051 and Dnr 2007/151-31/4). All participants provided informed consents.

Phenotypes

Depressive symptoms

Symptoms were obtained via the Center for Epidemiologic Studies Depression Scale (CES-D) (Radloff, 1977), where the home study assessment was prioritized. Altogether, 61% completed the full 20-item version, using a 4-option response to describe symptoms 'during the last week': 0=never or almost never, 1=rather seldom, 2=rather often and 3=always or almost always. The remaining 39% of the twins responded to an 11-item version using a 4-option response format to describe depressive symptoms over the last week: [0-Rarely/None (less than one day), 1-Some/A Little (1 to 2 days), 2-Occasionally (3 to 4 days), and 3-Most/All the Time (5 to 7 days).] Higher scores represent higher levels of depressive symptoms. CES-D version was used as a covariate (20-item=0/11-item=1). Values were standardized within CES-D version to mean zero and unit standard deviation.

References

Finkel, D., & Pedersen, N. (2004). Processing Speed and Longitudinal Trajectories of Change for Cognitive Abilities: The Swedish Adoption/Twin Study of Aging. Neuropsychol Dev CognB Aging Neuropsychol Cogn, 11(2), 325-345. doi:10.1080/13825580490511152

Gatz, M., Fratiglioni, L., Johansson, B., Berg, S., Mortimer, J. A., Reynolds, C. A., Fiske, A., & Pedersen, N. L. (2005). Complete ascertainment of dementia in the Swedish Twin Registry: the HARMONY study. Neurobiology of Aging, 26(4), 439-447. doi:10.1016/j.neurobiolaging.2004.04.004

Gold, C. H., Malmberg, B., McClearn, G. E., Pedersen, N. L., & Berg, S. (2002). Gender and health: a study of older unlike-sex twins. J Gerontol B Psychol Sci Soc Sci, 57(3), S168-176. Retrieved from http://www.ncbi.nlm.nih.gov/pubmed/11983743

McClearn, G. E., Johansson, B., Berg, S., Pedersen, N. L., Ahern, F., Petrill, S. A., & Plomin, R. (1997). Substantial genetic influence on cognitive abilities in twins 80 or more years old. Science, 276(5318), 1560-1563. Retrieved from http://www.ncbi.nlm.nih.gov/pubmed/9171059

Radloff, L.S. (1977). The CES-D scale: A self-report depression scale for research in the general population. Applied Psychological Measurement, 1, 385-401.

Swedish Twin registry (TwinGene, YATSS, PSYCH)

STR is a nation-wide register of twin births with close to full coverage for most of the 20th century and forward. Several cohorts of the STR have also been genotyped. The data for the current study was from three of these cohorts: the PSYCH, TwinGene and YATSS cohorts. See Zagai et al 2019 for more details. Ethical approval was given by the Uppsala Ethical Review Authority (nr: 2019-06066).

Phenotypes

Wellbeing
Data was collected as part of the SALTY survey and measured as one item a five-point Likert item, with higher values indicating higher well-being. The data was available in two cohorts (TwinGene and PSYCH). The item was "Would you generally describe yourself as: Very happy, Rather happy, Not particularly happy, Not at all happy."

Depression
Symptoms were measured using an 11-item Center for Epidemiologic Studies – Depression scale (CESD; Radloff, 1977) in three different surveys: YATSS, STAGE and STAGE1. The sample from the current study were drawn from the YATSS genotyped cohort only.

References
Radloff, L.S. (1977). The CES-D scale: A self-report depression scale for research in the general population. Applied Psychological Measurement, 1, 385-401

Twins Early Development Study (TEDS)

The Twins Early Development Study (TEDS) is a longitudinal twin study that recruited over 16,000 twin-pairs born between 1994 and 1996 in England and Wales through national birth records. More than 10,000 of these families are still engaged in the study. TEDS was and still is a representative sample of the population in England and Wales. Rich cognitive and emotional/behavioural data have been collected from the twins from infancy to emerging adulthood, with data collection at first contact and at ages 2, 3, 4, 7, 8, 9, 10, 12, 14, 16, 18 and 21, enabling longitudinal genetically sensitive analyses. Data have been collected from the twins themselves, from their parents and teachers, and from the UK National Pupil Database. Genotyped DNA data are available for 10,346 individuals (who are unrelated except for 3320 dizygotic co-twins). TEDS data have contributed to over 400 scientific papers involving more than 140 researchers in 50 research institutions. For the current study, first, all MZ twins with genotype data were selected. Then, of these twins, those who had data on any of the specified phenotypes were taken forward for analyses.

Reference
Rimfeld, K., Malanchini, M., Spargo, T., Spickernell, G., Selzam, S., McMillan, A., ... & Plomin, R. (2019). Twins early development study: A genetically sensitive investigation into behavioral and cognitive development from infancy to emerging adulthood. *Twin Research and Human Genetics*, *22*(6), 508-513.

Phenotypes

ADHD symptoms

Child sample: Hyperactivity subscale of the Strength and difficulties questionnaire (SDQ; Goodman 1997) was used to index hyperactivity symptoms in children. The scale consisted of 5 parent-rated items. The hyperactivity score was a mean of the 5 items, with at least 3 required to be non-missing. Data was collected when twins were approximately 7 years old.
Adolescent sample: Derived from items of the Conners, 2003 measure. This included 18 parent-rated items that assess hyperactivity (9 items) and inattention (9 items). The ADHD score was a total of 18 items, requiring at least 9 items to be non-missing. Data was collected when twins were approximately 16 years old.

Anxiety symptoms

Child sample: anxiety symptoms were assessed using the 5 items from the SDQ Emotional symptoms scales (Goodman 1997). The items were self-rated, and the total score is derived from mean of 5 items, requiring at least 3 items to be non-missing. The data was collected when twins were approximately 7 years old.
Adult sample: anxiety symptoms were assessed via Generalised Anxiety Disorder assessment, 10-item version (GAD10; Craske et al, 2013). A severity measure of current generalised anxiety disorder symptoms. The items were rated by twins. The score was derived from total of 10 items, requiring at least 5 items to be non-missing. The data was collected when twins were approximately 21 years old.

Autistic traits

Child sample: autistic traits were assessed using 30 items from the Childhood Asperger Syndrome Test (CAST; Scott et al, 2002; Williams et al, 2005). The items include both social and non-social domains impaired in autism, rated by parents. Total score is derived from 30 items, requiring at least 15 of the items to be non-missing. Data was collected when twins were approximately 12 years old.
Adolescent sample: autistic traits were assessed using the Autism Quotient (AQ; Baron-Cohen et al, 2001). The measure used here was an abbreviated version that included 28 items constituting 4 subscales: attention-switching (8 items), imagination (6 items), attention to detail (5 items), social (9 items). The questionnaire was rated by parents. The autistic traits score is a total of 28 items, requiring at least 14 to be non-missing. Data was collected when twins were approximately 16 years old.

Depression symptoms

Child sample: depression symptoms were assessed via the MFQ scale. The depression score is a mean of 11 self-report items, requiring at least 6 items to be non-missing. Data was collected when twins were approximately 12 years old.
Adult sample: the same measure as above was used, with 8 items, requiring at least 4 to be non-missing. Data was collected when twins were approximately 21 years old.

Neuroticism

This was measured with the Big-Five personality questionnaire (Mullins-Sweatt, Jamerson, Samuel, Olson, Widiger (2006), a 6-item self-report psychometrically validated inventory devised to measure five personality traits including neuroticism. The neuroticism score is a

mean of 6 of the items, requiring at least 3 of them to be non-missing. The responses are on a Likert rating scale ranging from 1 to 5, with higher scores representing higher levels of neuroticism. The data was collected when twins were approximately 21 years old.

Psychotic-like experiences

This was assessed via 10 items drawn from a questionnaire that has been previously used to examine prevalence and quality of psychotic like experiences in community samples (Stefanis, 2002). These 10 items assess whether participants have experienced symptoms, specifically positive symptoms (grandiosity/delusions, hallucinations and paranoia), that are typically associated with psychosis. For example," *have you ever felt that someone else's thoughts were being inserted into your head against your will*?", "*Have you ever believed that you were being sent special messages through the television or the radio, or that a programme had been arranged just for you alone?* ", and, "*Have you ever felt that you are somebody really special, or that you have special powers like reading people's mind, or that you have been chosen to perform great and special tasks?*". The items were rated as "yes, definitely=2", "Yes maybe=1", "No, never=0", and added up to obtain a total score, with higher scores reflecting higher presence of Psychotic-like experiences. The data was collected when twins were approximately 16 years old.

Wellbeing

Brief Multidimensional Students' Life Satisfaction Scale (BMSLSS; Seligson et al 2003) was used to assess general satisfaction with life, indicating one's subjective wellbeing. This is a self-report measure consisting of 6 items rated on a Lickert scale.
Wellbeing score is a mean of 6 items, requiring at least 3 to be non-missing. The data was collected when twins were approximately 16 years old.

References

Baron-Cohen, S., Wheelwright, S., Skinner, R., Martin, J., and Clubley, E. (2001) The autism-spectrum quotient (AQ): evidence from Asperger syndrome/high-functioning autism, males and females, scientists and mathematicians. J Autism Dev Disord 31(1): 5-17

Conners C.K. (2003): Conners' Rating Scales-Revised: Technical Manual. New York: Multi-Health System Inc.

Craske M, Wittchen U, Bogels S, Stein M, Andrews G, Lebeu R. (2013). Severity measure for generalized anxiety disorder, Adults. American Psychiatric Association.

Goodman, R (1997): The Strengths and Difficulties Questionnaire: A research note; Journal of Child Psychology and Psychiatry, 38, 581-586.

Mullins-Sweatt, S. N., Jamerson, J. E., Samuel, D. B., Olson, D. R., & Widiger, T. A. (2006). Psychometric properties of an abbreviated instrument of the five-factor model. *Assessment, 13*(2), 119-137.

Scott, F. J., Baron-Cohen, S., Bolton, P., & Brayne, C. (2002). The CAST (Childhood Asperger Syndrome Test): Preliminary development of a UK screen for mainstream primary-school-age children. Autism, 6, 9-31.

Seligson, J., Huebner, E., & Valois, R. (2003). Preliminary validation of the Brief Multidimensional Students' Life Satisfaction Scale (BMSLSS). Social Indicators Research, 61, 121-145.

Stefanis, N. C., Hanssen M Fau - Smirnis, N. K., Smirnis Nk Fau - Avramopoulos, D. A., Avramopoulos Da Fau - Evdokimidis, I. K., Evdokimidis Ik Fau - Stefanis, C. N., Stefanis Cn Fau - Verdoux, H., . . . Van Os, J. (2002). Evidence that three dimensions of psychosis have a distribution in the general population.

Williams, J., Scott, F., Stott, C., Allison, C., Bolton, P., Baron-Cohen, S., & Brayne, C. (2005). The CAST (Childhood Asperger Syndrome Test): test accuracy. Autism, 9 (1), 45-68.

TwinsUK

TwinsUK is large population-based study of twins in the United Kingdom started in 1992 (Spector & MacGregor, 2002; Spector & Williams, 2006). The data comprises a wide range of phenotypes and biological data, for about 15,000 MZ and DZ adult twins (www.twin-research.ac.uk). For the current study, the participants included a subset of the sample, where data had been collected on depression, anxiety or wellbeing phenotypes. Depression and anxiety data were collected in once in 2002 (Twin Health Questionnaire) and again in 2017 (Questionnaire Supplement). The data for the current study was drawn from 2002 wave, as the sample was larger than the one from 2017. K10 was collected part of the Autumn Questionnaire (2010). All of the questionnaires were sent by post (paper) and completed by the participant.

References

Spector, T. D., & MacGregor, A. J. (2002). The St. Thomas' UK adult twin registry. *Twin Research and Human Genetics, 5*(5), 440-443.

Spector, T. D., & Williams, F. M. K. (2006). The UK adult twin registry (TwinsUK). *Twin Research and Human Genetics, 9*(6), 899-906.

Phenotypes

Depression & Anxiety

Symptoms were assessed using Hospital Anxiety and Depression scale (HADS; Zigmond & Snaith, 1983). This included 7 items assessing depression symptoms and 7 items assessing anxiety. Items were self-reported and rated on a Lickert scale ranging from not at all=0 to most of the time=3. The total score for the scale was derived such that higher scores reflected higher levels of anxiety and depression.

Wellbeing

Wellbeing was assessed using 6 self-report items from Kessler Psychological Distress Scale (K10; Kessler, Andrews, Colpe, 2002; Andrews & Slade, 2001). The items measured to what extent the participants felt positive emotions such as satisfied, in good spirits, calm and peaceful, in the past month. The items were self-report, rated on a scale of none of the time=1 to all of the time =5. Higher scores indicated higher levels of wellbeing.

References

Andrews, G., Slade, T (2001). Interpreting scores on the Kessler Psychological Distress Scale (k10). Australian and New Zealand Journal of Public Health, 25, 494-497.

Kessler, R.C., Andrews, G., Colpe, .et al (2002) Short screening scales to monitor population prevalences and trends in non-specific psychological distress. Psychological Medicine, 32, 959-956.

Zigmond, A. S., & Snaith, R. P. (1983). The hospital anxiety and depression scale. Acta Psychiatr Scand, 67(6), 361-370.

Table S1. Participating studies N per phenotype

Phenotype	Sample	TEDS	NTR	STR_CATSS	FTC	QIMR	STR_psy	STR_ytss	STR_tg	STR_aging	TwinsUK	DTR	MTR	OATS	N
ADHD	largest	4290	2430	6506	512										13738
	child	4290	2430	6506	512										13738
	adolescent*	3018	1652	2832	338										7840
Anxiety	Largest	3490	3024	3980							1590			270	12354
	child	3490	3024	3980											10494
	adult	2484	1558	2030							1590			270	7932
Autistic	largest	3578	3036	6516		1022									14152
	child	3578	3036	6516											13130
	adult**	3018		2010		1022									6050
Depression	largest	3502	3024	3984	1912	2392		3714		280	1592	782	340	270	21792
	child	3502	3024	3984											10510
	adult	2482	2348	1962	1912	2392		3714		280	1592	782	340	270	18074
Neuroticism	largest	2364	2708		2816	1012									8900
Psychotic-like experiences	largest	842		2794											3636
Wellbeing	largest	2946	2696		3218	254	1122		886		1460	782	376		13740

ADHD: attention-deficit hyperactivity disorder symptoms; Largest: largest available sample, obtained by selecting the largest sample from each study, irrespective of age group. Child: data from studies where participants were aged 5-12 years old; Adolescent: data from studies where participants were aged 13-18 years old; Adult: data from studies where participants were aged >18 years old; *NTR data from adults; **TEDS data from adolescents; rMZ= Monozygotic twin correlation

2. Study-level GWAS of MZ differences and QC

2.1. GWAS model

The analysis plan was shared with interested cohorts to conduct GWAS of MZ differences on their available phenotypes using automated scripts (https://github.com/LaurenceHowe/MZTwins-vQTL). Plink 1.9 was recommended for conducting GWAS.

GWAS model: The underlying model involved a regression of the phenotypic difference between MZ twins on the genetic marker. First, the absolute phenotypic differences (APD) were obtained per twin pair. Next, the APD was residualised for sex, age and 10 principal components (PCs), and the residuals were inverse rank transformed and standardised. This transformed variable was used as the predictor in the following model:

Model 1:

$$|y_{i,A} - y_{i,B}| \sim \beta_{2,j} G_{ij} + \epsilon_{i,j}$$

Sensitivity analyses included two further models for comparison with Model 1:

Model 2: Model 1 and control for within-pair mean
Model 3: Model 1 but phenotype is not residualised on principal components.

The difference between model 2 and model 1 was the inclusion of within-pair mean as a control variable in the regression. Model 2 was constructed to examine if adjusting for within-twin mean in the GWAS model would significantly impact the SNP-associations, which would be the case if the MZ differences largely reflected mean effects. However, this also risks over-correcting, especially for vQTLs, which affect both the mean and variance of a phenotype, which was indicated here by small to moderate ($r \sim 0.3$ to 0.6) positive correlation between MZ phenotypic mean and MZ phenotypic differences in several of our samples and as previously proposed [10]. We constructed Model 3, which differed from our primary model by not adjusting for Principal Components when constructing the phenotype. This was done because some of the participating studies were likely to be very small (<300) and the blanket inclusion of 10 PC for all studies might have been overly conservative, leading to significant under-inflation of p-values.

We used Sign-test to assess whether Model 2 and Model 3 results were similar to Model 1, in which case, we would consider our primary model to be the most parsimonious: lower number of parameters than, but similar results to Model 2, while also correcting for population stratification confounding. We used the available script from EasyQC to assess the correlation between p-values and betas of Model 1 with Model 2 and 3. We also examined if the direction of effects (BETAs) were significantly similar, using the script available here: https://github.com/klpurves/SignTest. This compares the direction of effects at various p-value threshold using the following steps: clump base file SNPs using 1000 Genomes as reference, create SNP list based on specified p-value threshold, merge base and target data retaining independent SNPs in base data, perform SNP test and exact binomial test

(probability .5, two -sided). The results indicated that the effects were largely in the same direction (See section 3.2: Table S3a, S3b, S3c).

2.2. Study-level GWAS QC

Study-level GWAS results were QC'd using EasyQC (v. 23.8)[11]. SNPs with missing BETA, SE, P or INFO score, as well as SNPs with MAF <.01, INFO score < 0.5 were removed. Using cptid format, alleles were harmonised according to their respective reference panel (1000 G Phase 3 version 5 or Haplotype Reference Consortium), effect and other alleles, using mapping and frequency files available in EASYQC:

HRC.r1-1.GRCh37.wgs.mac5.sites.tab.cptid.maf001.gz

HRC.r1-1.GRCh37.wgs.mac5.sites.tab.rsid_map.gz

1000GP_p3v5_legends_rbind.noDup.noMono.noCnv.noCnAll.afref.EUR.txt.gz

rsmid_machsvs_mapb37.1000G_p3v5.merged_mach_impute.v3.corrpos.gz

SNPs with mismatching alleles to the reference panel were removed. Indels, monomorphic SNPs and duplicate SNPs, that could also be tri-allelic (same bp position with different alleles) were removed, retaining only one SNP with the largest sample. QQ-plots and lambda-median values were inspected for p-value inflation in each dataset. Figure S2-S6 show QQ-plots of study-level GWAS of each phenotype and Table S2 shows QC information, including median-Lambda values. One study did not pass the QC procedure due to low N (<100) and missingness across a large number of SNPs (>20%) and was therefore excluded from meta-analyses.

Table S2. Study Level information and QC

Phenotype	Study	numVarIn	numVarOut	corr between ref.panel MAF and study EAF	N_max	Lambda.PVAL.GC	NSNP P<5x10-5	NSNP P<5x10-6	NSNP P<5x10-7	NSNP P<5x10-8
ADHD	CATSS_adolescent	7506235	7506235	0.997	1416	1.007	397	35	9	0
	CATSS_child	7578299	7578298	0.998	3253	1.025	368	25	0	0
	FTC_adolescent	7836429	6983059	0.988	169	0.997	341	19	0	0
	FTC_child	7836429	7603522	0.989	256	0.996	201	16	0	0
	NTR_adult	8549069	7543722	0.997	826	1.002	402	7	0	0
	NTR_child	8571055	7563855	0.997	1215	1.000	269	9	0	0
	TEDS_adult	6815364	6815364	0.999	1509	0.997	302	31	0	0
	TEDS_child	6815364	6815364	0.999	2145	0.999	312	54	0	0
Anxiety	CATSS_adult	7481313	7481313	0.997	1015	0.998	261	44	0	0
	CATSS_child	7553169	7553168	0.998	1990	0.993	273	26	0	0
	NTR_adult	8553452	7547736	0.997	779	1.007	295	25	0	0
	NTR_child	8576246	7568419	0.997	1512	1.018	314	35	7	0
	OATS	6800060	6033895	0.995	135	1.001	320	8	0	0
	TEDS_adult	6815364	6815364	0.999	1242	0.992	356	15	1	0
	TEDS_child	6815364	6815364	0.999	1745	1.009	222	4	0	0
	TwinsUk	7552852	7552839	0.997	795	1.011	531	59	2	0
Autistic traits	CATSS_adult	7474267	7474267	0.997	1005	1.006	423	35	1	0
	CATSS_child	7579932	7579931	0.998	3258	1.053	598	76	1	0
	NTR_child	8578625	7570615	0.997	1518	0.999	505	27	5	0
	QIMR	7031777	7027679	0.993	511	1.007	270	25	3	0
	TEDS_adult	6815364	6815364	0.999	1509	1.008	350	48	1	0
	TEDS_child	6815364	6815364	0.999	1789	1.008	313	36	10	0
Depression	CATSS_adult	7490111	7490111	0.997	981	1.001	336	24	0	0
	CATSS_child	7553734	7553733	0.998	1992	1.052	763	93	8	4
	DTR	4807249	4798517	0.994	391	1.001	200	19	0	0
	FTC	7836429	7836422	0.990	956	1.007	318	28	0	0
	NTR_adult	8566778	7559622	0.997	1174	1.008	355	35	0	0
	NTR_child	8576299	7568466	0.997	1512	0.998	525	82	0	0
	OATS	6800060	6033895	0.995	135	1.021	431	107	5	0
	QIMR	7046740	7042886	0.993	1196	1.002	365	41	2	1
	STR_aging	6755261	5515015	0.992	140	0.987	148	5	4	0
	STR3	7682443	7602277	0.998	1840	0.997	367	25	2	0
	TEDS_adult	6815364	6815364	0.999	1241	1.004	400	18	0	0
	TEDS_child	6815364	6815364	0.999	1750	1.010	252	25	0	0
	TwinsUK	7553824	7553811	0.997	796	1.003	489	24	1	0
Neuroticism	FTC	7836429	7836422	0.990	1408	0.997	355	87	0	0
	NTR	8564642	7557696	0.997	1354	0.993	319	20	2	0
	QIMR	7020765	7016489	0.993	506	1.001	322	15	1	0
	TEDS	6815364	6814427	0.999	1182	0.997	319	63	4	0
psychotic-like experiences	CATSS	7520891	7520891	0.997	1396	1.010	373	31	0	0
	TEDS	6815364	6815334	0.999	421	0.993	297	20	1	0
Wellbeing	DTR	4807249	4798517	0.994	391	0.994	176	1	0	0
	FTC	7836429	7836422	0.990	1609	1.011	587	67	18	5
	NTR	8583598	7575072	0.997	1348	1.000	248	8	1	0
	QIMR	6235402	5178397	0.991	127	1.000	250	15	0	0
	STR1	7592964	7535087	0.998	553	1.003	419	67	12	0
	STR2	7641015	7542478	0.997	443	1.003	363	65	0	0
	TEDS	6815364	6815364	0.999	1487	1.008	379	101	0	0
	TwinsUK	7532362	7532350	0.997	730	0.996	326	77	0	0

Figures S2 – S6: Study-level QQ-Plots of GWAS of MZ differences

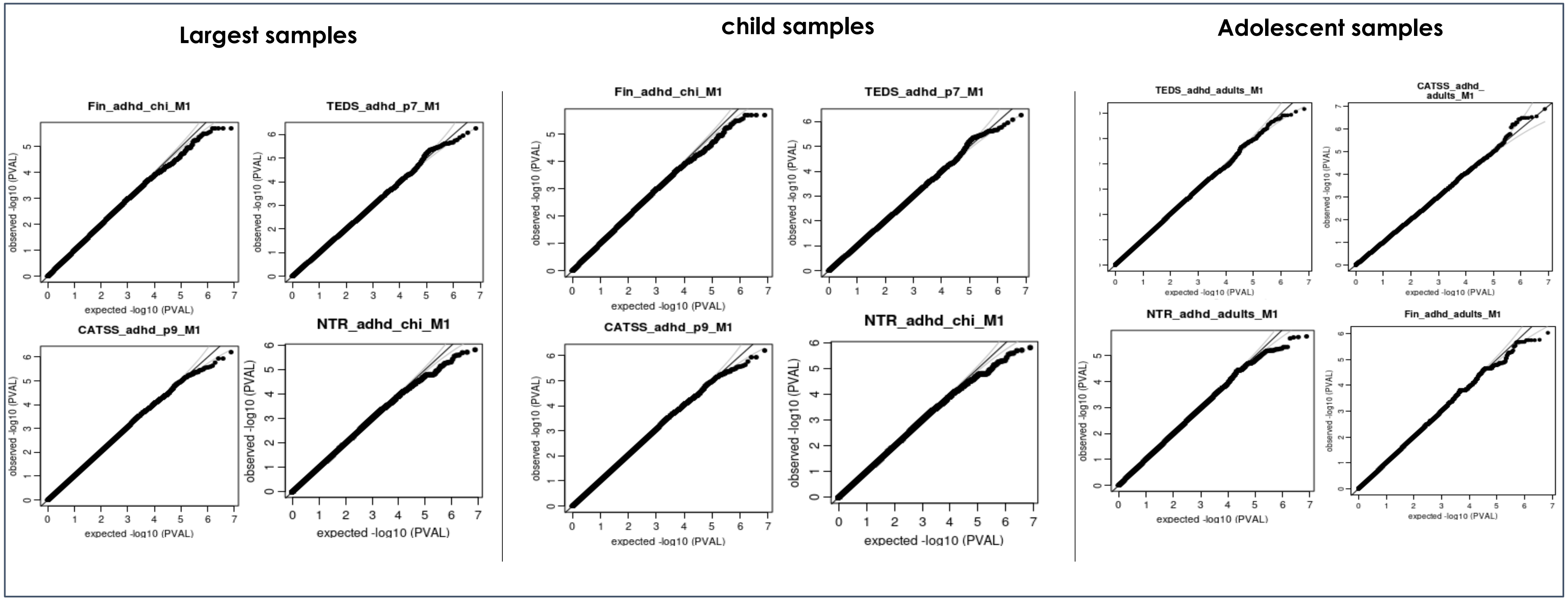

Figure S2. QQ-Plots of p-values from MZ GWAS of ADHD symptoms

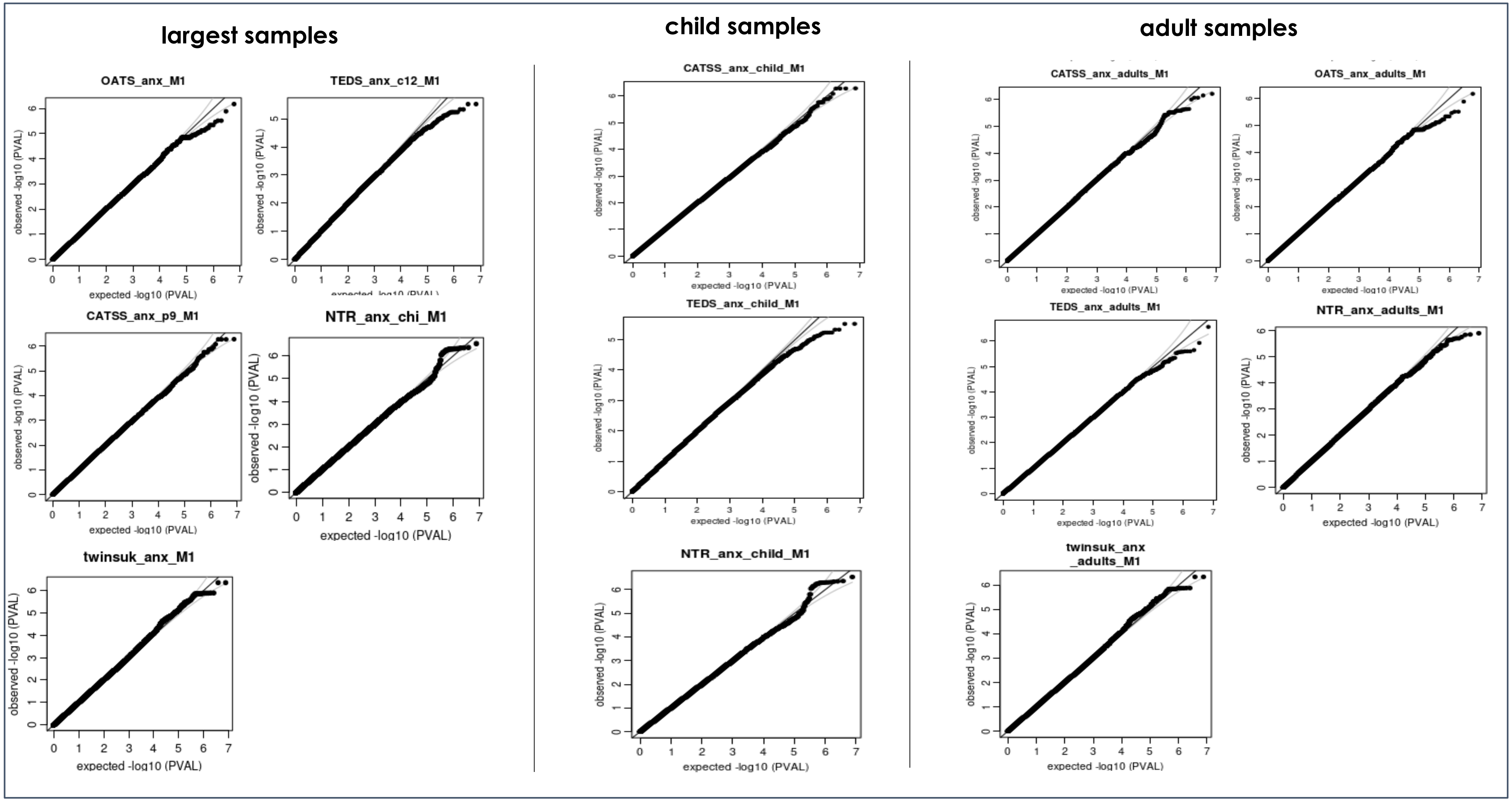

Figure S3. QQ-Plots of p-values from MZ GWAS of anxiety symptoms

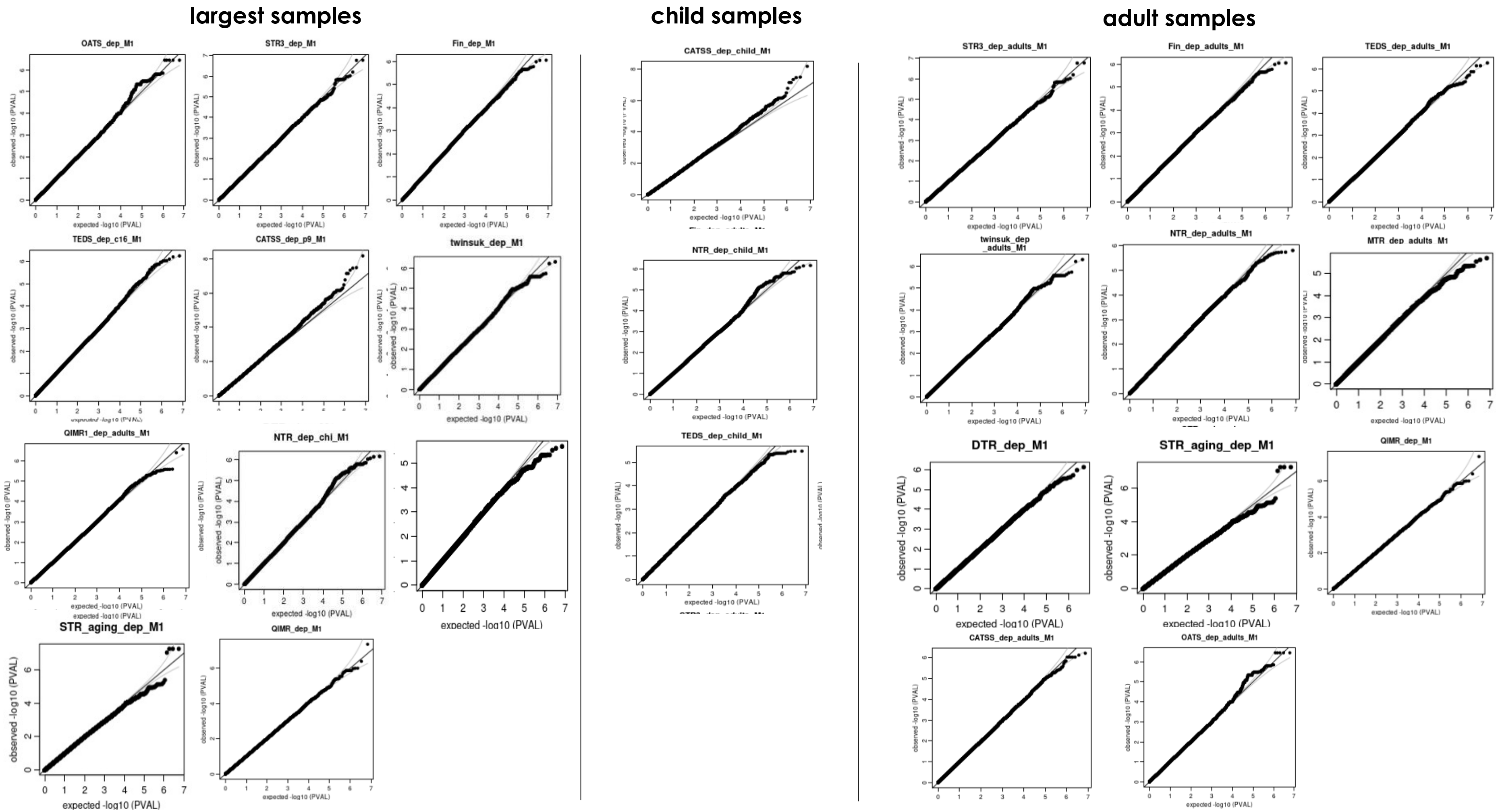

Figure S4. QQ-Plots of p-values from MZ GWAS of depression symptoms

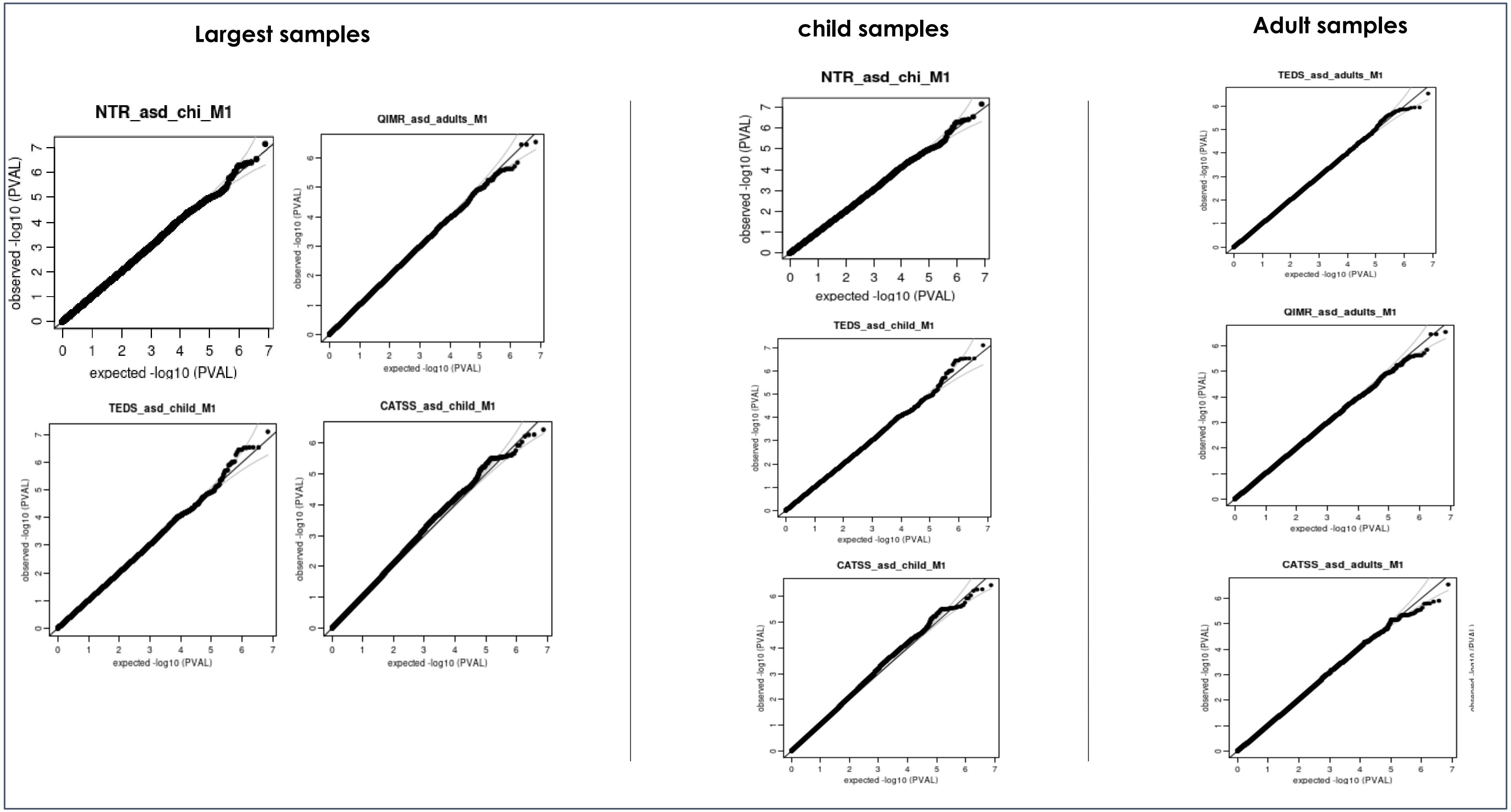

Figure S5. QQ-Plots of p-values from MZ GWAS of autistic traits

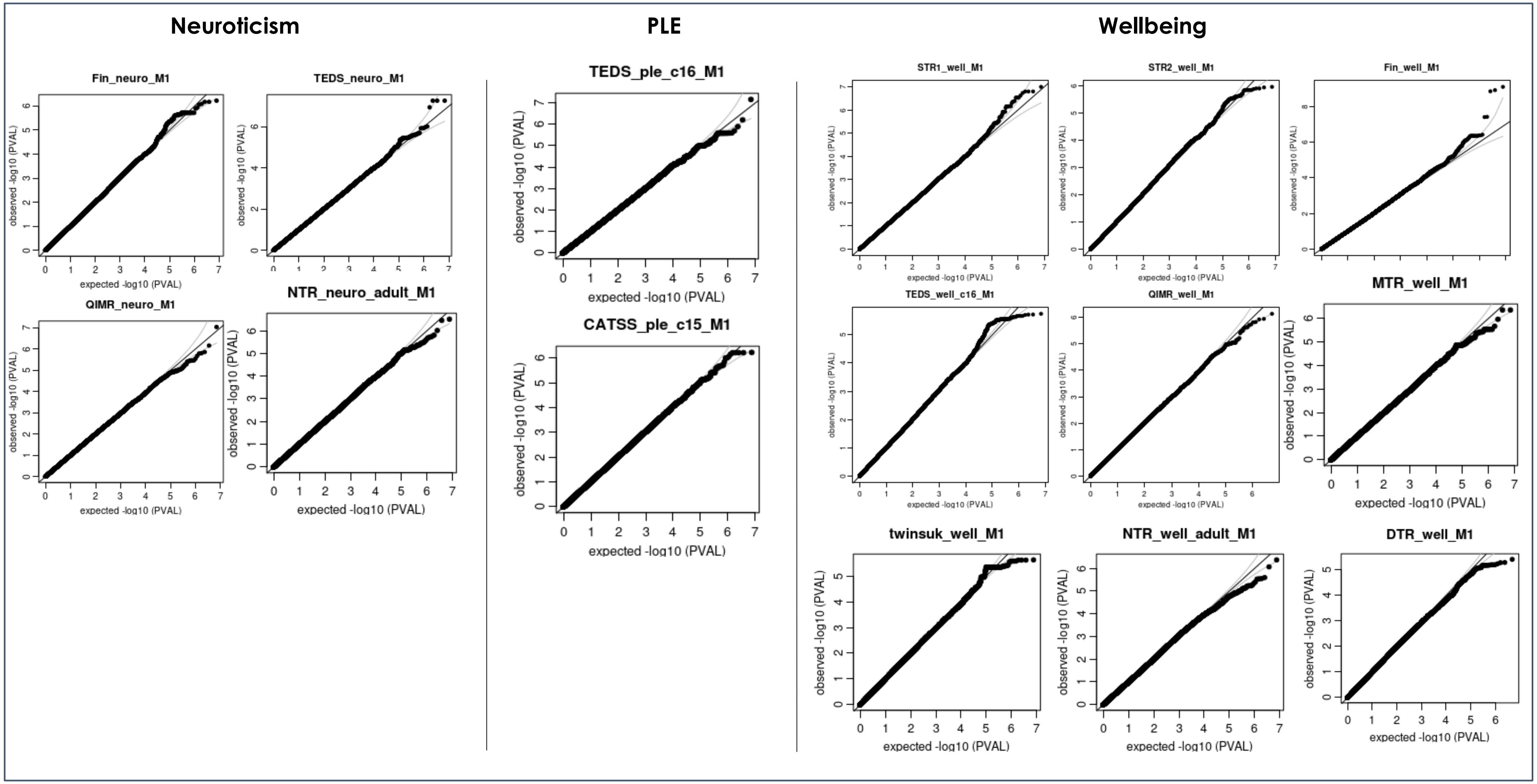

Figure S6. QQ-Plots of p-values from MZ GWAS of neuroticism, psychotic-like experiences and wellbeing

3. GWAS meta-analyses

METAL (2011 release) [12] was used to conduct inverse-variance weighted fixed effect meta-analysis on QC'd GWAS results for each phenotype.

First, a meta-analysis was conducted on largest sample for each phenotype, by including the largest sampled GWAS results from each study regardless of developmental stage. This was done for Model 1 (primary model), as well as Model 2 (adjusted for within-twin mean) & Model 3 (no PC or within-twin adjustment).

The results of Model 2 and 3 were compared to our primary Model 1, using Sign-test. This examined the correlation between p-values and BETAs in Model 1 and Model 2 or 3, as well as comparing the direction of effects at various p-value thresholds (see supplementary information Section 2.1). The results of Sign-test, comparing Model 1 with Model 2 and 3 indicated that the effects were largely in the same direction (see Table S3a, S3b, S3c). The remaining analyses were therefore conducted using Model 1 results only, which made no-adjustment for within-twin mean but accounted for population stratification.

Second, GWAS meta-analysis were conducted separately in developmentally-stratified samples for depression (child, adult), anxiety (child, adult), ADHD (child, adolescent), and ASD (child, adult) phenotypes.

"ANALYZE HETEROGENEITY ON" option in METAL was used to examine whether the effect sizes (test statistics) were significantly different across studies.

"AVERAGEFREQ ON", "MINMAXFREQ ON", "LABEL TotalSampleSize as N" options were used to keep track of effect allele frequencies across studies, obtain an average allele frequency and total sample size across studies.

"ADDFILTER N > 100" and "ADDFILTER MAF > 0.01" were used to include only SNPs with N>100 and MAF >.01 from studies.

The chr:bp:a1:a2 ids of the meta-analysis results were converted to rs ids of SNPs in 1000 genome phase 3 version 5 European panel (available from www.genepi-regensburg.de/easyqc:rsmid_machsvs_mapb37.1000G_p3v5.merged_mach_impute.v3.corrpos). SNP2Gene function within FUMA web application (v1.5.2) [13] was used to annotate GWAS SNPS and identify independent significant SNPs (SNPs which are in LD of the lead SNP at r^2 0.1; lead SNPs are those in LD with any of independent significant SNPs with r^2 >.6), and for producing regional plots and QQ and Manhattan plots.
Figures S7 to S11 show QQ and Manhattan plots of p-values. **Table S4** shows the top SNP for each phenotype. See **Table S5** for parameters used in FUMA for annotation of GWAS meta-analysis results.

Tables S3a – S3c: GWAS model comparisons

Table S3a. GWAS Models Comparison. M1 & M2						
pthreshold	Base Model	Target Model	Total_SNPs	Total_Shared	Proportion in the same direction	Binomial_test
ADHD						
5.10E-08	ADHD M1	ADHD M2	0	0	NA	NA
5.10E-06	ADHD M1	ADHD M2	9	9	1	3.91E-03
0.00051	ADHD M1	ADHD M2	806	806	1	4.69E-243
0.051	ADHD M1	ADHD M2	25598	25596	1	4.95e-324
0.51	ADHD M1	ADHD M2	80394	79486	0.99	9.89e-324
1	ADHD M1	ADHD M2	99230	93856	0.95	9.89e-324
Anxiety						
5.10E-08	Anxiety M1	Anxiety M2	0	0	NA	NA
5.10E-06	Anxiety M1	Anxiety M2	7	7	1	1.6E-02
0.00051	Anxiety M1	Anxiety M2	651	651	1	2.1E-196
0.051	Anxiety M1	Anxiety M2	21156	21156	1	4.95e-324
0.51	Anxiety M1	Anxiety M2	68174	67507	0.99	4.95e-324
1	Anxiety M1	Anxiety M2	84133	79693	0.95	9.89e-324
ASD						
5.10E-08	ASD M1	ASD M2	0	0	NA	NA
5.10E-06	ASD M1	ASD M2	11	11	1	9.77E-04
0.00051	ASD M1	ASD M2	830	830	1	2.79E-250
0.051	ASD M1	ASD M2	24501	24499	1.00	4.95e-324
0.51	ASD M1	ASD M2	76378	75322	0.99	9.89e-324
1	ASD M1	ASD M2	94381	88880	0.94	9.89e-324
Depression						
5.10E-08	Depression M1	Depression M2	0	0	NA	NA
5.10E-06	Depression M1	Depression M2	12	12	1	4.88E-04
0.00051	Depression M1	Depression M2	754	754	1	2.11E-227
0.051	Depression M1	Depression M2	22586	22582	0.999823	4.95e-324
0.51	Depression M1	Depression M2	71393	70017	0.980726	9.89e-324
1	Depression M1	Depression M2	87924	82028	0.932942	9.89e-324
Neuroticism						
5.10E-08	Neuroticism M1	Neuroticism M2	0	0	NA	NA
5.10E-06	Neuroticism M1	Neuroticism M2	13	13	1	2.4E-04
0.00051	Neuroticism M1	Neuroticism M2	1014	487	0.48	2.2E-01
0.051	Neuroticism M1	Neuroticism M2	24433	17742	0.73	4.95e-324
0.51	Neuroticism M1	Neuroticism M2	58827	49989	0.85	4.95e-324
1	Neuroticism M1	Neuroticism M2	68547	59012	0.86	9.89e-324
Psychotic Like experiences (PLE						
5.10E-08	PLE M1	PLE M2	0	0	NA	NA
5.10E-06	PLE M1	PLE M2	8	8	1	7.8E-03
0.00051	PLE M1	PLE M2	645	645	1	1.4E-194
0.051	PLE M1	PLE M2	19648	19647	1	4.95e-324
0.51	PLE M1	PLE M2	66183	64846	0.98	9.89e-324
1	PLE M1	PLE M2	82775	77014	0.93	9.89e-324
Wellbeing						
5.10E-08	Wellbeing M1	Wellbeing M2	1	1	1	1.00E+00
5.10E-06	Wellbeing M1	Wellbeing M2	16	16	1	3.05E-05
0.00051	Wellbeing M1	Wellbeing M2	714	714	1	2.32E-215
0.051	Wellbeing M1	Wellbeing M2	21471	21465	1	4.95e-324
0.51	Wellbeing M1	Wellbeing M2	68304	66827	0.98	9.89e-324
1	Wellbeing M1	Wellbeing M2	84228	78497	0.93	9.89e-324

Table S3b. GWAS Models Comparison. M1 & M2

pthreshold	Base_sample	Target_sample	Total_SNPs	Total_Shared	Proportion	Binomial_test
5.10E-08	ADHD M1	ADHD M3	0	0	NA	NA
5.10E-06	ADHD M1	ADHD M3	9	9	1	3.9E-03
0.00051	ADHD M1	ADHD M3	806	806	1	4.69E-243
0.051	ADHD M1	ADHD M3	25598	25598	1	4.69E-243
0.51	ADHD M1	ADHD M3	80394	80100	1	9.89e-324
1	ADHD M1	ADHD M3	99230	95443	0.96	9.89e-324
Anxiety						
5.10E-08	Anxiety M1	Anxiety M3	0	0	NA	NA
5.10E-06	Anxiety M1	Anxiety M3	7	7	1	1.6E-02
0.00051	Anxiety M1	Anxiety M3	651	651	1	2.14E-196
0.051	Anxiety M1	Anxiety M3	21197	21197	1	4.95e-324
0.51	Anxiety M1	Anxiety M3	68331	68275	1	9.89e-324
1	Anxiety M1	Anxiety M3	84337	81932	0.97	9.89e-324
ASD						
5.10E-08	ASD M1	ASD M3	0	0	NA	NA
5.10E-06	ASD M1	ASD M3	11	11	1	9.8E-04
0.00051	ASD M1	ASD M3	830	830	1	2.79E-250
0.051	ASD M1	ASD M3	24501	24501	1	4.95e-324
0.51	ASD M1	ASD M3	76378	76227	1	9.89e-324
1	ASD M1	ASD M3	94381	91152	0.97	9.89e-324
Depression						
5.10E-08		Depression M3	0	0	NA	NA
5.10E-06	Depression M1	Depression M3	12	12	1	4.88E-04
0.00051	Depression M1	Depression M3	754	754	1	2.11E-227
0.051	Depression M1	Depression M3	22612	22612	1	4.95e-324
0.51	Depression M1	Depression M3	71534	70963	0.992018	9.89e-324
1	Depression M1	Depression M3	88116	83847	0.951552	9.89e-324
Neuroticism						
5.10E-08	Neuroticism M1	Neuroticism M3	0	0	NA	NA
5.10E-06	Neuroticism M1	Neuroticism M3	13	13	1	2.4E-04
0.00051	Neuroticism M1	Neuroticism M3	1014	479	0.47	8.4E-02
0.051	Neuroticism M1	Neuroticism M3	24433	17209	0.70	4.95e-324
0.51	Neuroticism M1	Neuroticism M3	58828	46080	0.78	9.89e-324
1	Neuroticism M1	Neuroticism M3	68549	53815	0.79	9.89e-324
Psychotic Like experiences (PLE						
5.10E-08	PLE M1	PLE M3	0	0	NA	NA
5.10E-06	PLE M1	PLE M3	8	8	1	7.8E-03
0.00051	PLE M1	PLE M3	645	645	1	1.4E-194
0.051	PLE M1	PLE M3	19648	19648	1	4.95e-324
0.51	PLE M1	PLE M3	66183	65314	0.99	9.89e-324
1	PLE M1	PLE M3	82775	77839	0.94	9.89e-324
Wellbeing						
5.10E-08	Wellbeing M1	Wellbeing M3	1	1	1	1.00
5.10E-06	Wellbeing M1	Wellbeing M3	16	16	1	0.00
0.00051	Wellbeing M1	Wellbeing M3	714	714	1	2E-215
0.051	Wellbeing M1	Wellbeing M3	21471	21471	1	4.95e-324
0.51	Wellbeing M1	Wellbeing M3	68304	68280	1	9.89e-324
1	Wellbeing M1	Wellbeing M3	84228	82270	0.98	9.89e-324

Table S3c. Model comparison: M1 & M3

Phenotype	corr_P	corr_BETA
Model 1&2		
ADHD	0.73	0.89
Anxiety	0.70	0.87
ASD	0.68	0.86
Depression	0.64	0.84
Neuroticism	0.92	0.97
PLE	0.64	0.84
Wellbeing	0.62	0.81
Model 1 & 3		
ADHD	0.78	0.90
Anxiety	0.85	0.94
ASD	0.80	0.92
Depression	0.75	0.90
Neuroticism	0.67	0.86
PLE	0.68	0.86
Wellbeing	0.89	0.96

Table S4. Top SNPs per phenotype

Phenotype	Sample	SNP	CHR	Position	Gene	A1	MAF	Beta	SE	P	N	Effect across studies
ADHD	Largest	rs62440140	7	7017333	*AC079804.1*	A	0.8	0.12	0.02	2.00E-07	5654	?+++
	Child	rs62440140	7	7017333	*AC079804.1*	A	0.8	0.12	0.02	2.00E-07	5654	?+++
	Adolescent	rs146735996	5	118359106	*RP11-655H13.2*	A	0.97	0.4	0.08	8.00E-07	3751	+?++
Anxiety	Largest	rs2279849	15	90444639	*C15orf38-AP3S2:C15orf38*	A	0.27	0.11	0.02	1.25E-07	6177	+++++
	Child	rs148140018	17	61028968	*RP11-180P8.1*	T	0.03	0.49	0.10	4.76E-07	1990	?+?
	Adult	**rs60358762**	**13**	**99411217**	***SLC15A1***	**A**	**0.03**	**0.44**	**0.08**	**5.07E-09**	**3033**	**+++??**
autistic traits	Largest	rs9624813	22	25784853	*LRP5*	A	0.19	-0.13	0.03	2.00E-07	5049	---?
	Child	rs116949133	9	138821760	*UBAC1*	A	0.01	-0.58	0.12	5.00E-07	3258	?-?
	Adult	rs139326350	16	70940065	*HYDIN*	T	0.04	0.33	0.06	2.00E-07	2978	+++
Depression	Largest	rs16909904	9	98236865	*PTCH1*	T	0.10	0.12	0.02	6.72E-08	10844	++++-+++-++
	Child	rs10905596	10	9677682	*HSP90AB7P*	T	0.34	-0.11	0.02	2.28E-07	5249	---
	Adult	rs7963939	12	47465082	*AMIGO2*	A	0.01	-0.12	0.02	2.35E-07	8248	-?---------
Neuro	Adult	rs72823287	16	81023480	*CMC2*	C	0.82	-0.25	0.04	5.00E-07	1408	?-??
Wellbeing	Adult	**rs2940988**	**4**	**37586376**	***C4orf19***	**T**	**0.88**	**0.16**	**0.03**	9.93E-09	**6464**	++?++-+++
PLE	Adolescent	rs7600318	2	206522214	*NRP2*	A	0.54	-0.16	0.03	6.00E-07	1817	--

Notes: Largest: largest available sample, obtained by selecting the largest sample from each study, irrespective of age group. Child: data from studies where participants were aged 5-12 years old; Adolescent: data from studies where participants were aged 13-18 years old; Adult: data from studies where participants were aged >18 years old. Results with genome-wide significant p-value are highlighted in bold. ? in the effect column indicates the SNP was not available in the respective study.

Figures S7-S11: Manhattan and QQ plots of MZ GWAS meta-analyses

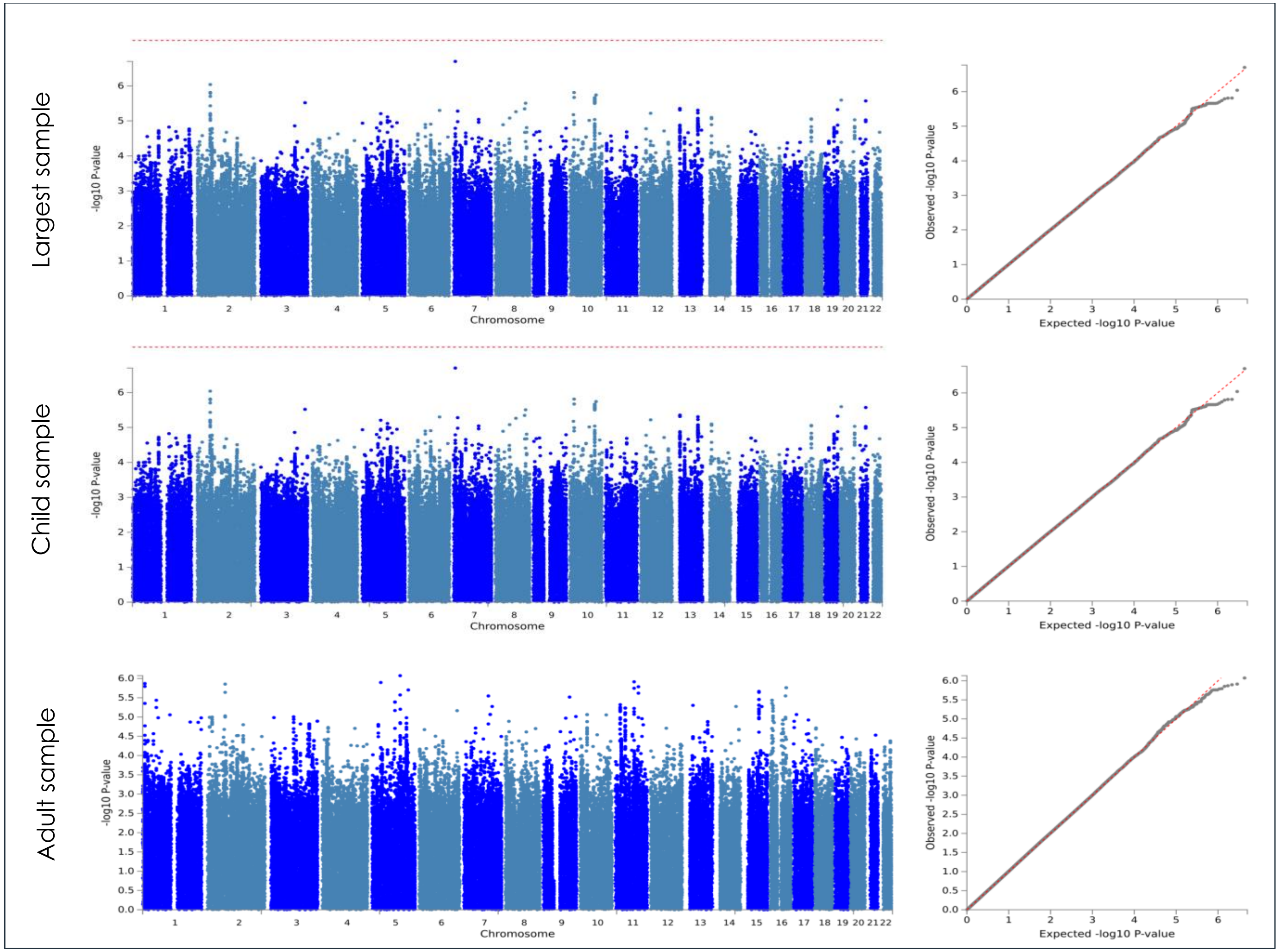

Figure S7. QQ and Manhattan plots of p-values of GWAS meta-analysis ADHD symptoms

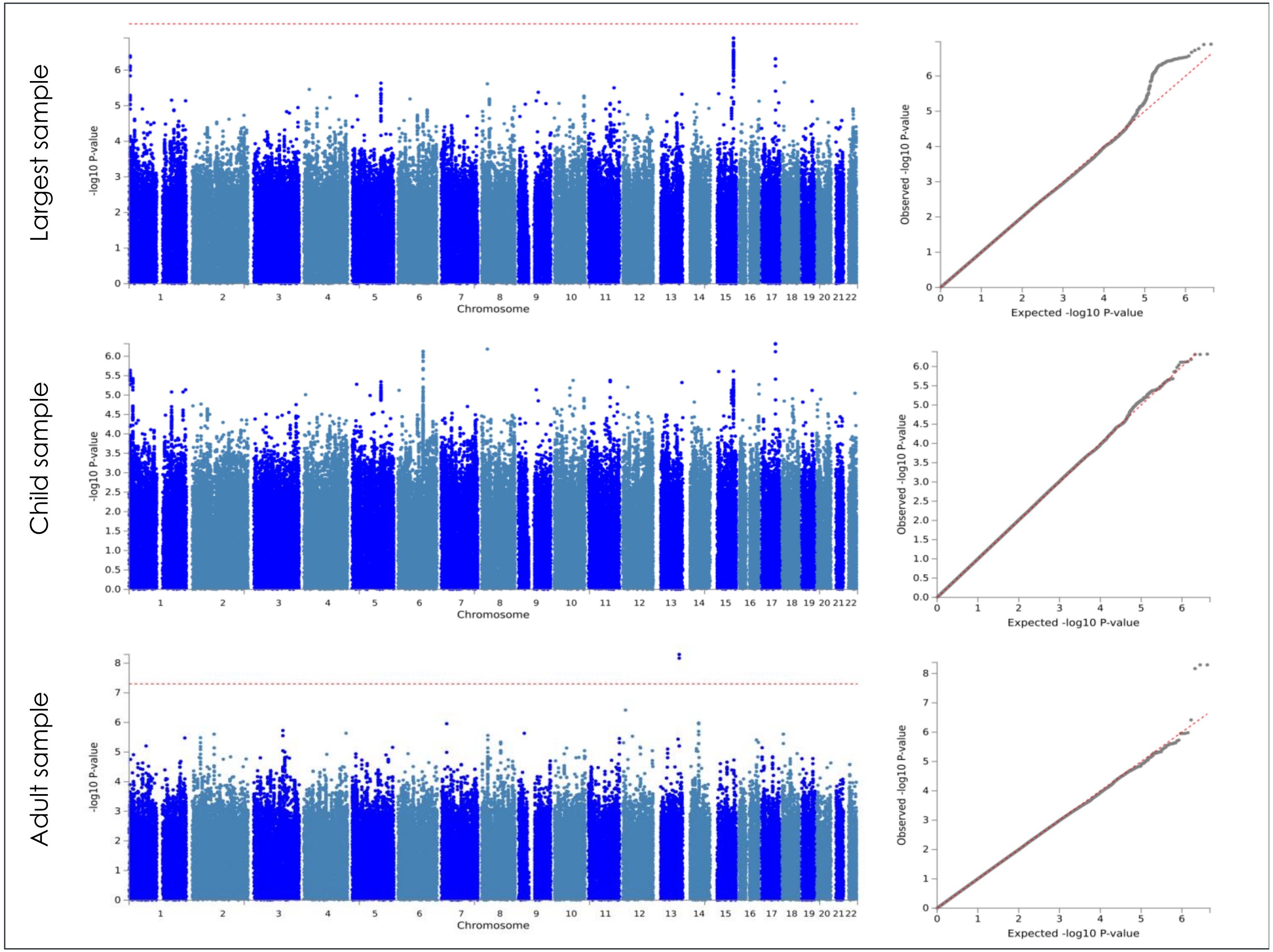

Figure S8. QQ and Manhattan plots of p-values of GWAS meta-analysis - Anxiety symptoms

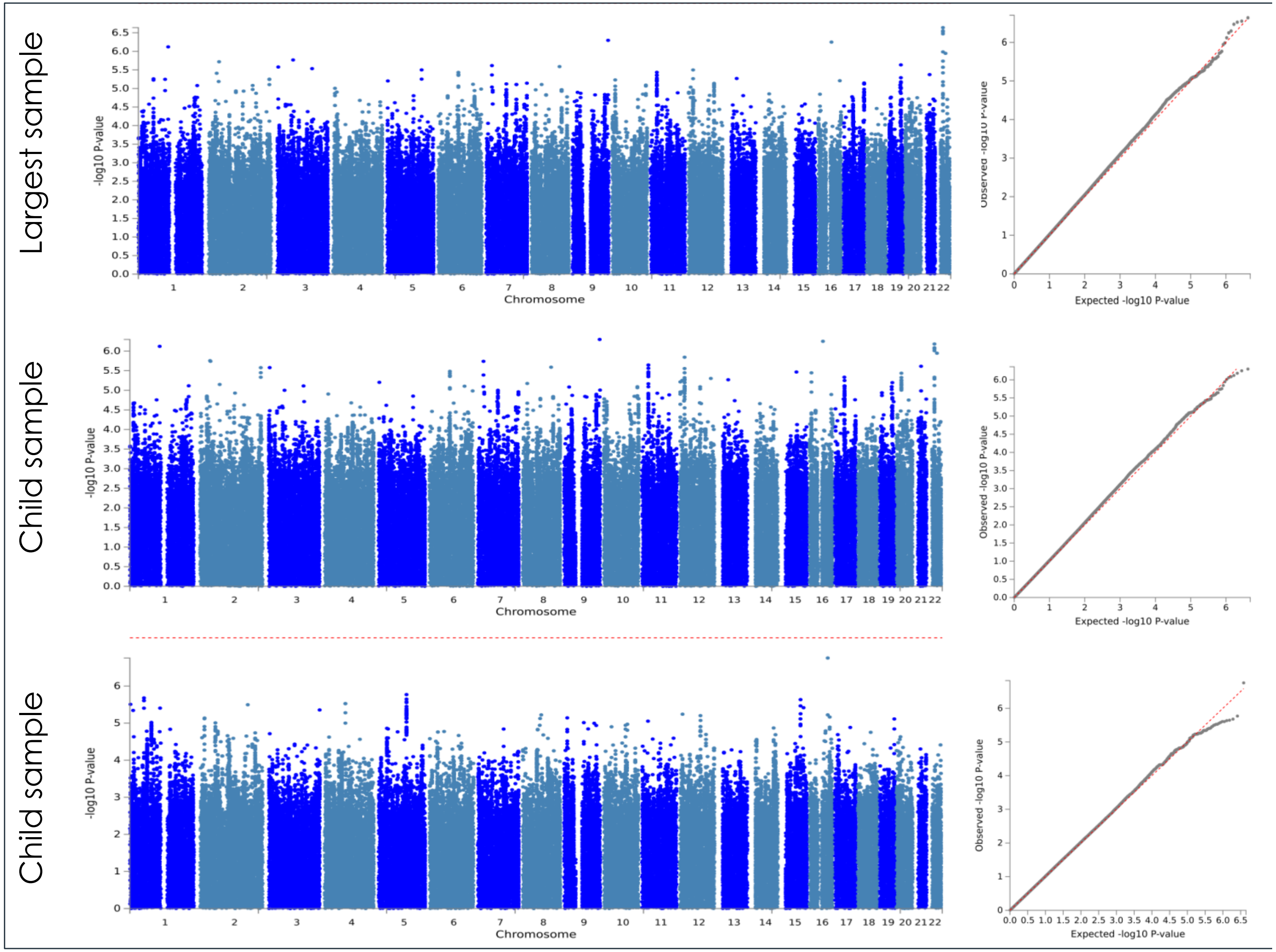

Figure S9. QQ and Manhattan plots of p-values of GWAS meta-analysis- ASD symptoms

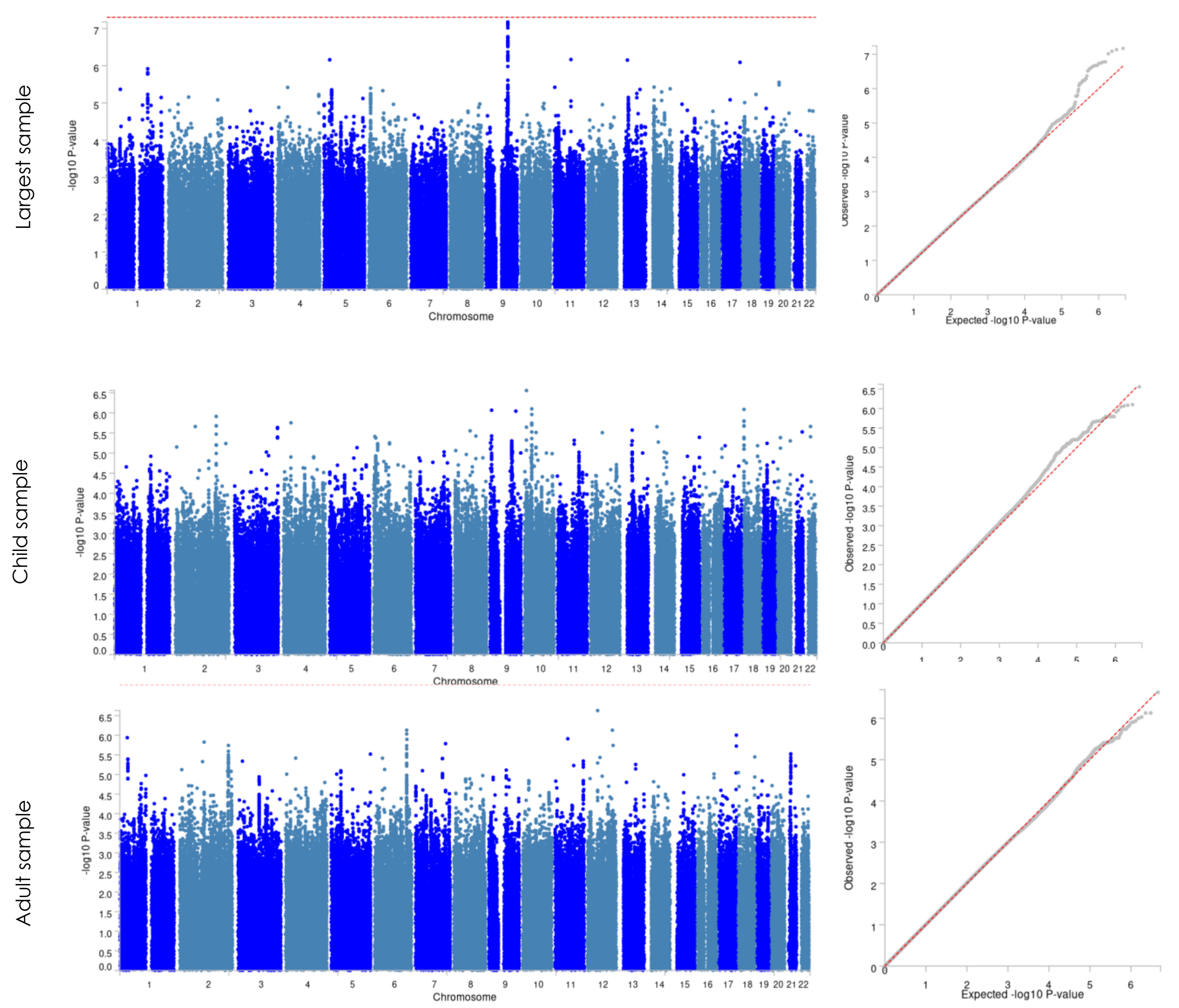

Figure S10. QQ and Manhattan plots of p-values of GWAS meta-analysis- Depression symptoms

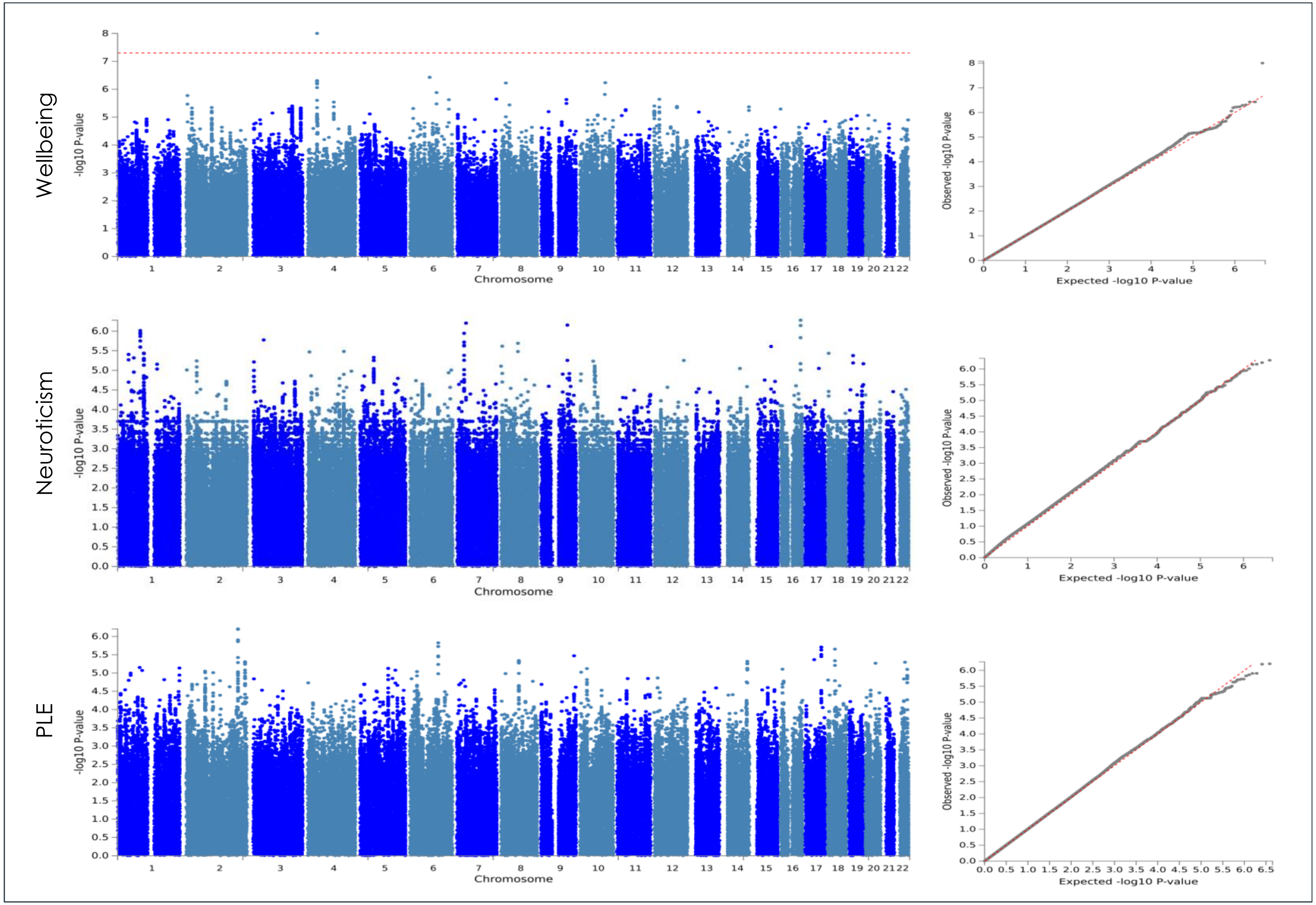

Figure S11. QQ and Manhattan plots of p-values of GWAS meta-analysis (Wellbeing, Neuroticism, PLE)

4. Gene-based and Gene-set analyses

SNP2Gene function from the FUMA web application (v.1.5.2) [13] was used to perform gene-based and gene-set analyses in MAGMA (v1.08)[14]. Please refer to the manual of MAGMA from https://ctg.cncr.nl/software/magma for more details.

GWAS meta-analysis results were filtered for SNPS available in at least 50% of studies to ensure the SNP associations reflected a good coverage across the sample. The following analyses were then conducted in the largest sample per phenotype, and the developmental samples.

Gene-based analysis: GWAS SNPs with p-value <.05 were mapped to 1KG phase 3 European panel, and protein coding genes with unique ensembl ID. All parameters were set as default (SNP-wise (mean) model for gene test).

Gene-set analysis: Gene sets were obtained from MsigDB v7.0. Total 15496 gene sets (Curated gene sets: 5500, GO terms: 9996) were tested.
Curated gene sets consisted of 9 data resources including KEGG, Reactome and BioCarta (http://software.broadinstitute.org/gsea/msigdb/collection_details.jsp#C2 for details).
GO terms consists of three categories, biological processes (bp), cellular components (cc) and molecular functions (mf). All parameters were set as default (competitive test).

Table S5 shows parameters for MAGMA analyses, which were set to be the same for all study phenotypes.

Table S5. MAGMA parameters and descriptions

**SNP2Gene Parameters**<br><br>FUMA = v1.5.2<br>MAGMA = v1.08<br>GWAScatalog = e0_r2022-11-29<br>ANNOVAR = 2017-07-17	[params]<br>exMHC = 1<br>MHCopt = all<br>extMHC = NA<br>ensembl = v92<br>genetype = protein_coding<br>leadP = 1e-5<br>gwasP = 0.05<br>r2 = 0.6<br>r2_2 = 0.1<br>refpanel = 1KG/Phase3<br>pop = EUR<br>MAF = 00<br>refSNPs = 0<br>mergeDist = 250	[magma]<br>magma = 1<br>magma_window = 0<br>magma_exp = 0	[posMap]<br>posMap = 1<br>posMapWindowSize = 10<br>posMapAnnot = NA<br>posMapCADDth = 0<br>posMapRDBth = NA<br>posMapChr15 = NA<br>posMapChr15Max = NA<br>posMapChr15Meth = NA<br>posMapAnnoDs = NA<br>posMapAnnoMeth = NA
N : Sample size of GWAS<br>exMHC : 1 to exclude MHC region, 0 otherwise<br>MHCopt : "annot" to exclude MHC region only from SNP2GENE annotation, "magma" to exclude MHC from MAGMA gene analysis, or "all" to exclude MHC from both.<br>extMHC : user defined MHC region if provided, NA otherwise<br>genetype : All selected gene type.<br>leadP : the maximum threshold of P-value to be lead SNP<br>gwasP : the maximum threshold of P-value to be candidate SNP<br>r2 : the minimum threshold to define independent significant SNPs<br>r2_2 : the minimum threshold to define lead SNPs<br>pop : The population of reference panel<br>MAF : the minimum minor allele frequency based on 1000 genome reference of given population<br>refSNPs : 1 to include non-GWAS-tagged SNPs from reference panel, 0 otherwise<br>mergeDist : The maximum distance between LD blocks to merge into interval<br>magma : 1 to perform MAGMA, 0 otherwise<br>magma_exp : Data sets for magma gene expression analysis.<br>posMap : 1 to perform positional mapping, 0 otherwise<br>posMapWindow : 1 to perform positional mapping based on distance to the genes, 0 otherwise<br>posMapWindowSize : If window based positional mapping is performed, which distance (kb) as the maximum. If window-based mapping is 0, this parameter set at 10 as default but will be ignored.<br>posMapAnnot : Positional annotations selected if window based mapping is 0.			

4.1. MAGMA gene-based analyses results

Full gene-based results per phenotype are in files with extension "phenotype_magma_genes.out".

Figures S12 -S17: gene-based Manhattan and QQ plots

Table S6 shows the top genes per phenotype.

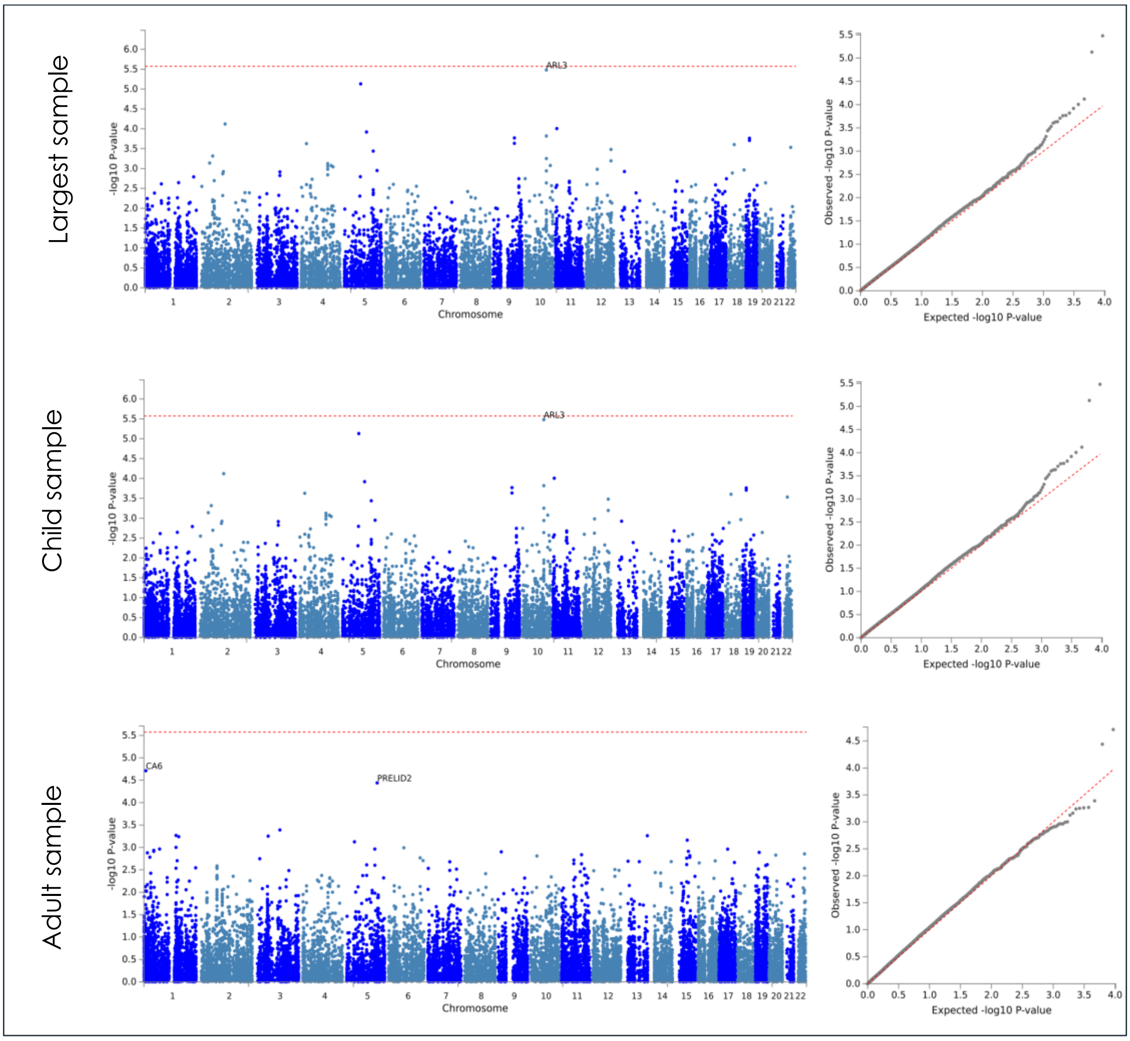

Figure S12. QQ and Manhattan plots of gene-based results for ADHD symptoms

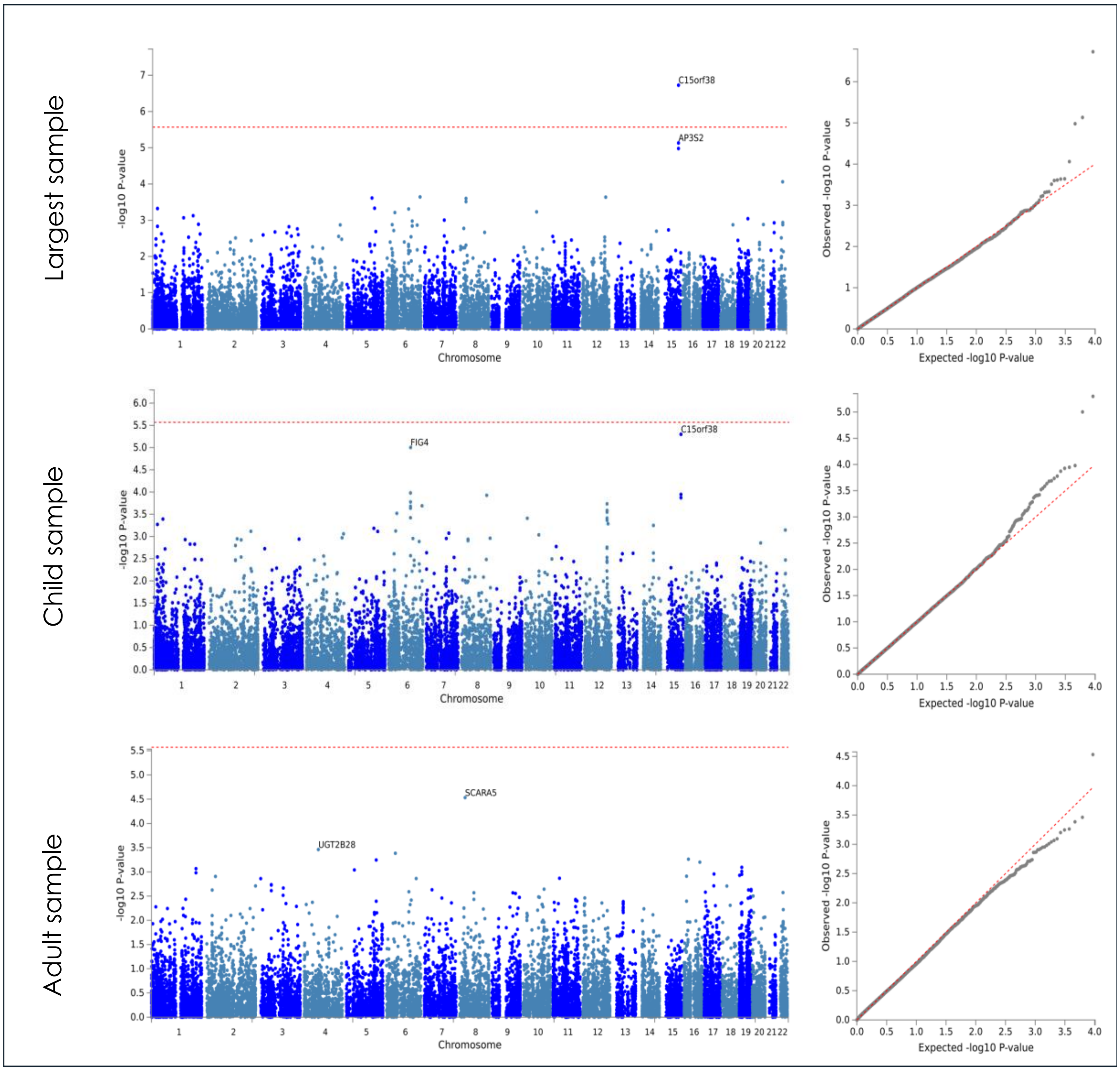

Figure S13. QQ and Manhattan plots of gene-based results for anxiety symptoms

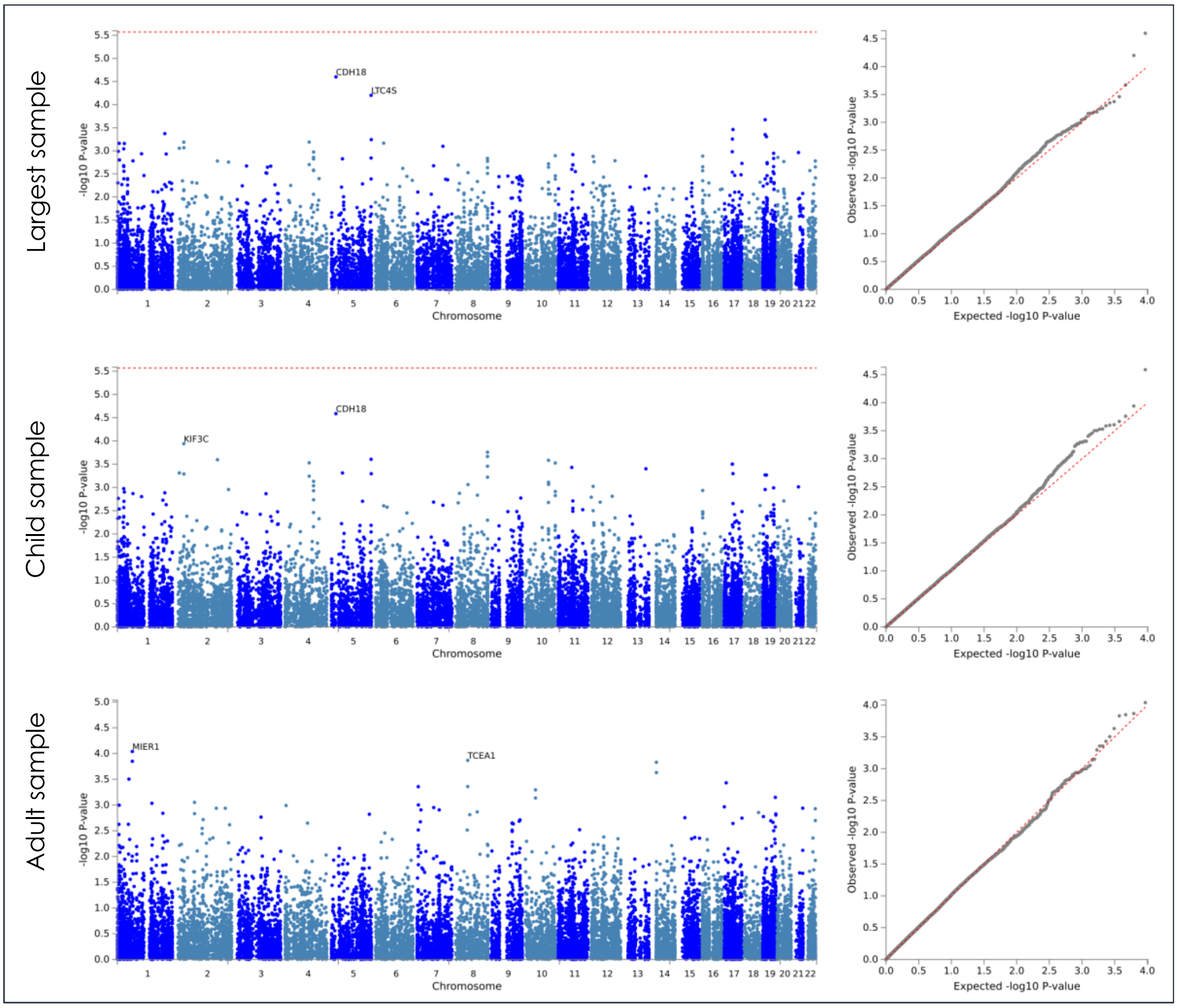

Figure S14. QQ and Manhattan plots of gene-based results for ASD symptoms

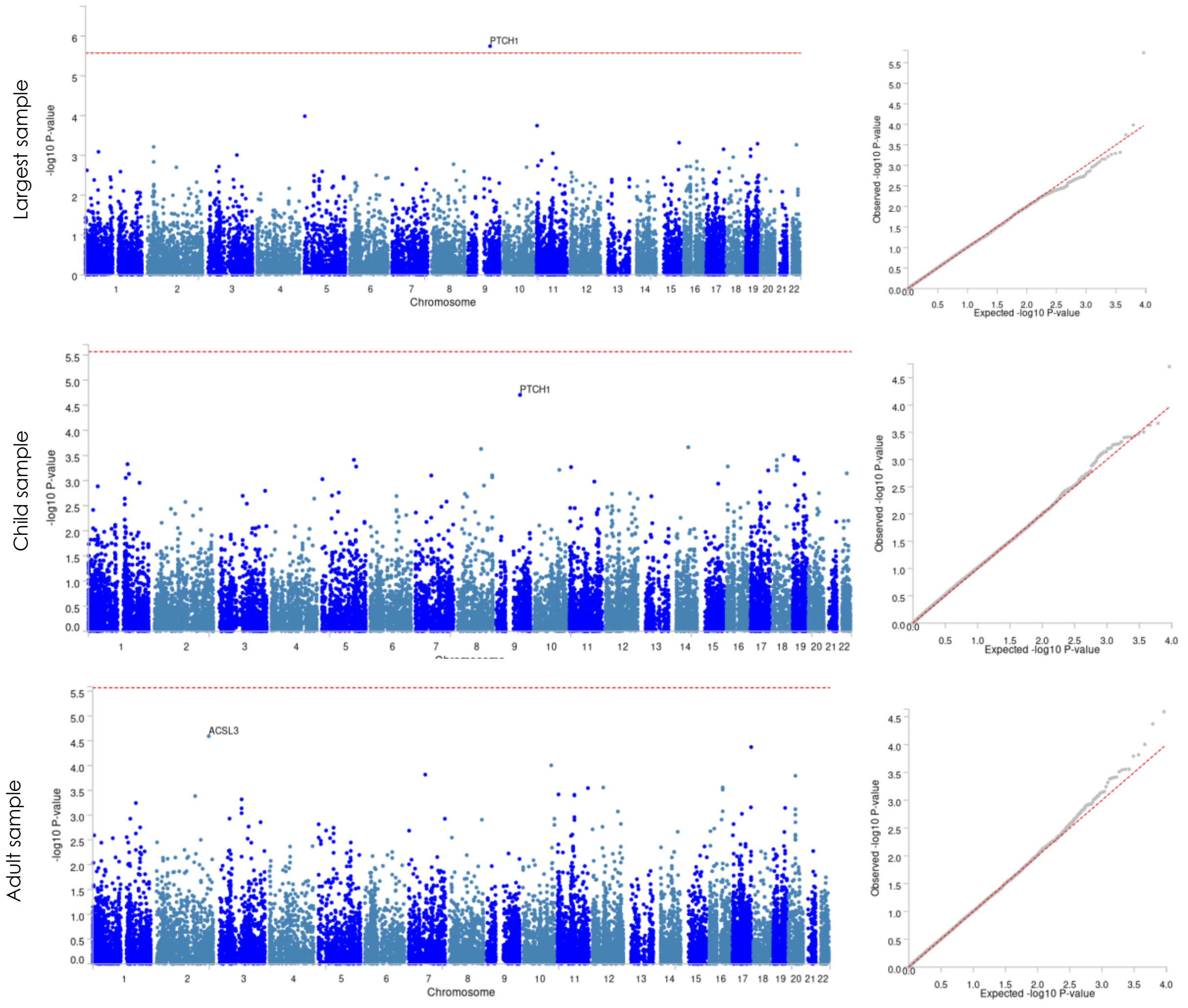

Figure S15. QQ and Manhattan plots of gene-based results for depression symptoms

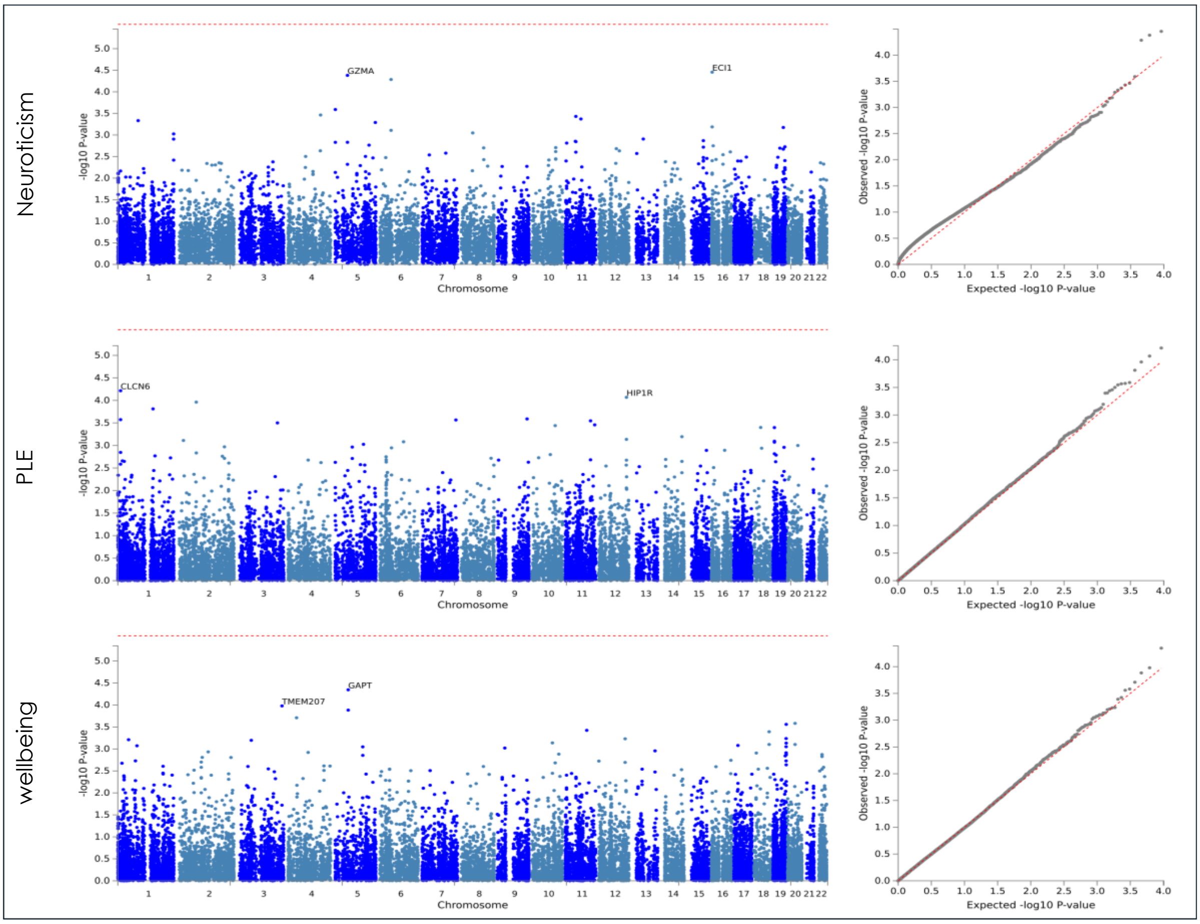

Figure S16. QQ and Manhattan plots of gene-based results for neuroticism, PLE and wellbeing

Table S6. Top genes per phenotype and across developmental groups

Phenotype	Sample	Gene	Chr	NSNPS	NPARAM	N	ZSTAT	P
ADHD	Largest*	*ARL3*	10	63	11	6675	4.51	3.00E-06
	Child	*ARL3*	10	63	11	6675	4.51	3.00E-06
	Adolescent	*CA6*	1	120	25	3739	4.11	2.00E-05
Anxiety	Largest	***C15orf38***	**15**	**28**	**7**	**5265**	**5.08**	**2.00E-07**
	Child	***C15orf38***	**15**	**29**	**8**	**4553**	**4.42**	**5.00E-06**
	Adult	*SCARA5*	8	533	46	3625	4.02	3.00E-05
autistic traits	Largest	*CDH18*	5	2583	87	6607	4.05	3.00E-05
	Child	*CDH18*	5	2497	85	6363	4.05	3.00E-05
	Adult	*MIER1*	1	133	12	2869	3.74	9.00E-05
Depression	**Largest**	***PTCH1***	**9**	**130**	**12**	**10166**	**4.63**	**1.80E-06**
	Child	*PTCH1*	9	145	15	5116	4.11	1.97E-05
	Adult	*ACSL3*	2	215	17	8796	4.05	2.56E-05
Neuroticism	Adult	*ECI1*	16	9	2	2653	3.98	4.00E-05
Wellbeing	Adult	*GAPT*	5	6	2	6859	3.92	5.00E-05
PLE	Adolescent	*CLCN6*	1	173	12	1817	3.84	6.00E-05

Notes: Largest: largest available sample, obtained by selecting the largest sample from each study, irrespective of age group. Child: data from studies where participants were aged 5-12 years old; Adolescent: data from studies where participants were aged 13-18 years old; Adult: data from studies where participants were aged >18 years old. * same sample as the child sample. Results with significant p-values after Bonferroni correction are highlighted in bold.

4.2. MAGMA gene-set analyses results

Full gene-based results per phenotype are in files with extension "phenotype_magma.gsa.out". **Table S7** shows top gene-sets per phenotype.

Details of statistically significant gene-sets that were robust to Bonferroni correction, are presented in **Tables S8-S11**. **Table S12** shows overview of all annotated genome-wide significant results per phenotype.

Table S7. Top gene-sets per phenotype and across developmental groups

Pheno	Sample	Gene Set	N genes	Beta	SD	SE	P	Pbon
ADHD	Largest	Curated_gene_sets:reactome_crmps_in_sema3a_signaling	15	0.8	0.02	0.21	6.00E-05	0.9
	Child only	Curated_gene_sets:reactome_crmps_in_sema3a_signaling	15	0.8	0.02	0.21	6.00E-05	0.9
	Adolescent	GO_bp:go_nonassociative_learning	6	1.45	0.03	0.37	5.00E-05	0.8
Anxiety	Largest	Curated_gene_sets:reactome_cell_extracellular_matrix_interactions	16	0.7	0.02	0.2	2.00E-04	1
	Child	Curated_gene_sets:abdelmohsen_elavl4_targets	16	0.8	0.02	0.2	4.00E-05	0.67
	Adult	Curated_gene_sets:biocarta_ppara_pathway	49	0.44	0.02	0.11	4.00E-05	0.64
autistic traits	Largest	Curated_gene_sets:plasari_tgfb1_targets_1hr_dn	5	1.29	0.02	0.34	8.00E-05	1
	Child	**Curated_gene_sets:plasari_tgfb1_targets_1hr_dn**	**5**	**1.68**	**0.03**	**0.34**	**6.00E-07**	**0.01**
	Adult	**GO_bp:go_regulation_of_protein_localization_to_cilium**	**7**	**1.43**	**0.03**	**0.26**	**1.00E-08**	**0.0002**
Dep	Largest	GOCC_TRANSCRIPTION_REPRESSOR_COMPLEX	72	0.36	0.02	0.10	1.07E-04	1
	Child	**GOCC_PROTEASOME_REGULATORY_PARTICLE**	**19**	**0.79**	**0.03**	**0.17**	**1.41E-06**	**0.02**
	Adult	Curated_gene_sets:holleman_vincristine_resistance_all_up	26	0.70	0.03	0.18	3.78E-05	0.64
Neuro	Adult	**GO_cc:go_gemini_of_coiled_bodies**	**9**	**1.01**	**0.02**	**0.2**	**2.00E-07**	**0.003**
Well	Adult	GO_bp:go_bone_resorption	58	0.41	0.02	0.11	1.00E-04	1
PLE	Adolescent	**GO_bp:go_regulation_of_dopamine_uptake_involved_in_synaptic_transmission**	**8**	**1.37**	**0.03**	**0.28**	**5.00E-07**	**0.01**

Notes: Largest: largest available sample, obtained by selecting the largest sample from each study, irrespective of age group. Child: data from studies where participants were aged 5-12 years old; Adolescent: data from studies where participants were aged 13-18 years old; Adult: data from studies where participants were aged >18 years old. Results with significant p-values after Bonferroni correction are highlighted in bold.

Tables S8 – S11: Genome-wide significant gene-sets details

Table S8. statistically significant gene-sets after Bonferroni correction (ASD symptoms)

GENE	CHR	START	STOP	NSNPS	NPARAM	N	ZSTAT	P	ZFITTED_BASE	ZRESID_BASE
Child sample (Curated_gene_sets: plasari_tgfb1_targets_1hr_dn (p= 5.6E-07))										
ENSG00000117289	1	145438469	145442635	4	1	6563	1	0.22	6.0E-15	0.74
ENSG00000168542	2	189839046	189877472	78	11	6366	0	0.36	5.9E-15	0.34
ENSG00000147862	9	14081842	14398982	612	89	6241	0	0.49	6.0E-15	-0.10
ENSG00000119938	10	93388199	93392811	6	4	5967	1	0.18	6.0E-15	0.91
ENSG00000134138	15	37181406	37393504	417	63	6365	1	0.09	6.0E-15	1.23
Adult sample (GO_bp:go_regulation_of_protein_localization_to_cilium (p-value= 1.39E-08))										
ENSG00000058453	1	17066768	17299474	135	12	2621	2	0.02	9.8E-15	2.13
ENSG00000203965	1	63989043	64038364	110	11	2916	2	0.05	9.8E-15	1.60
ENSG00000115355	2	55514978	55647057	400	19	2882	-1	0.86	9.8E-15	-1.15
ENSG00000163818	3	45864808	45957534	173	25	2942	0	0.40	9.9E-15	0.22
ENSG00000180376	3	56591189	56655846	257	17	2936	0	0.38	9.9E-15	0.27
ENSG00000165689	9	139296377	139305061	46	9	2899	0	0.31	9.9E-15	0.48
ENSG00000141013	16	90086037	90111383	117	16	2878	1	0.26	9.9E-15	0.62

N gene sets = 15480

Table S9. statistically significant gene-set after Bonferroni correction (depression symptoms - child sample)

GENE	CHR	START	STOP	NSNPS	NPARAM	N	ZSTAT	P	ZFITTED_BASE	ZRESID_BASE
GOCC_PROTEASOME_REGULATORY_PARTICLE (p=1.41e-06)										
ENSG00000159352	1	151227179	151239955	15	3	4812	1	0.21	1.3E-14	0.78
ENSG00000115233	2	162164549	162268228	143	15	5109	1	0.13	1.3E-14	1.09
ENSG00000173692	2	231921578	232037541	171	17	5103	1	0.10	1.3E-14	1.24
ENSG00000163636	3	63996225	64009658	30	7	5194	2	0.02	1.2E-14	2.05
ENSG00000175166	3	184016497	184026842	21	6	4920	1	0.26	1.3E-14	0.59
ENSG00000161057	7	102984701	103009842	38	7	5015	0	0.45	1.3E-14	0.08
ENSG00000185627	11	236546	252983	93	12	5051	1	0.12	1.3E-14	1.14
ENSG00000165916	11	47440320	47447993	13	4	4733	1	0.20	1.3E-14	0.80
ENSG00000110801	12	122326637	122356203	64	9	5168	2	0.06	1.3E-14	1.51
ENSG00000100519	14	53173890	53195305	26	6	5078	-1	0.75	1.3E-14	-0.69
ENSG00000100764	14	90722839	90738968	50	2	5253	1	0.19	1.3E-14	0.87
ENSG00000103035	16	74330673	74340186	12	6	4835	1	0.22	1.3E-14	0.73
ENSG00000108671	17	30771279	30810336	35	7	5202	0	0.50	1.3E-14	-0.04
ENSG00000108344	17	38137050	38154213	55	6	4849	0	0.36	1.3E-14	0.35
ENSG00000087191	17	61904512	61909379	8	2	5254	1	0.15	1.3E-14	1.02
ENSG00000197170	17	65334032	65362743	46	10	5085	3	0.00	1.2E-14	2.67
ENSG00000099341	19	38865176	38874464	24	5	5190	-1	0.88	1.3E-14	-1.19
ENSG00000013275	19	40476912	40487348	22	4	5026	0	0.51	1.3E-14	-0.06
ENSG00000130706	20	60877149	60883918	15	4	5251	0	0.53	1.3E-14	-0.12
WP_PROTEASOME_DEGRADATION (p-value=2.48e-06)										
ENSG00000126067	1	36067185	36107445	15	3	5052	-1	0.72	1.27E-14	-0.63
ENSG00000143106	1	109941653	109969062	36	5	4974	-1	0.79	1.27E-14	-0.82
ENSG00000159352	1	151227179	151239955	15	3	4812	1	0.21	1.27E-14	0.78
ENSG00000159377	1	151372010	151374420	2	1	5254	2	0.05	1.27E-14	1.59
ENSG00000173692	2	231921578	232037541	171	17	5103	1	0.10	1.27E-14	1.24
ENSG00000182179	3	49842640	49851379	7	2	5004	1	0.20	1.27E-14	0.83
ENSG00000163636	3	63996225	64009658	30	7	5194	2	0.02	1.24E-14	2.05
ENSG00000163902	3	128338817	128399918	166	16	5182	-1	0.89	1.27E-14	-1.28
ENSG00000175166	3	184016497	184026842	21	6	4920	1	0.26	1.27E-14	0.59
ENSG00000154277	4	41258430	41270472	43	9	5112	1	0.19	1.27E-14	0.85
ENSG00000164032	4	100869243	100871545	6	2	5002	1	0.29	1.27E-14	0.51
ENSG00000109332	4	103715540	103790053	144	7	5083	2	0.06	1.27E-14	1.53
ENSG00000119048	5	133706870	133727683	27	2	5124	0	0.64	1.27E-14	-0.39
ENSG00000131508	5	138906016	139008018	120	13	4842	2	0.05	1.27E-14	1.56
ENSG00000008018	6	170844205	170862429	60	7	4857	0	0.56	1.26E-14	-0.16
ENSG00000106588	7	42956460	42971822	28	3	5146	0	0.36	1.27E-14	0.31
ENSG00000161057	7	102984701	103009842	38	7	5015	0	0.45	1.26E-14	0.08
ENSG00000095261	9	123577774	123605262	32	6	5196	0	0.50	1.26E-14	-0.04
ENSG00000136930	9	127115745	127177723	88	11	4805	1	0.22	1.27E-14	0.75
ENSG00000072401	10	60094735	60130513	66	11	5147	1	0.27	1.27E-14	0.58
ENSG00000185627	11	236546	252983	93	12	5051	1	0.12	1.27E-14	1.14
ENSG00000129084	11	14515329	14665181	177	25	4938	0	0.40	1.26E-14	0.20
ENSG00000165916	11	47440320	47447993	13	4	4733	1	0.20	1.27E-14	0.80
ENSG00000188486	11	118964564	118966177	4	1	5252	2	0.03	1.27E-14	1.91
ENSG00000111537	12	68548548	68553527	5	2	4951	0	0.33	1.27E-14	0.41
ENSG00000110801	12	122326637	122356203	64	9	5168	2	0.06	1.27E-14	1.51
ENSG00000150991	12	125396150	125401914	9	3	5252	1	0.13	1.27E-14	1.07
ENSG00000118939	13	76123619	76180085	154	14	5049	-1	0.81	1.27E-14	-0.90
ENSG00000100804	14	23485752	23504439	23	5	4703	0	0.40	1.26E-14	0.21
ENSG00000092010	14	24605367	24608176	4	1	4875	1	0.11	1.27E-14	1.20
ENSG00000100911	14	24612574	24616779	6	3	4669	2	0.03	1.27E-14	1.77
ENSG00000100902	14	35747839	35786699	113	13	4946	-1	0.72	1.27E-14	-0.63
ENSG00000100519	14	53173890	53195305	26	6	5078	-1	0.75	1.27E-14	-0.69
ENSG00000100567	14	58711549	58738730	45	8	4973	0	0.45	1.26E-14	0.07
ENSG00000100764	14	90722839	90738968	50	2	5253	1	0.19	1.27E-14	0.87
ENSG00000069869	15	56119120	56285944	620	23	5211	0	0.52	1.26E-14	-0.10
ENSG00000041357	15	78832747	78841604	26	4	4868	0	0.61	1.27E-14	-0.29
ENSG00000205220	16	67968405	67970990	2	1	5254	1	0.16	1.27E-14	0.93
ENSG00000103035	16	74330673	74340186	12	6	4835	1	0.22	1.27E-14	0.73
ENSG00000142507	17	4699439	4701790	3	1	4749	0	0.61	1.27E-14	-0.35
ENSG00000170315	17	16284112	16286059	2	1	5254	0	0.58	1.27E-14	-0.26
ENSG00000108671	17	30771279	30810336	35	7	5202	0	0.50	1.26E-14	-0.04
ENSG00000108344	17	38137050	38154213	55	6	4849	0	0.36	1.27E-14	0.35
ENSG00000131467	17	40976402	40995774	4	2	4814	-1	0.89	1.27E-14	-1.31
ENSG00000087191	17	61904512	61909379	8	2	5254	1	0.15	1.27E-14	1.02
ENSG00000197170	17	65334032	65362743	46	10	5085	3	0.00	1.24E-14	2.67
ENSG00000099341	19	38865176	38874464	24	5	5190	-1	0.88	1.27E-14	-1.19
ENSG00000013275	19	40476912	40487348	22	4	5026	0	0.51	1.26E-14	-0.06
ENSG00000118705	20	35806813	35870022	190	11	5157	1	0.09	1.27E-14	1.28
ENSG00000101182	20	60711791	60718496	15	2	5252	2	0.04	1.27E-14	1.76

Table S10. statistically significant gene-set after Bonferroni correction (neuroticism)

GENE	CHR	START	STOP	NSNPS	NPARAM	N	ZSTAT	P	ZFITTED_BASE	ZRESID_BASE
GO_bp:go_mrna_3_end_processing_by_stem_loop_binding_and_cleavage (p=2.18E-06)										
ENSG00000119203	2	9563697	9613230	60	4	4354.00	1	3.7E-02	-1.8E-15	1.42
ENSG00000163950	4	1694527	1714282	39	7	3835.00	2	2.2E-02	-1.8E-15	1.63
ENSG00000155858	5	157170703	157187717	33	5	3488.00	0	3.2E-01	-1.7E-15	0.10
ENSG00000165934	14	92588281	92630755	92	8	4033.00	1	1.4E-01	-1.7E-15	0.69
ENSG00000142528	19	50528971	50556671	41	8	3392.00	0	3.6E-01	-1.7E-15	-0.02
GO_cc:go_gemini_of_coiled_bodies (p=2.22E-07)										
ENSG00000064703	1	112297867	112310638	39	5	4288.00	1	5.8E-02	-1.8E-15	1.21
ENSG00000152147	2	38978676	39012142	58	6	4010.00	0	2.3E-01	-1.7E-15	0.37
ENSG00000082516	5	154266976	154317769	106	11	3688.00	0	4.1E-01	-1.7E-15	-0.19
ENSG00000130956	9	99212483	99253618	60	5	4166.00	0	4.7E-01	-1.7E-15	-0.30
ENSG00000149308	11	108027942	108093369	70	6	3410.00	1	1.5E-01	-1.7E-15	0.65
ENSG00000109917	11	116648436	116658766	23	6	4196.00	0	3.8E-01	-1.7E-15	-0.05
ENSG00000092208	14	39583427	39606177	58	11	4188.00	1	1.2E-01	-1.7E-15	0.77
ENSG00000179409	17	647654	657239	27	5	3533.00	1	4.4E-02	-1.8E-15	1.34
ENSG00000142252	19	45582453	45594782	51	4	2715.00	1	1.9E-01	-1.7E-15	0.53

N gene-sets= 15478

Table S11. statistically significant gene-set after Bonferroni correction (PLE)

GENE	CHR	START	STOP	NSNPS	NPARAM	N	ZSTAT	P	ZFITTED_BASE	ZRESID_BASE
GO_bp:go_regulation_of_dopamine_uptake_involved_in_synaptic_transmission (p=5.01E-07)										
GENE	CHR	START	STOP	NSNPS	NPARAM	N	ZSTAT	P	ZFITTED_BASE	ZRESID_BASE
ENSG00000169213	1	52373628	52456436	148	17	1817	0.01	4.9E-01	5.01E-15	0.01
ENSG00000151577	3	113847499	113918254	188	24	1817	1.40	7.9E-02	5.11E-15	1.40
ENSG00000145335	4	90645250	90759466	282	15	1817	0.76	2.1E-01	5.00E-15	0.76
ENSG00000168621	5	37812779	37839788	68	13	1817	-0.01	5.0E-01	5.01E-15	-0.01
ENSG00000184845	5	174867042	174871211	6	2	1817	0.04	4.8E-01	5.01E-15	0.04
ENSG00000136827	9	132575223	132586413	22	5	1817	2.09	1.8E-02	4.89E-15	2.09
ENSG00000069696	11	637293	640706	5	3	1817	0.90	1.9E-01	5.00E-15	0.90
ENSG00000149295	11	113280318	113346413	163	21	1817	0.49	3.0E-01	5.00E-15	0.49
GO_bp:go_catecholamine_uptake_involved_in_synaptic_transmission (p=9.55E-07)										
ENSG00000116288	1	8014351	8045565	56	8	1817	-0.92	8.2E-01	5.00E-15	-0.92
ENSG00000169213	1	52373628	52456436	148	17	1817	0.01	4.9E-01	5.01E-15	0.01
ENSG00000151577	3	113847499	113918254	188	24	1817	1.40	7.9E-02	5.11E-15	1.40
ENSG00000145335	4	90645250	90759466	282	15	1817	0.76	2.1E-01	5.00E-15	0.76
ENSG00000168621	5	37812779	37839788	68	13	1817	-0.01	5.0E-01	5.01E-15	-0.01
ENSG00000184845	5	174867042	174871211	6	2	1817	0.04	4.8E-01	5.01E-15	0.04
ENSG00000185345	6	161768452	163148803	4275	216	1817	0.66	2.4E-01	5.00E-15	0.66
ENSG00000136827	9	132575223	132586413	22	5	1817	2.09	1.8E-02	4.89E-15	2.09
ENSG00000069696	11	637293	640706	5	3	1817	0.90	1.9E-01	5.00E-15	0.90
ENSG00000149295	11	113280318	113346413	163	21	1817	0.49	3.0E-01	5.00E-15	0.49
ENSG00000103546	16	55689516	55740104	151	27	1817	0.38	3.4E-01	5.00E-15	0.38
GO_cc:go_extrinsic_component_of_endoplasmic_reticulum_membrane (p=1.59E-06)										
ENSG00000163874	1	37940153	37949980	12	5	1817	-1.42	9.2E-01	5.11E-15	-1.42
ENSG00000136710	2	131095814	131099922	3	1	1817	-0.08	5.3E-01	5.01E-15	-0.08
ENSG00000136827	9	132575223	132586413	22	5	1817	2.09	1.8E-02	4.89E-15	2.09
ENSG00000137675	11	102562218	102576537	71	11	1817	0.69	2.4E-01	5.00E-15	0.69
ENSG00000140464	15	74287014	74340153	127	13	1817	-0.34	6.2E-01	5.00E-15	-0.34

Table S12: Genome-wide significant results per phenotype

Phenotype-sample	analysis type	variant	Details
Anxiety - child	Gene-based analysis	*C15orf38*	Chromosome 15 open reading frame 38 (C15orf38) gene (also known as ARPIN-AP3S2) is a protein-coding gene involved in steerig cell migration by controlling its directional persistence. SNPs in this gene have also been consistently associated with type 2 diabetes in adults [15,16], and also with corticotropin-releasing factor protein (CRFBP) levels [17]. As well as regulating the stress response, the CRF peptides are though to regulate anxiety, mood, eating, and inflammation[6]. CRFBP are considered as drug targets for stress-related illness (Curley et al., 2021). Levels of insulin interact with environmental factors such as dietary input and stress[7] and hypoglycaemia symptoms in Type2 diabetes include rapid heartbeat, sweating, and nervousness. These symptoms also feature strongly as physical sensations associated with anxiety. It is possible that certain variants in this gene impact sensitivity to the effects of environmental factors such as diet and stressors that are involved in variability in insulin and corticotropin levels; unpleasant physical sensations related to insulin and corticotropin levels may be contextualised and made sense of as worries and anxieties, especially in those individuals who are more attuned to detecting these physical sensations (two factor model of emotions[18]). This gene may therefore relate to variability in anxiety symptoms brought about by variability in biophysiological systems relating to type 2 diabetes and stress response.
Anxiety- adult	GWAS meta-analysis	rs60358762	The SNP has not previously been associated with phenotypes in GWAS catalogue. The SNP is located in the intergenic region of the protein-coding s olute carrier family 15-member 1 *(SLC15A1)* gene on chromosome 13. NCBI Gene Summary: "This gene encodes an intestinal hydrogen peptide cotransporter, a member of the solute carrier family 15. The encoded protein is localized to the brush border membrane of the intestinal epithelium and mediates the uptake of di- and tripeptides from the lumen into the enterocytes and plays an important role in the uptake and digestion of dietary proteins and facilitates the absorption of peptidomimetic drugs. " Transport of glucose and other sugars, bile salts and acids are amongst its pathways. It is possible that variations in transport, uptake and digestion of dietary inputs relate to symptoms of anxiety in a similar way as above.
Autistic traits - child	gene-set analysis	Curated_gene_sets:plasari_tgfb1_targets_1hr_dn	The genes in this gene set are involved in tissue morphogenesis and repair. Genes in this gene set were down regulated in nuclear factor I C (NFIC) knock out vs wild type mice, after 1 h of transforming growth factor beta (TGFβ-1) stimulation in MEF cells. They play a central role in the interplay of other pathways involved in tissue morphosis and repair, and in regulation of the progression of tissue regeneration. There is evidence that autism is associated with other growth factor genes such as Epidermal growth factor (EGF), TGFβ-1 and hepatocyte growth factor (HGF) and reduced sera levels are associated with autism [19,20,21]. Growth factors serve important roles in neurodevelopment, immune function, and development of the central nervous system (CNS). These suggest growth factor dysregulation may contribute to ASD aetiology by affecting CNS development, and/or by influencing immune function.
Autistic traits- Adult		GO_bp:go_regulation_of_protein_localization_to_cilium	This gene set includes any process that modulates the frequency, rate or extent of protein localization to cilium. Cilia serve an important role in signalling pathways involved in early development including cell and tissue differentiation, and also involved in translating external stimulations into neuronal signals that regulate the activity of the cells in which they are localised [22]; For example, in the nose they detect odours and signal olfactory neurons, and in the eye, the photoreceptors response to light. There is no previous report of this gene-set being associated with ASD, however, hypersensitivity to sensory stimuli is a feature of ASD and may relate to variations in these genes.
Depression - Largest	Gene-based analysis	*PTCH1*	This is a protein-coding gene, and SNPs in this gene are associated with numerous phenotypes in the GWAS catalogue, most notably for depression-related phenotypes, including neuroticism [15,23], anxiety[24,25], depression symptoms[23], feeling emotionally hurt[26] and sensitivity to environmental stress and adversity[26]
Depression- child	gene-set analysis	GOCC_PROTEASOME_REGULATORY_PARTICLE	The proteasome terminates the existence of thousands of short-lived, damaged, or misfolded proteins. The genes in this gene-set are involved in proteasome regulatory particle, a multi-subunit complex that is involved in degradation of proteins by recognizing and processing polyubiquitinated substrates [27].
		WP_PROTEASOME_DEGRADATION	The proteasome terminates the existence many short-lived, damaged, or misfolded proteins. The decline in proteasomal activity is associated with aging, cancer, neurodegenerative diseases, and other late-onset diseases.[28]
Neuroticism	gene-set analysis	GO_cc:go_gemini_of_coiled_bodies;	Gemini of coiled bodies, or 'gems', do not contain small nuclear ribonucleoproteins (snRNPs), instead they contain a protein called survivor of motor neurons (SMN) whose function relates to snRNP biogenesis and play a role in the aetiology of spinal muscular atrophy (PMID:11031238, PMID:9683623).
		GO-_bp:go_mrna_end_processing_by_stem_loop_binding_and_cleavage	Any mRNA 3'-end processing that involves the binding to and cleavage of a stem-loop structure. The genes in this gene set are from the transcription factor gene family.
Psychotic-like experiences	gene-set analysis	GO_bp:go_regulation_of_dopamine_uptake_involved_in_synaptic_transmission	This gene set relates to regulation of dopamine uptake involved in synaptic transmission. This is significant association because, the dopamine hypothesis in schizophrenia suggests dysregulation of dopaminergic systems is involved in patho-aetiology of psychosis [5]
		GO_bp:go_catecholamine_uptake_involved_in_synaptic_transmission	This gene set relates to regulation of catecholamine uptake involved in synaptic transmission. This is significant association because, the dopamine hypothesis in schizophrenia suggests dysregulation of dopaminergic systems is involved in patho-aetiology of psychosis [29]
		GO_cc:go_extrinsic_component_of_endoplasmic_reticulum_membrane	endoplasmic reticulum membrane is the lipid bilayer surrounding the endoplasmic reticulum. The endoplasmic reticulum is a large strucure that serves many roles in the cell including calcium storage, protein synthesis and lipid metabolism 30
Wellbeing	GWAS meta-analysis	rs2940988	rs2940988 is located in the intronic region of the protein-coding, uncharacterised chromosome 4 open reading frame 19 (*C4orf19)* gene. The SNP is not associated with any phenotypes in the GWAS catalogue, but the gene is associated with cardiometabolic health, adolescent idiopathic scoliosis and serum gamma-glutamyl transferase [31], a marker of alcohol abuse.

5. Heritability analyses

The heritability estimates as shown in Table S13 were largely negative or low with large standard errors. We examined several possibilities to test whether the estimates could be improved. One potential reason for low SNP-heritability is the over-correction for confounding due to population stratification. Therefore, we ran our analyses using results from Model 3 which did not correct for population stratification in the phenotype. This did not make a significant difference to the results.
We also examined the heterogeneity of SNP effects across studies in meta-analysis results and removed those SNPS that showed significant heterogeneity across samples. The estimates were not improved. We also ran our analyses using a more stringent imputation quality score (INFO >.9), but the estimates did not improve.

Table S13. SNP-heritability estimates per phenotype

Phenotype	Sample	h2	Lambda GC	Mean Chi^2	Intercept	Ratio
ADHD	Largest	.04 (.06)	1.008	1.007	1.0018 (.006)	.25 (.80)
	Child	.04 (.06)	1.008	1.007	1.0018 (.006)	.25 (.80)
	Adolescent	.18 (.11)	1.012	1.011	.997 (.007)	<0
Anxiety	Largest	-.15 (.08)	0.999	0.999	1.017 (.007)	NA*
	Child	-.15 (.09)	0.999	1.001	1.016 (.006)	20.42 (7.95)
	Adult	-.03 (.10)	1.002	0.997	0.99 (.006)	NA*
Autistic traits	Largest	-.08 (.06)	1.023	1.021	1.032 (.006)	1.54 (.303)
	Child	-.09 (.07)	1.02	1.022	1.034 (.006)	1.52 (.276)
	Adult	.09 (.15)	1.005	1.008	1.003 (.006)	.30 (.79)
Depression	Largest	.02 (.04)	1.023	1.016	1.013 (.006)	.80 (.40)
	Child	.03 (.09)	1.029	1.024	1.021 (.007)	.88 (.28)
	Adult	.03 (.06)	1.026	1.014	1.010 (.007)	.72 (.46)
Neuro	Adult	-.04 (.09)	1.471	1.208	1.214 (.006)	1.03 (.03)
PLE	Adolescent	-.17 (.26)	1.005	1.011	1.017 (.006)	1.59 (.59)
Wellbeing	Adult	-.07 (.06)	1.005	1.008	1.017 (.007)	2.12 (.82)

Notes: Largest: largest available sample, obtained by selecting the largest sample from each study, irrespective of age group. Child: data from studies where participants were aged 5-12 years old; Adolescent: data from studies where participants were aged 13-18 years old; Adult: data from studies where participants were aged >18 years old. * mean chi^2 < 1

6. MR analysis

It has been previously speculated that environmental sensitivity may relate to polygenic liability rather than single loci due to the environment interacting with a polygenic biological component[32]. To test this hypothesis, we used a two-sample summary data Mendelian randomization to estimate the influence of genetic liability of psychological phenotypes on their environmental sensitivity. Mendelian randomization uses genetic variants as instrumental variables for the exposure of interest. Three assumptions define instrumental variables. First, relevance: the instrument must be associated with the exposure. Second, independence, there must be no uncontrolled confounders of the instrument-outcome association, and third, the exclusion restriction, that the instruments only affect the outcome via the exposure of interest. We selected independent (LD=10,000kb, R^2=0.001) genetic variants associated with main effect GWAS of the phenotypes in our study and used the inverse variance weighted estimator to estimate the effect of genetic liability to these phenotypes on phenotypic variability, using the TwoSampleMR package[33]. Because depression is strongly genetically correlated with anxiety, but there are no well-powered GWASs for anxiety, we performed a similar analysis for variance in anxiety but using the 102 variants for depression. Here, the main effects for anxiety at each of the 102 variants were obtained from a GWAS in UK Biobank for self-reported anxiety measures[34]. Finally, we also tested educational attainment[35]and body mass index. Table S14 shows the results.

Table S14: Influence of disease liability on environmental sensitivity.

GWAS Phenotype	MZ differences GWAS	nsnp	beta	se	pval
ADHD [36]	ADHD-Adult	27	-0.01	0.06	0.93
	ADHD-largest	27	0.11	0.04	0.02
	ADHD-child	27	0.11	0.04	0.02
ASD [37]	ASD-adult	9	-0.10	0.16	0.55
	ASD-largest	10	0.15	0.08	0.08
	ASD-Child	10	0.16	0.09	0.09
Depression [38]	Depression-adult	95	1.45	0.29	2.60E-06
	Depression-largest	95	0.65	0.26	0.015
	Depression-child	95	0.17	0.35	0.632
Neuroticism [39]	Neuroticism	110	-0.04	0.02	0.041
schizophrenia [40]	PLE	215	-0.03	0.04	0.562
Wellbeing [41]	Wellbeing	3	-1.41	0.08	0.036
BMI [40]	Depression-adult	458			
Educational attainment [35]	Depression-adult	74			

7. Evaluation of various data generating models for MZ differences

Many vQTL methods are liable to bias due to main additive effects. The MZ difference approach aims to protect against this problem but could plausibly be biased too under various analytical regimes, particularly when the trait is not normally distributed as is the case for the psychological traits in this study.
We conducted simulations to evaluate the sensitivity of the MZ difference design to this problem, using the following genetic liability model

$$l_i = \beta_{g1} g_{i,1} + \beta_e e_i + \beta_{g2} e_i g_{i,2} + v_i + \beta_f f + \epsilon_i$$

Where

$$v_i \sim N(0, \beta_3 g_{i,3})$$

and each g is a centred biallelic SNP with allele frequency 0.4, $e \sim N(0,1)$ is an environmental variable, $f \sim N(0,1)$ is a covariate and $\epsilon_i \sim N(0,1)$ is a residual error term.

- g_1 = Main effect that should have no influence in the MZ difference model
- g_2 = GxE effect that has no main effect and should have an influence in the MZ difference model
- g_3 = Variance heterogeneity effect that has no main effect and should have an influence in the MZ difference model

The liability l_i is then transformed in various ways to evaluate how sensitive the MZ difference model is to returning unexpected associations for g_1, g_2, g_3.

- Normal liability = l
- Normal liability squared = l^2 – creates skewness
- Normal liability squared (INT) = inverse normal transformation of l^2
- Exponentiated liability = e^l = the link model for generating skewed count data
- Exponentiated liability (INT) = inverse normal transformation of e^l
- Poisson of liability = Transformation of liability to score similarly distributed to those from standardised psychological phenotype scoring, $y \sim Pois(e^l)$
- Poisson of liability (INT) = inverse normal transformation of y
- Residuals of Poisson = residual of y after adjusting for covariate f
- Residuals of Poisson (INT) = inverse normal transformation of residual of y after adjusting for covariate f

We hypothesise that the most similar data generating model to the phenotypes used in our study is represented by the Residuals of Poisson (INT) model, though the true data generating model of complex psychological scores is not well understood.
The simulation results in **Figure S18** indicate that departure from normality can introduce bias (inflated effect estimates) in the MZ difference model, and that transformation to score data can attenuate variance effects. Residuals of the scores behave as expected, with little evidence of bias from the main effects and associations remaining for variance effects.

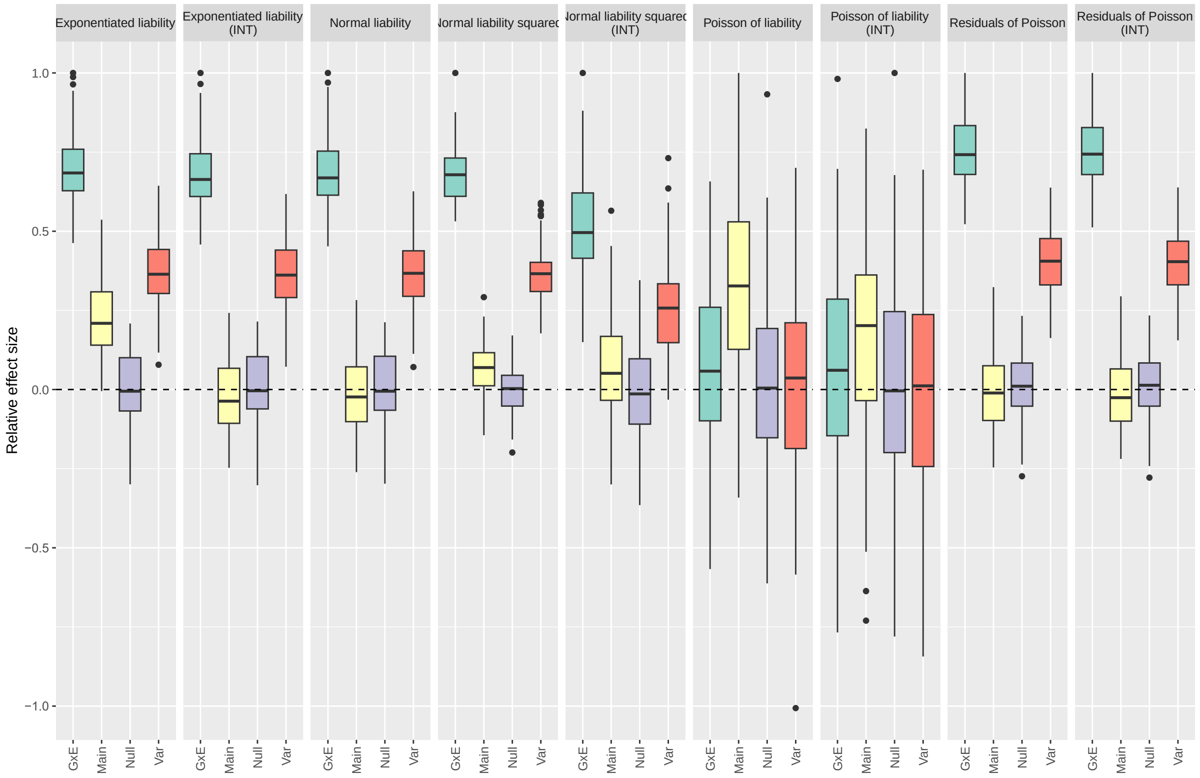

Figure S18: Evaluation of the MZ difference model for different genetic mechanisms

Evaluation of the MZ difference model for different genetic mechanisms (x axis) and different data generating models for the phenotype (columns of boxes). We expect the MZ difference model to have a relative effect size (y-axis) of 0 for the Main and Null genetic factors, and to have a positive effect for the GxE and variance heterogeneity (Var) models. Theory tends to focus on a perfectly normal liability (3rd column) which shows the model behaving as expected. Several models can give highly unexpected results, for example some of the Poisson based models. Though the underlying data generating model is unknown for the empirical analysis, the most likely is the Residuals of the Poisson (INT) model, which appears to behave as expected in the MZ difference model.

8. Funding, acknowledgements, and ethical approvals

The **Danish Twin Registry** has been supported by The National Program for Research Infrastructure 2007 (grant no. 09-063256), the Danish Agency for Science Technology and Innovation, and the US National Institute of Health (P01 AG08761). Genotyping was conducted by the SNP&SEQ Technology Platform, Science for Life Laboratory, Uppsala, Sweden (http://snpseq.medsci.uu.se/genotyping/snp-services/) and supported by NIH R01 AG037985 (Pedersen). Written informed consents were obtained from all participants. Collection and use of biological material and survey information were approved by the Regional Committees on **Health Research Ethics** for Southern Denmark, and the study is registered in SDU's internal list (notification no. 11.059) and complies with the rules in the General Data Protection Regulation.

Phenotype and genotype data collection in **Finnish Twin Cohort** has been supported by the Wellcome Trust Sanger Institute, the Broad Institute, ENGAGE – European Network for Genetic and Genomic Epidemiology, FP7-HEALTH-F4-2007, grant agreement number 201413, National Institute of Alcohol Abuse and Alcoholism (grants AA-12502, AA-00145, and AA-09203 to R J Rose; AA15416 and K02AA018755 to D M Dick; R01AA015416 to Jessica Salvatore) and the Academy of Finland (grants 100499, 205585, 118555, 141054, 264146, 308248, 312073, 336823, and 352792 to JKaprio). Informed consent was obtained before the beginning of the FTC studies in 1975, and upon every contact with the study subjects. When clinical investigations were undertaken with sampling of biological material, written informed consent was obtained. **Ethics approval** of these procedures was provided in multiple studies, the last one on the transfer of all available DNA samples and genotypes and the associated phenotypes to the THL Biobank by the Hospital District of Helsinki and Uusimaa ethics board in 2018 (#1799/2017). We wish to thank all the twins and their family members for their contributions over many decades. We also thank the many staff members who have contributed to data collection and data management.

Murcia Twin Registry is funded by Ministerio de Ciencia, Innovación y Universidades – Spain (RTI2018-095185-B-I00) co-funded by European Regional Development Fund (FEDER). Informed consent was obtained from all participants. The registry procedures have been approved by the University of **Murcia Research Ethics Committee.**

Netherlands Twin Register is funded by the Netherlands Organization for Scientific Research (NWO): NWO-GROOT 480-15-001/674. Ethical approval was provided by Central Ethics Committee on Research Involving Human Subjects of the VU University Medical Centre, Amsterdam, an Institutional Review Board certified by the US Office of Human Research Protections (IRB number IRB-2991 under Federal-wide Assurance-3703; IRB/institute codes, NTR 03-180).
We warmly thank the Netherlands Twin Register participants for their contributions.

Older Australian Twins Study has been funded by a National Health & Medical Research Council (NHMRC) and Australian Research Council (ARC) Strategic Award Grant of the Ageing Well, Ageing Productively Program (401162); NHMRC Project (seed) Grants (1024224, 1025243); NHMRC Project Grants (1045325, 1085606); and NHMRC Program Grants (568969,

1093083). OATS was facilitated through access to Twins Research Australia, a national resource supported by a Centre of Research Excellence Grant (1079102) from the National Health and Medical Research Council. OATS was approved by the human research ethics committees of the Australian Twin Registry, University of New South Wales, University of Melbourne, Queensland Institute of Medical Research and the South-eastern Sydney and Illawarra Area Health Service. Written informed consent was provided by all participants. We thank the participants for their time and generosity in contributing to this research. We acknowledge the contribution of the OATS research team (https://cheba.unsw.edu.au/project/older-australian-twins-study) to this study.

Queensland Institute of Medical Research (QIMR) studies have been supported from multiple sources: National Health and Medical Research Council (901061, 950998, 241944, 389875, 389891, 552485, 496682, 1009064, 552485, 496739, 1031119, 1049894, 1069141, 1086683, 1095227), Australian Research Council (A79600334, A79801419, A79906588, DP0212016, DP0343921), Human Frontiers Science Program (RG0154/1998-B), the Young and Well Cooperative Research Centre, which was established and funded under the Australian Government's Cooperative Research Centres Program. Phenotypic data were collected during a series of longitudinal studies of Australian twins and their families. These studies were approved by the QIMR Berghofer Medical Research Institute Human Research Ethics Committee and the storage of the data follows national regulations regarding personal data protection. All participants provided informed consent.

Swedish Twin registry is funded by Swedish Research Council (2017-0064); Ragnar Söderberg Foundation (E9/11); Swedish Research Council (421-2013-1061). The Swedish Twin Registry is managed by Karolinska Institutet and receives funding through the Swedish Research Council under the grant no 2017-00641. PsychChip GWAS genotyping and collaborative work supported in part by the National Institutes of Health/National Institute on Aging grants R01 AG037985, R01 AG059329, and R01 AG060470 and DNA extraction by grants R01 AG17561 and R01 AG028555. Harmony was supported by grant R01 AG08724. OCTO-Twin was supported by grant R01 AG08861. Gender was supported by the MacArthur Foundation Research Network on Successful Aging, The Axel and Margaret Axson Johnson's Foundation, The Swedish Council for Social Research, and the Swedish Foundation for Health Care Sciences and Allergy Research. SATSA was supported by grants R01 AG04563, R01 AG10175, the John D. and Catherine T. MacArthur Foundation Research Network on Successful Aging, the Swedish Council For Working Life and Social Research (FAS) (97:0147:1B, 2009-0795) and Swedish Research Council (825-2007-7460, 825-2009-6141). The research was also supported by Riksbankens Jubileumsfond (P18-0782:1) and the Swedish Research Council (2019-00244). The different twin studies held separate approvals by the regional **ethical review** board in Stockholm (SATSA: Dnr 80:80, 84:61; 93:226, 98:319, and 2010/657-31/3; OCTO-Twin: Dnr 98:380; HARMONY: Dnr: 97:051 and Dnr 2007/151-31/4). Ethical approval for the PSYCH, TwinGene and YATSS cohorts was given by the Uppsala Ethical Review Authority (nr: 2019-06066). All participants provided informed consents. We acknowledge The Swedish Twin Registry for access to data. The content of this manuscript is solely the responsibility of the authors and does not necessarily represent the official views of the National Institutes of Health/National Institute on Aging.

Twins Early Development (TEDS) is supported by the UK Medical Research Council (MR/V012878/1 and previously MR/M021475/1). Ethical approval for TEDS has been provided by the King's College London **Ethics Committee** (reference: PNM/09/10–104). Written informed consent was obtained prior to each wave of data collection from parents and from twins themselves from age 16 onwards. We gratefully acknowledge the ongoing contribution of the participants in the Twins Early Development Study (TEDS) and their families.

TwinsUK is funded by the Wellcome Trust, Medical Research Council, Versus Arthritis, European Union Horizon 2020, Chronic Disease Research Foundation (CDRF), Zoe Ltd, the National Institute for Health and Care Research (NIHR) Clinical Research Network (CRN) and Biomedical Research Centre based at Guy's and St Thomas' NHS Foundation Trust in partnership with King's College London. This study was carried out under TwinsUK BioBank ethics, approved by North West – Liverpool Central Research Ethics Committee (REC reference 19/NW/0187), IRAS ID 258513. This approval supersedes earlier approvals granted to TwinsUK by the St Thomas' Hospital Research Ethics Committee, later London – Westminster **Research Ethics Committee** (REC reference EC04/015), which have now been subsumed within the TwinsUK BioBank.

9. References

1. Bulik-Sullivan, B.K. *et al.* LD Score regression distinguishes confounding from polygenicity in genome-wide association studies. *Nature Genetics* **47**, 291 (2015).
2. Howe, L.J. *et al.* Within-sibship genome-wide association analyses decrease bias in estimates of direct genetic effects. *Nat Genet* **54**, 581-592 (2022).
3. Sachdev, P.S. *et al.* A comprehensive neuropsychiatric study of elderly twins: the Older Australian Twins Study. *Twin Res Hum Genet* **12**, 573-82 (2009).
4. Sachdev, P.S. *et al.* Cognitive functioning in older twins: the Older Australian Twins Study. *Australas J Ageing* **30 Suppl 2**, 17-23 (2011).
5. Lee, T. *et al.* Leisure activity, health, and medical correlates of neurocognitive performance among monozygotic twins: the Older Australian Twins Study. *J Gerontol B Psychol Sci Soc Sci* **69**, 514-22 (2014).
6. Finkel, D. & Pedersen, N. Processing Speed and Longitudinal Trajectories of Change for Cognitive Abilities: The Swedish Adoption/Twin Study of Aging. *Neuropsychol Dev Cogn B Aging Neuropsychol Cogn* **11**, 325-345 (2004).
7. Gold, C.H., Malmberg, B., McClearn, G.E., Pedersen, N.L. & Berg, S. Gender and health: a study of older unlike-sex twins. *J Gerontol B Psychol Sci Soc Sci* **57**, S168-76 (2002).
8. McClearn, G.E. *et al.* Substantial genetic influence on cognitive abilities in twins 80 or more years old. *Science* **276**, 1560-3 (1997).
9. Gatz, M. *et al.* Complete ascertainment of dementia in the Swedish Twin Registry: the HARMONY study. *Neurobiology of Aging* **26**, 439-47 (2005).
10. Jinks, J.L. & Fulker, D.W. Comparison of the biometrical genetical, MAVA, and classical approaches to the analysis of the human behavior. *Psychological Bulletin* **73**, 311-349 (1970).
11. Winkler, T.W. *et al.* Quality control and conduct of genome-wide association meta-analyses. *Nat Protoc* **9**, 1192-212 (2014).
12. Willer, C.J., Li, Y. & Abecasis, G.R. METAL: fast and efficient meta-analysis of genomewide association scans. *Bioinformatics* **26**, 2190-1 (2010).
13. Watanabe, K., Taskesen, E., van Bochoven, A. & Posthuma, D. Functional mapping and annotation of genetic associations with FUMA. *Nature Communications* **8**, 1826 (2017).
14. de Leeuw, C.A., Mooij, J.M., Heskes, T. & Posthuma, D. MAGMA: Generalized Gene-Set Analysis of GWAS Data. *PLOS Computational Biology* **11**, e1004219 (2015).
15. Kichaev, G. *et al.* Leveraging Polygenic Functional Enrichment to Improve GWAS Power. *Am J Hum Genet* **104**, 65-75 (2019).
16. Mahajan, A. *et al.* Fine-mapping type 2 diabetes loci to single-variant resolution using high-density imputation and islet-specific epigenome maps. *Nat Genet* **50**, 1505-1513 (2018).
17. Pietzner, M. *et al.* Mapping the proteo-genomic convergence of human diseases. *Science* **374**, eabj1541 (2021).
18. Schachter S Fau - Singer, J.E. & Singer, J.E. Cognitive, social, and physiological determinants of emotional state. *Psychological review* **69**, 379–399 (1962).
19. Onore, C., Van de Water, J. & Ashwood, P. Decreased Levels of EGF in Plasma of Children with Autism Spectrum Disorder. *Autism Research and Treatment* **2012**, 205362 (2012).

20. Toyoda, T. *et al.* SNP analyses of growth factor genes EGF, TGFβ-1, and HGF reveal haplotypic association of EGF with autism. *Biochemical and Biophysical Research Communications* **360**, 715-720 (2007).
21. Suzuki, K. *et al.* Decreased serum levels of epidermal growth factor in adult subjects with high-functioning autism. *Biological Psychiatry* **62**, 267-269 (2007).
22. Najafi, M. & Calvert, P.D. Transport and localization of signaling proteins in ciliated cells. *Vision Res* **75**, 11-8 (2012).
23. Baselmans, B.M.L. *et al.* Multivariate genome-wide analyses of the well-being spectrum. *Nat Genet* **51**, 445-451 (2019).
24. Thorp, J.G. *et al.* Symptom-level modelling unravels the shared genetic architecture of anxiety and depression. *Nature human behaviour* **5**, 1432-1442 (2021).
25. Thorp, J.G. *et al.* Symptom-level modelling unravels the shared genetic architecture of anxiety and depression. *Nat Hum Behav* **5**, 1432-1442 (2021).
26. Nagel, M., Watanabe, K., Stringer, S., Posthuma, D. & van der Sluis, S. Item-level analyses reveal genetic heterogeneity in neuroticism. *Nat Commun* **9**, 905 (2018).
27. Lander, G.C. *et al.* Complete subunit architecture of the proteasome regulatory particle. *Nature* **482**, 186-191 (2012).
28. Hoeller, D. & Dikic, I. How the proteasome is degraded. *Proceedings of the National Academy of Sciences* **113**, 13266-13268 (2016).
29. Howes, O., McCutcheon, R. & Stone, J. Glutamate and dopamine in schizophrenia: An update for the 21st century. *Journal of Psychopharmacology* **29**, 97-115 (2015).
30. Schwarz, D.S. & Blower, M.D. The endoplasmic reticulum: structure, function and response to cellular signaling. *Cell Mol Life Sci* **73**, 79-94 (2016).
31. Sakaue, S. *et al.* A cross-population atlas of genetic associations for 220 human phenotypes. *Nat Genet* **53**, 1415-1424 (2021).
32. Coleman, J.R.I. *et al.* Genome-wide gene-environment analyses of major depressive disorder and reported lifetime traumatic experiences in UK Biobank. (2020).
33. Hemani, G.A.-O. *et al.* The MR-Base platform supports systematic causal inference across the human phenome. LID - 10.7554/eLife.34408 [doi] LID - e34408. (2018).
34. Dönertaş, H.M., Fabian, D.K., Valenzuela, M.F., Partridge, L. & Thornton, J.M. Common genetic associations between age-related diseases. (2021).
35. Okbay, A. *et al.* Genome-wide association study identifies 74 loci associated with educational attainment. *Nature* **533**, 539-42 (2016).
36. Demontis, D. *et al.* Genome-wide analyses of ADHD identify 27 risk loci, refine the genetic architecture and implicate several cognitive domains. *Nature Genetics* **55**, 198-208 (2023).
37. Grove, J. *et al.* Identification of common genetic risk variants for autism spectrum disorder. *Nat Genet* **51**, 431-444 (2019).
38. Howard, D.M. *et al.* Genome-wide meta-analysis of depression identifies 102 independent variants and highlights the importance of the prefrontal brain regions. *Nat Neurosci* **22**, 343-352 (2019).
39. Loh, P.R., Kichaev, G., Gazal, S., Schoech, A.P. & Price, A.L. Mixed-model association for biobank-scale datasets. *Nat Genet* **50**, 906-908 (2018).
40. Trubetskoy, V. *et al.* Mapping genomic loci implicates genes and synaptic biology in schizophrenia. *Nature* **604**, 502-508 (2022).

41. Okbay, A. *et al.* Genetic variants associated with subjective well-being, depressive symptoms, and neuroticism identified through genome-wide analyses. *Nature Genetics* **48**, 624-633 (2016).
