# Supplementary material for: Genetics of monozygotic twins reveals the impact of environmental sensitivity on psychiatric and neurodevelopmental phenotypes
Source: Nat Hum Behav. 2025 Jun 10;9(8):1683–96. doi: 10.1038/s41562-025-02193-7 (PMC12367547; doi:10.1038/s41562-025-02193-7)
Supplement: Supplementary file 2 — Reporting Summary [file 41562_2025_2193_MOESM2_ESM.pdf]

nature portfolio

Corresponding author(s): Elham Assary
Last updated by author(s): 20/03/2025

Reporting Summary

Nature Portfolio wishes to improve the reproducibility of the work that we publish. This form provides structure for consistency and transparency in reporting. For further information on Nature Portfolio policies, see our Editorial Policies and the Editorial Policy Checklist.

Please do not complete any field with "not applicable" or n/a. Refer to the help text for what text to use if an item is not relevant to your study.
For final submission: please carefully check your responses for accuracy; you will not be able to make changes later.

Statistics

For all statistical analyses, confirm that the following items are present in the figure legend, table legend, main text, or Methods section.

n/a	Confirmed	
☐	☑	The exact sample size (n) for each experimental group/condition, given as a discrete number and unit of measurement
☐	☑	A statement on whether measurements were taken from distinct samples or whether the same sample was measured repeatedly
☐	☑	The statistical test(s) used AND whether they are one- or two-sided<br>*Only common tests should be described solely by name; describe more complex techniques in the Methods section.*
☐	☑	A description of all covariates tested
☐	☑	A description of any assumptions or corrections, such as tests of normality and adjustment for multiple comparisons
☐	☑	A full description of the statistical parameters including central tendency (e.g. means) or other basic estimates (e.g. regression coefficient) AND variation (e.g. standard deviation) or associated estimates of uncertainty (e.g. confidence intervals)
☐	☑	For null hypothesis testing, the test statistic (e.g. F, t, r) with confidence intervals, effect sizes, degrees of freedom and P value noted<br>*Give P values as exact values whenever suitable.*
☑	☐	For Bayesian analysis, information on the choice of priors and Markov chain Monte Carlo settings
☑	☐	For hierarchical and complex designs, identification of the appropriate level for tests and full reporting of outcomes
☑	☐	Estimates of effect sizes (e.g. Cohen's d, Pearson's r), indicating how they were calculated

Our web collection on statistics for biologists contains articles on many of the points above.

Software and code

Policy information about availability of computer code

Data collection	
Data analysis	The packages used in the study are: EasyQC (v. 23.8), METAL (2011 release), MAGMA (v1.08) within FUMA web application (v1.5.2), TwoSamle MR (v.0.5.9). Scripts for GWAS analyses are available here.

For manuscripts utilizing custom algorithms or software that are central to the research but not yet described in published literature, software must be made available to editors and reviewers. We strongly encourage code deposition in a community repository (e.g. GitHub). See the Nature Portfolio guidelines for submitting code & software for further information.

Data

Policy information about availability of data

All manuscripts must include a data availability statement. This statement should provide the following information, where applicable:

- Accession codes, unique identifiers, or web links for publicly available datasets
- A description of any restrictions on data availability
- For clinical datasets or third party data, please ensure that the statement adheres to our policy

Meta-analysed GWAS summary statistics from the current study are publicly available from OpenGWAS (https://gwas.mrcieu.ac.uk/). **Accession codes** for GWAS meta-analyses: ADHD symptoms MZ twin differences – adolescent: ieu-b-5146, child: ieu-b-5147; Anxiety symptoms MZ twin differences – largest: ieu-b-5148, adult: ieu-b-5149, child: ieu-b-5150; Autism spectrum disorder symptoms MZ twin differences- largest: ieu-b-5151, adult: ieu-b-5152, child: ieu-b-5153; Depression symptoms MZ twin differences- largest:ieu-b-5154, adult: ieu-b-5155, child: ieu-b-5156; Neuroticism MZ twin differences score:ieu-b-5157; Psychotic-like experiences MZ twin differences: ieu-b-5158; Subjective wellbeing MZ twin differences: ieu-b-5159.
Data from individual studies are not publicly available and are subject to strict access control, since the consent given by the participants does not allow for data storage on an individual level in repositories or journals. Access to these data requires specific approval from the relevant data access committees for each cohort.

Mapping and allele frequency reference files (all based on NCBI build 37) for 1000G phase1 version3, 1000G phase3 version5, and Haplotype Reference Consortium (HRC) are available via https://www.uni-regensburg.de/medizin/epidemiologie-praeventivmedizin/genetische-epidemiologie/software/index.html.

Research involving human participants, their data, or biological material

Policy information about studies with human participants or human data. See also policy information about sex, gender (identity/presentation), and sexual orientation and race, ethnicity and racism.

Reporting on sex and gender	The study reports total sample sizes as well as the number of females in the data used in the analyses. Data on sex were obtained via self-reports, parent reports (for children) and birth registry. No sex or gender-based analyses have been conducted due to low power. Findings do not apply to a specific sex or gender.
Reporting on race, ethnicity, or other socially relevant groupings	The study does not include reporting on ethnicity/race or other socially relevant groupings
Population characteristics	The sample consisted exclusively of monozygotic twins, registered in twin cohorts
Recruitment	The study did not involve participant recruitment as the data for analysis was already collected via participating twin cohorts. Different recruitment methods were used in each cohort, including recruitment through birth registries, hospital records and national registers. More details on recruitment for each participating cohort is provided in supplementary materials.
Ethics oversight	Ethical approval was obtained separately in each participating study. The details are presented in the manuscript under methods section.

Note that full information on the approval of the study protocol must also be provided in the manuscript.

Field-specific reporting

Please select the one below that is the best fit for your research. If you are not sure, read the appropriate sections before making your selection.

☐ Life sciences ☑ Behavioural & social sciences ☐ Ecological, evolutionary & environmental sciences

For a reference copy of the document with all sections, see nature.com/documents/nr-reporting-summary-flat.pdf

Life sciences study design

All studies must disclose on these points even when the disclosure is negative.

Sample size	
Data exclusions	
Replication	
Randomization	
Blinding	

Behavioural & social sciences study design

All studies must disclose on these points even when the disclosure is negative.

Study description	This was a genome-wide association study of quantitative data on seven psychological traits (attention deficit hyperactivity symptoms, autistic traits, anxiety and depression symptoms, psychotic-like experiences, neuroticism, and wellbeing), assessed in monozygotic twins along with genome-wide genetic data
Research sample	The sample consisted of monozygotic twins, with participants' age ranging between 7 to 70 years old across studies, with approximately half of the sample reported as being female. Mean age across studies, number of females per phenotype and total number of participants in the study are presented in Table 1 and per cohort information are presented in SI.
Sampling strategy	Stratified sampling procedure: analysis of existing data only from monozygotic twins registered in twin cohorts. The planned sample size was to collate the largest possible data on MZ twins with DNA data from existing datasets. The final sample size was determined by availability of genome-wide genetic and phenotypic data in twin cohorts who contributed to the study.
Data collection	No data was collected for the purpose of this study as it involved secondary data analysis of existing datasets. The data collection method varied by participating twin cohorts, including interviews, pen and paper questionnaires and online questionnaires.
Timing	Secondary analysis of GWAS data from participating cohorts were carried out between September 2020 to February 2022.
Data exclusions	Samples with genetic data missingness more than 5% and genetic data missing in more than 95% of the sample were excluded from analyses. Data that were available only for one twin from each pair were excluded, as the phenotype construction step (phenotype differences) requires data from both twins, as well as individuals with missing information on covariates such as sex and age to account for demographic effects
Non-participation	No participants were involved in the study, as this was secondary data analysis of existing datasets.
Randomization	Randomisation did not apply to this study design, as it was a correlational study of genetic and phenotypic data

Ecological, evolutionary & environmental sciences study design

All studies must disclose on these points even when the disclosure is negative.

Study description

Research sample

Sampling strategy

Data collection

Timing and spatial scale

Data exclusions

Reproducibility

Randomization

Blinding

Did the study involve field work? ☐ Yes ☐ No

Field work, collection and transport

Field conditions

Location

Access & import/export

Disturbance

Reporting for specific materials, systems and methods

We require information from authors about some types of materials, experimental systems and methods used in many studies. Here, indicate whether each material, system or method listed is relevant to your study. If you are not sure if a list item applies to your research, read the appropriate section before selecting a response.

Materials & experimental systems

n/a	Involved in the study
☑	☐ Antibodies
☑	☐ Eukaryotic cell lines
☑	☐ Palaeontology and archaeology
☑	☐ Animals and other organisms
☑	☐ Clinical data
☑	☐ Dual use research of concern
☑	☐

Methods

n/a	Involved in the study
☑	☐ ChIP-seq
☑	☐ Flow cytometry
☑	☐ MRI-based neuroimaging

Antibodies

Antibodies used

Validation

Eukaryotic cell lines

Policy information about cell lines and Sex and Gender in Research

Cell line source(s)

Authentication

Mycoplasma contamination

Commonly misidentified lines
(See ICLAC register)

Palaeontology and Archaeology

Specimen provenance

Specimen deposition

Dating methods

☐ Tick this box to confirm that the raw and calibrated dates are available in the paper or in Supplementary Information.

Ethics oversight

Note that full information on the approval of the study protocol must also be provided in the manuscript.

Animals and other research organisms

Policy information about studies involving animals; ARRIVE guidelines recommended for reporting animal research, and Sex and Gender in Research

Laboratory animals

Wild animals

Reporting on sex

Field-collected samples

Ethics oversight

Note that full information on the approval of the study protocol must also be provided in the manuscript.

Clinical data

Policy information about clinical studies
All manuscripts should comply with the ICMJE guidelines for publication of clinical research and a completed CONSORT checklist must be included with all submissions.

Clinical trial registration

Study protocol

Data collection

Outcomes

Dual use research of concern

Policy information about dual use research of concern

Hazards

Could the accidental, deliberate or reckless misuse of agents or technologies generated in the work, or the application of information presented in the manuscript, pose a threat to:

No | Yes

- [ ] [ ] Public health
- [ ] [ ] National security
- [ ] [ ] Crops and/or livestock
- [ ] [ ] Ecosystems
- [ ] [ ] Any other significant area

Experiments of concern

Does the work involve any of these experiments of concern:

No | Yes

- [ ] [ ] Demonstrate how to render a vaccine ineffective
- [ ] [ ] Confer resistance to therapeutically useful antibiotics or antiviral agents
- [ ] [ ] Enhance the virulence of a pathogen or render a nonpathogen virulent
- [ ] [ ] Increase transmissibility of a pathogen
- [ ] [ ] Alter the host range of a pathogen
- [ ] [ ] Enable evasion of diagnostic/detection modalities
- [ ] [ ] Enable the weaponization of a biological agent or toxin
- [ ] [ ] Any other potentially harmful combination of experiments and agents

Plants

Seed stocks

Novel plant genotypes

Authentication

ChIP-seq

Data deposition

- [ ] Confirm that both raw and final processed data have been deposited in a public database such as GEO.
- [ ] Confirm that you have deposited or provided access to graph files (e.g. BED files) for the called peaks.

Data access links
May remain private before publication.

Files in database submission

Genome browser session
(e.g. UCSC)

Methodology

Replicates

Sequencing depth

Antibodies

Peak calling parameters

Data quality

Software

Flow Cytometry

Plots

Confirm that:

- [ ] The axis labels state the marker and fluorochrome used (e.g. CD4-FITC).
- [ ] The axis scales are clearly visible. Include numbers along axes only for bottom left plot of group (a 'group' is an analysis of identical markers).
- [ ] All plots are contour plots with outliers or pseudocolor plots.
- [ ] A numerical value for number of cells or percentage (with statistics) is provided.

Methodology

Sample preparation

Instrument

Software

Cell population abundance

Gating strategy

- [ ] Tick this box to confirm that a figure exemplifying the gating strategy is provided in the Supplementary Information.

Magnetic resonance imaging

Experimental design

Design type

Design specifications

Behavioral performance measures

Imaging type(s)

Field strength

Sequence & imaging parameters

Area of acquisition

Diffusion MRI ☐ Used ☐ Not used

Preprocessing

Preprocessing software

Normalization

Normalization template

Noise and artifact removal

Volume censoring

Statistical modeling & inference

Model type and settings

Effect(s) tested

Specify type of analysis: ☐ Whole brain ☐ ROI-based ☐ Both

Statistic type for inference

(See Eklund et al. 2016)

Correction

Models & analysis

n/a	Involved in the study
☐	☐ Functional and/or effective connectivity
☐	☐ Graph analysis
☐	☐ Multivariate modeling or predictive analysis

Functional and/or effective connectivity

Graph analysis

Multivariate modeling and predictive analysis

This checklist template is licensed under a Creative Commons Attribution 4.0 International License, which permits use, sharing, adaptation, distribution and reproduction in any medium or format, as long as you give appropriate credit to the original author(s) and the source, provide a link to the Creative Commons license, and indicate if changes were made. The images or other third party material in this article are included in the article's Creative Commons license, unless indicated otherwise in a credit line to the material. If material is not included in the article's Creative Commons license and your intended use is not permitted by statutory regulation or exceeds the permitted use, you will need to obtain permission directly from the copyright holder. To view a copy of this license, visit http://creativecommons.org/licenses/by/4.0/
